# Mechanistic Studies of a Skatole-Forming Glycyl Radical Enzyme Suggest Reaction Initiation via Hydrogen Atom Transfer

**DOI:** 10.1021/jacs.1c13580

**Published:** 2022-06-15

**Authors:** Beverly Fu, Azadeh Nazemi, Benjamin J. Levin, Zhongyue Yang, Heather J. Kulik, Emily P. Balskus

**Affiliations:** † Department of Chemistry and Chemical Biology, Harvard University, Cambridge, Massachusetts 02138, United States; ‡ Howard Hughes Medical Institute, Harvard University, Cambridge, Massachusetts 02138, United States; § Department of Chemical Engineering, Massachusetts Institute of Technology, Cambridge, Massachusetts 02139, United States

## Abstract

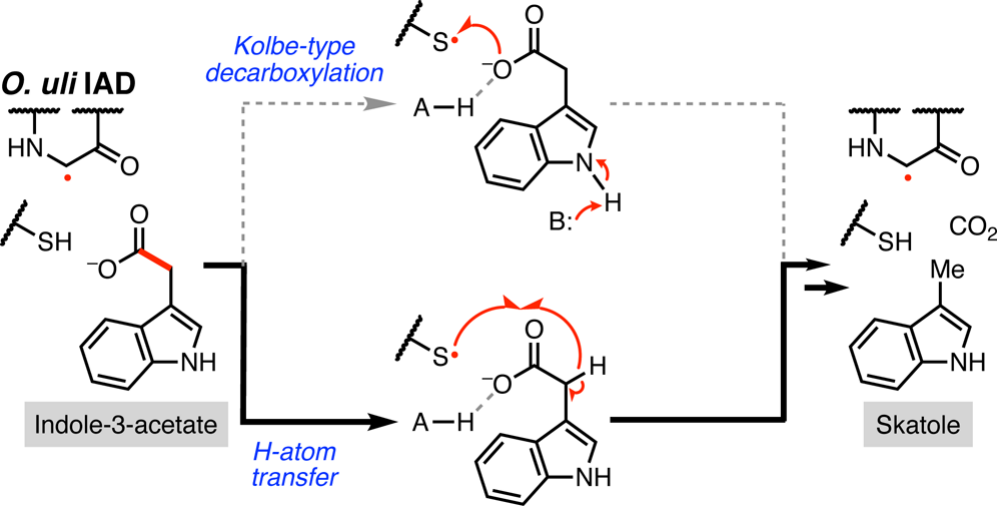

Gut microbial decarboxylation of amino acid-derived arylacetates is a chemically challenging enzymatic transformation which generates small molecules that impact host physiology. The glycyl radical enzyme (GRE) indoleacetate decarboxylase from *Olsenella uli* (*Ou* IAD) performs the non-oxidative radical decarboxylation of indole-3-acetate (I3A) to yield skatole, a disease-associated metabolite produced in the guts of swine and ruminants. Despite the importance of IAD, our understanding of its mechanism is limited. Here, we characterize the mechanism of *Ou* IAD, evaluating previously proposed hypotheses of: (1) a Kolbe-type decarboxylation reaction involving an initial 1-e^–^ oxidation of the carboxylate of I3A or (2) a hydrogen atom abstraction from the α-carbon of I3A to generate an initial carbon-centered radical. Site-directed mutagenesis, kinetic isotope effect experiments, analysis of reactions performed in D_2_O, and computational modeling are consistent with a mechanism involving initial hydrogen atom transfer. This finding expands the types of radical mechanisms employed by GRE decarboxylases and non-oxidative decarboxylases, more broadly. Elucidating the mechanism of IAD decarboxylation enhances our understanding of radical enzymes and may inform downstream efforts to modulate this disease-associated metabolism.

## Introduction

**Figure 1:**
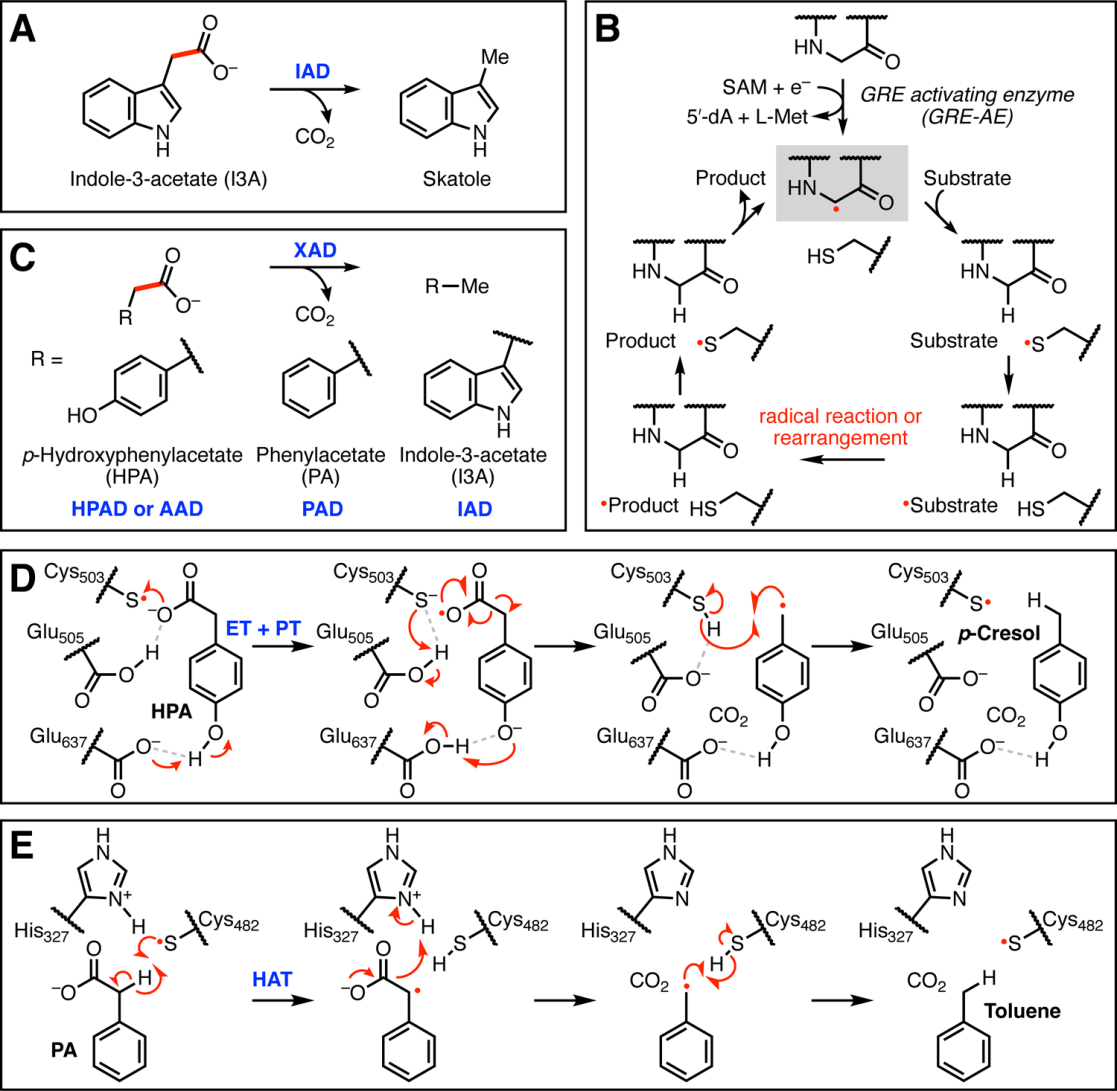
Four glycyl radical enzyme (GRE) decarboxylases have different proposed mechanisms. (A) Indoleacetate decarboxylase (IAD) converts indole-3-acetate (I3A) into skatole. (B) Generic GRE catalytic cycle involves a conserved catalytic Gly and Cys residue. (C) The X-acetate decarboxylases metabolize arylacetate substrates derived from aromatic amino acids. Hydroxyphenylacetate decarboxylase (HPAD) and arylacetate decarboxylase (AAD) metabolize *p*-hydroxyphenylacetate (HPA), while phenylacetate decarboxylase (PAD) metabolizes phenylacetate (PA). (D) Proposed Kolbe-type decarboxylation mechanism for HPAD. Residue numbering from *Clostridium scatologenes* HPAD. (E) Proposed H-atom transfer mechanism for PAD. An alternative possibility is generation of a carboxylate radical through solely 1-e^–^ transfer. Residue numbering from PAD isolated from sewage.

In the largely anoxic mammalian gastrointestinal tract, microbial enzymes often take advantage of radical-mediated mechanisms to accomplish challenging transformations.^1^ One frequently observed reaction class is radical-based decarboxylation. For example, *Clostridioides difficile* decarboxylates *p*-hydroxyphenylacetate (HPA) into *p*-cresol, a bacteriostatic catabolite that may provide a growth advantage for *C. difficile*.^2^ Other organisms decarboxylate indole-3-acetate (I3A) to generate skatole^3^ (Figure 1A), a disease-associated metabolite produced within swine and ruminant gut microbiomes.^4^ Accumulation of skatole in swine fat cells contributes to boar taint, in which pork meat acquires an offensive odor and taste. Oxidation of skatole in ruminant lungs generates a reactive, electrophilic metabolite that causes fog fever and eventual death by asphyxiation. In humans, gut bacterial-derived skatole is a pulmonary and hepatic toxin.^5^ Given the importance of these bacterial metabolites, gaining a mechanistic understanding of radical-based decarboxylases may inform downstream efforts to inhibit these deleterious microbial activities.

Decarboxylation of HPA and I3A is difficult to achieve through 2-e^–^ mechanisms, as the resultant negative charge generated upon CO_2_ release cannot be stabilized. Instead, arylacetate decarboxylation is catalyzed by members of the glycyl radical enzyme (GRE) family.^6^ These O_2_-sensitive enzymes use a conserved glycine-centered radical to initiate challenging chemical transformations.^7^ The stable, protein-based glycyl radical is installed post-translationally by a dedicated partner activating enzyme (GRE-AE) belonging to the radical *S*-adenosyl-l-methionine (rSAM) superfamily.^8^ The GRE-AE reductively cleaves SAM at the SAM-binding [Fe_4_S_4_]^+^ cluster, generating a reactive 5′-deoxyadenosyl radical (5′-dA•) species that abstracts a hydrogen atom (H-atom) from the conserved Gly of the GRE peptide backbone.^6,9−11^ Biochemical experiments and structural data have uncovered shared mechanistic features of GREs (Figure 1B).^12^ The glycyl radical is proposed to first abstract an H-atom from a neighboring conserved Cys residue.^13,14^ Although a thiyl radical has never been directly detected, its involvement is widely accepted. The thiyl radical reacts with the substrate via H-atom transfer (HAT) or e^–^ transfer (ET). The resultant substrate-based radical then enables downstream bond cleavage or rearrangement events that generate a product-centered radical. This intermediate re-abstracts an H-atom from the Cys residue, providing product and regenerating the catalytic Gly radical.^12^ Using this cycle, GREs catalyze diverse radical-mediated chemical reactions including C–C, C–O, C–N, and C–S bond cleavage and formation.^3,12,15−18^

Four GRE decarboxylases have been biochemically characterized to date: hydroxyphenylacetate decarboxylase (HPAD),^19^ arylacetate decarboxylase (AAD),^18^ phenylacetate decarboxylase (PAD),^17^ and indoleacetate decarboxylase (IAD)^3^ (Figure 1C). HPAD, the most extensively characterized decarboxylase, catalyzes *p*-cresol formation from HPA.^20^ More recently, AAD was reported to also decarboxylate HPA despite lacking key auxiliary subunits and active site residues conserved in HPAD. Although a structure of AAD co-crystallized with HPA has been published, the density attributed to HPA was remote from the catalytic Cys and thus provides limited mechanistic information.^18^ PAD was first reported to convert phenylacetate (PA) into toluene in cell-free extracts,^21^ and its activity was subsequently validated *in vitro*.^17^ Finally, IAD was reported in 2018 to decarboxylate I3A to give skatole and has been preliminarily characterized.^3^ Although minor modifications to the aryl rings are typically tolerated, each decarboxylase is specific to its native substrate.^2,3,17^

The GRE decarboxylases may use distinct radical decarboxylation mechanisms. Current evidence suggests that HPAD employs a Kolbe-type decarboxylation mechanism (Figure 1D), based on the orientation of HPA in a co-crystal structure and hybrid quantum chemical/molecular mechanical calculations.^22^ In short, 1-e^–^ oxidation of the carboxylate of HPA by the active site thiyl radical is proposed to generate a substrate-based carboxyl radical. This step is thought to be coupled to deprotonation of the phenol of HPA by Glu637.^22^ The Cys thiolate is then protonated by Glu505. Radical decarboxylation is driven by re-protonation of the phenolate by Glu637, generating a benzylic radical, which then abstracts an H-atom from the Cys to form *p*-cresol. Notably, PA, the substrate of PAD, lacks the *para*-hydroxy functional group proposed to enable the Kolbe-type decarboxylation, so a different mechanism must be operant.^17^ One alternative proposal is that PAD employs a Kolbe-type decarboxylation mechanism with the initial step involving ET from the carboxylate of PA without a coupled proton transfer (PT). A second possibility starts with HAT from the methylene carbon of PA to generate an α-carbon-centered radical. 2-e^–^ decarboxylation, followed by HAT from the conserved Cys, produces toluene (Figure 1E). This latter proposal is supported by studies with methylene-substituted PA analogs and density functional theory calculations,^23^ but these results do not rule out the alternative Kolbe-type decarboxylation mechanism.

To date, it is unclear which of these potential mechanisms is employed in I3A decarboxylation by IAD. Liu et al. initially discovered IAD from *Olsenella scatoligenes* (*Os* IAD) and biochemically verified it is a GRE.^3^ They reported Michaelis–Menten kinetics of wild-type (WT) *Os* IAD (Table S1) and bioinformatically identified conserved active site residues that could potentially participate in a Kolbe-type decarboxylation. However, this work did not include mechanistic experiments.

Here, we describe our efforts to study the mechanism of I3A decarboxylation by IAD from *Olsenella uli* (*Ou* IAD). Combining evidence from site-directed mutagenesis, kinetic isotope effect (KIE) experiments with deuterated substrates, deuterium-incorporation studies in D_2_O, and computational modeling of reaction intermediates, we propose that *Ou* IAD performs decarboxylation of I3A by first generating an α-carbon-centered radical intermediate, reminiscent of the proposed mechanism for PAD. Elucidation of the details of this radical-based decarboxylation reaction enhances our understanding of the chemical strategies used for enzymatic non-oxidative radical decarboxylations and lays the foundation for developing mechanism-based inhibitors for these disease-associated enzymes.

## Results

### Biochemical Verification of *Ou* IAD Activity

**Figure 2:**
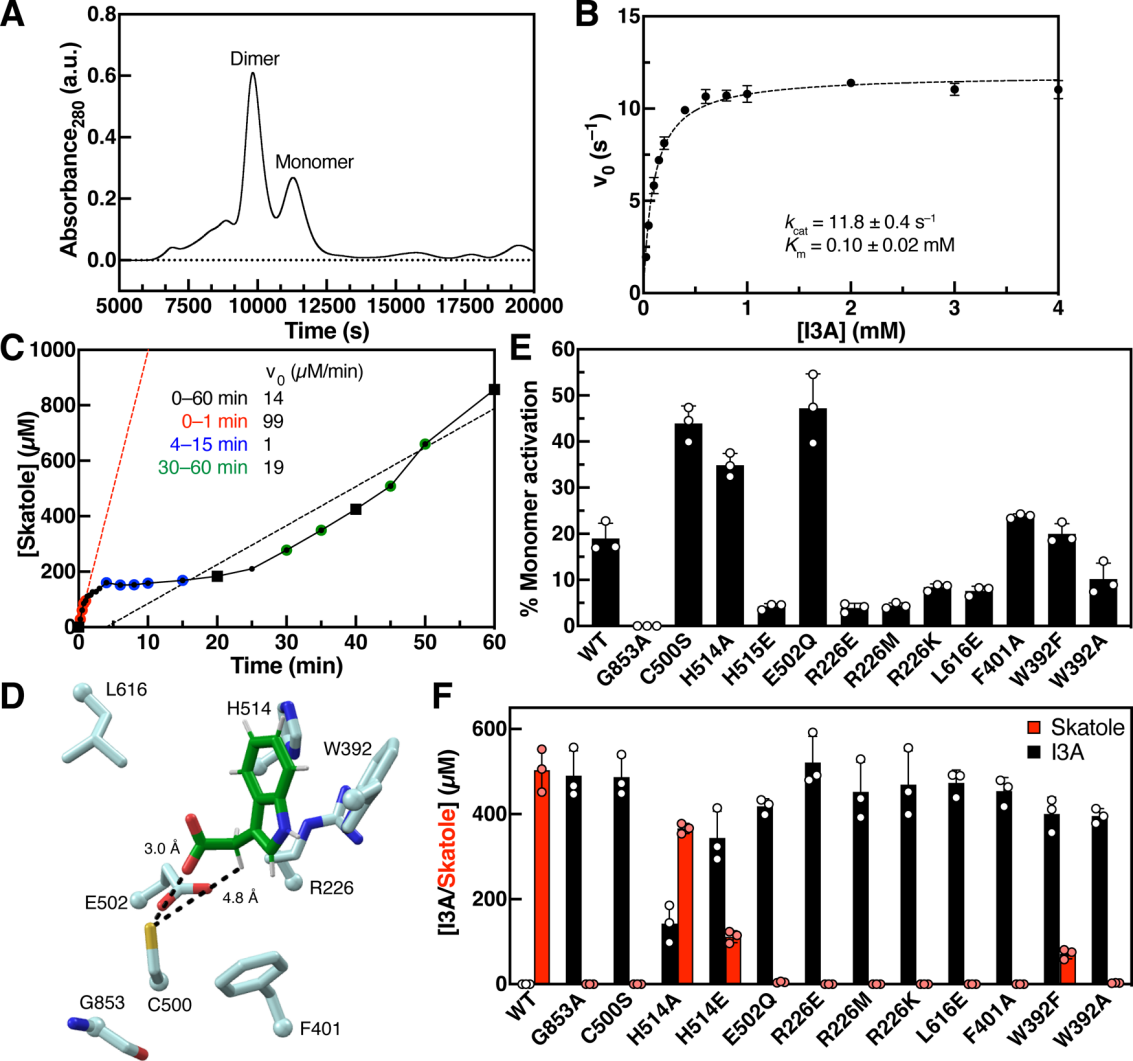
Biochemical characterization of *Ou* IAD reveals it is a GRE. (A) Size exclusion chromatography (SEC) trace of *Ou* IAD. (B) Michaelis–Menten kinetics of WT *Ou* IAD using initial rates measured in the first 60 s. Data are mean ± SD (*n* = 3). Fitted parameters are mean ± SE (*n* = 3) as derived from nonlinear curve fitting to the Michaelis–Menten equation. Assay performed twice on separate days. (C) Full progress curve of *Ou* IAD over the course of an hour (*n* = 1). Experiment repeated three times on separate days. Determination of initial rates (*v*_0_) differs based on time points chosen. (D) Homology model generated with SWISS-MODEL using *Cs* HPAD (33.1% amino acid ID) as a template and docked with I3A. (E) Glycyl radical formation of active site point variants. Data are mean ± SD (*n* = 3). (F) End-point skatole production of wild-type (WT) and *Ou* IAD variants. Data are mean ± SD (*n* = 3).

We independently noted that the decarboxylation of I3A parallels that of HPA and PA and hypothesized the involvement of a GRE. We identified putative IAD- and IAD-AE-encoding genes in the genome of *O. uli* DSM 7084^T^, a known skatole-producing bacterium isolated from the dental plaque of periodontitis patients. *Ou* IAD shares 89% amino acid (aa) ID to *Os* IAD. Both *Ou* IAD and IAD-AE were purified (Figure S1A), and size exclusion chromatography (SEC) of *Ou* IAD indicated that it is a mixture of oligomeric states, with a putative dimer as the predominant form (70%) along with a putative monomer and higher order oligomers (Figure 2A). *Ou* IAD-AE eluted as a dark brown solution (4.23 ± 0.08 Fe and 5.3 ± 0.6 S per monomer) and had a UV–vis absorbance spectrum of a typical [Fe_4_S_4_]-containing protein with a shoulder around 410 nm that disappeared upon incubation with sodium dithionite, indicating reduction of the [Fe_4_S_4_]^2+^ to the +1 state (Figure S1B).

Incubation of *Ou* IAD/IAD-AE, SAM, dithiothreitol (DTT) and light-activated 5-deazariboflavin resulted in formation of a glycyl radical on *O*u IAD, as determined by electron paramagnetic resonance (EPR) spectroscopy (*g* = 2.0038, *A* = 1.4 mT) (Figure S1C). Spin quantification of the EPR spectrum indicated that 19 ± 2% of *Ou* IAD monomers contained a glycyl radical. We extensively optimized these conditions, screening various reductants, length of halogen lamp illumination, buffer components, enzyme ratios, and [FeS] reconstitution of *Ou* IAD-AE (Supporting Information Methods). When activated *Ou* IAD was incubated with I3A for 2.5 h, the substrate was stoichiometrically converted to skatole, as detected by ultra-performance liquid chromatography-tandem mass spectrometry (UPLC–MS/MS) and confirmed by comparison to authentic standards. The isolated monomeric and dimeric forms of *Ou* IAD were equally active in end-point assays (Figure S1D), although it is unclear if the two species interconvert under our assay conditions. The oligomeric mixture was used for experimental work going forward. Turnover required all assay components and an anaerobic environment (Figure S1E). Importantly, only samples illuminated with a 500 W halogen lamp produced quantifiable amounts of glycyl radical by EPR, although overnight incubations omitting 5-deazariboflavin resulted in complete skatole production, implying there was a small amount of IAD-AE isolated in the reduced form. This result is further corroborated by the fact that 5′-dA can only be detected when both 5-deazariboflavin and DTT reductants are included (Figure S1E). Alternative reductants sodium dithionite, acriflavine, and titanium(III) citrate resulted in less active *Ou* IAD. Incomplete activation, which results in a heterogeneous population of enzymes, is frequently observed for GREs. Altogether, these experiments verify that the *Ou* IAD/IAD-AE pair converts I3A into skatole.

### Michaelis–Menten Kinetics of WT *Ou* IAD

To further verify *Ou* IAD’s activity toward I3A, we determined its Michaelis–Menten kinetic parameters (*k*_cat_ = 11.8 ± 0.4 s^–1^, *K*_m_ = 0.10 ± 0.02 mM), using initial rates derived from the first 60 s and normalizing to the amount of glycyl radical formation (Figure 2B). Although the *K*_m_ is similar to that reported for *Os* IAD, the *k*_cat_ for *Ou* IAD is about 6 times faster. In addition, the catalytic efficiency (*k*_cat_/*K*_m_ = (1.18 ± 0.2) × 10^5^ s^–1^ M^–1^) of *Ou* IAD is on the same order as those reported for HPAD (Table S1). We attribute the difference in measured *k*_cat_ between IAD homologs to the time frame used for kinetic assays. *Ou* IAD progress curves showed an initial fast rate of product formation (0–5 min), followed by a slight plateau (5–20 min), and then a linear phase of slower product formation over the course of an hour (Figure 2C). As a result, the initial rate measured within the first min is much higher than that measured over an hour. Liu et al. confirmed progress curve linearity by measuring skatole production at solely 20, 40, and 60 min and determined initial rates using a single 10 min time point.^3^ Determination of *Ou* IAD kinetics with this method resulted in considerably lower parameters (*k*_cat_ = 0.95 ± 0.08 s^–1^, *K*_m_ = 0.09 ± 0.03 mM, *k*_cat_/*K*_m_ = (1.1 ± 0.4) × 10^4^ s^–1^ M^–1^), which are more comparable to those reported by Liu et al. (Figure S2A).

While we cannot yet explain this kinetic complexity, a likely contributing factor is the heterogeneity of the *Ou* IAD enzyme preparation, which contains a mixture of active and inactive enzyme, as well as different oligomeric states and conformations. As noted earlier, this is often observed for GREs. Compared to other biochemically characterized GREs, *Ou* IAD turnover rate (*k*_cat_) is low. However, its catalytic efficiency is on the higher end of the GREs (Table S1).

### Site-Directed Mutagenesis of Putative *Ou* IAD Active Site Residues

We generated an MSA of the GRE decarboxylases to identify active site residues conserved across IAD homologs (Figure S3A) and constructed protein prediction models of *Ou* IAD using a variety of programs^24−26^ (Figures 2D and S4). Although the overall predicted structures are similar (Figure S4A–C), the positions of active site residues without substrate (Figure S4D–F) and with I3A docked (Figure S4G–I) differ. Our models also differ from that previously generated by Liu et al.^3^ As the putative active site residues and I3A binding orientations are inconsistent across models, we decided to probe the active site using site-directed mutagenesis.

The analyses described above identified Gly853 and Cys500 as the universally conserved catalytic residues in *Ou* IAD. As previously noted,^3^ IAD has a conserved Glu502 proposed to act as the proton donor upon carboxylate oxidation and thiolate reduction. Conservation of this residue between IAD and HPAD would be consistent with IAD proceeding through a Kolbe-type decarboxylation mechanism. However, IAD does not have a second conserved Glu for simultaneous substrate deprotonation; our models predict that His514 and Leu616 are in this region of the active site. His514 was previously predicted to be in close proximity to the nitrogen atom of I3A and proposed to act as a base to facilitate substrate deprotonation in a Kolbe-type decarboxylation.^3^ However, the indole nitrogen of I3A is predicted to be considerably less acidic (p*K*_a_ = 16) than the phenol (p*K*_a_ = 10) of HPA.^27^ Other potentially important active site residues include Arg226, which could participate in a cation-π interaction with the indole ring of I3A, influencing substrate binding. IAD also has an aromatic amino acid (Phe401 or Tyr401) that may serve as a cap for the carboxylate group of I3A. Finally, the active site is predicted to contain a Trp residue (Trp392) that could form a π-stacking interaction with I3A.

To probe the importance of these putative active site residues, we expressed and purified *Ou* IAD variants (Figure S1A) and evaluated their ability to harbor a stable glycyl radical and produce skatole (Figure 2E,F). The oligomeric state of the mutants was not assessed by SEC. We verified that Gly853 and Cys500 are indeed the conserved Gly and Cys required for catalysis, as substitution of Gly853 with Ala generates inactive *Ou* IAD that can neither install a glycyl radical nor produce skatole, while the C500S variant can still harbor a glycyl radical but cannot not metabolize I3A. In contrast, both His514 variants still retain activity, albeit to a much lower degree. Compared to WT *Ou* IAD, the H514A variant has severely impaired kinetic parameters (*k*_cat_ = 0.18 ± 0.06 s^–1^, *K*_m_ = 7 ± 4 mM) (Figure S2B). As the *k*_cat_ has decreased by 66-fold and the *K*_m_ has also increased by 70-fold, substitution of His514 likely affects both substrate binding and catalysis. The retention of activity in His514 variants of IAD thus suggests this enzyme may not employ a Kolbe-type decarboxylation mechanism. Leu616 is predicted to be in close vicinity of His514, but substitution with Glu to mimic the HPAD active site renders IAD completely inactive. As there is no solved crystal structure of IAD, we cannot exclude the possibility that there is a residue that serves as a general base that we have yet to identify.

Substitution of Glu502 with Gln to mimic PAD greatly increases radical installation but abolishes skatole formation, demonstrating its critical role in catalysis. Similarly, although Arg226 can be substituted with a variety of residues (Glu, Met, and Lys) and still harbor low levels of the glycyl radical, these variants cannot produce skatole. Arg226 may be important for enabling substrate binding or transition state stabilization through interactions with the carboxylate or aromatic ring. Substitution of Phe401 with Ala also abolishes activity, perhaps indicating a role in constraining substrate binding. A hydrophobic residue is present at this position in GREs with diverse activities, suggesting a more generic or structural role in catalysis.^28^ Finally, substitution of Trp392 with Phe but not Ala preserved IAD activity. The tolerance of a different aromatic residue at this position suggests Trp392 may be involved in a π-stacking interaction with I3A. Although these analyses are based on predicted IAD structures, our results suggest we have identified residues important for substrate binding and catalysis.

### Kinetic Isotope Effects

Next, we examined the reactivity of *Ou* IAD toward various I3A analogs (Figure S5), particularly focusing on the methylene analogs α,α-Me_2_-I3A and racemic α-Me-I3A. If a HAT mechanism were operant, we would expect no reactivity with α,α-Me_2_-I3A and reduced reactivity with α-Me-I3A, assuming IAD is stereoselective. Both compounds would be expected to still undergo a Kolbe-type decarboxylation. However, neither compound was accepted by *Ou* IAD. Both compounds are partial competitive inhibitors in assays with I3A, suggesting they can bind and occupy the active site (Figure S6C,D). As neither analog was consumed, we cannot draw clear conclusions regarding mechanism. One potential reason for the lack of reactivity is that the methyl group may alter the binding of substrate and positioning of the methylene hydrogen atom or carboxylate in the active site. Alternatively, the methyl-substituted benzylic radical may be more stable than the hydrogen-substituted radical, making 2-e^–^ decarboxylation more energetically uphill (Table S9).^29^

**Figure 3:**
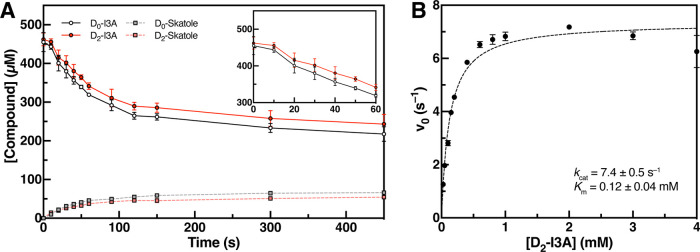
Assays with deuterated substrate analogs show a minor kinetic isotope effect (KIE) for I3A decarboxylation. (A) Substrate enrichment curves show that D_0_-I3A is consumed preferentially over D_2_-I3A. The inset is of the first 60 s. Data are mean ± SD (*n* = 3). Assay was performed twice on independent days (see Figure S8). (B) Michaelis–Menten kinetics of WT *Ou* IAD and D_2_-I3A using initial rates measured in first 60 s. Data are mean ± SD (*n* = 3). Fitted parameters are mean ± SE (*n* = 3) as derived from nonlinear curve fitting to the Michaelis–Menten equation. Assay was performed twice on independent days.

We next examined IAD activity toward deuterium-labeled substrates D_2_-I3A (two α-deuteria) and D_7_-I3A (perdeuterated except for indole N–H). If the Kolbe-decarboxylation mechanism were operant, we would not expect to observe any KIEs as no HAT or PT occurs at any of the deuterated sites. In contrast, the HAT mechanism should exhibit a KIE for both deuterated analogs, assuming generation of the substrate radical is the rate-limiting step. To assess this, we performed competition assays by incubating deuterium-labeled and unlabeled substrate (500 μM each) in a single reaction with *Ou* IAD and measured isotopologue consumption over the course of 25 min. Substrate isotopic enrichment was determined at 50% conversion. We found that D_2_-I3A exhibited a KIE of 1.14 ± 0.04 (Figure 3A) and D_7_-I3A exhibited a KIE of 1.2 ± 0.1 (Figure S7A). In contrast, competition assays with D_2_-I3A and D_7_-I3A revealed these substrates were consumed at similar rates (KIE of 1.03 ± 0.04), indicating the KIE observed for D_7_-I3A in competition with unlabeled I3A is due to the α-deuteria (Figure S7B). Given the small size of these KIEs and the heterogeneity of the enzyme preparations, we sought to use a complementary method to measure KIEs. Results of Michaelis–Menten kinetics with D_2_-I3A, normalized to the amount of glycyl radical formation (*k*_cat_ = 7.4 ± 0.5 s^–1^, *K*_m_ = 0.12 ± 0.04 mM, *k*_cat_/*K*_m_ = (6 ± 2) × 10^4^ s^–1^ M^–1^), showed a KIE of 1.6 ± 0.1 on *k*_cat_ and 2.0 ± 0.7 on *k*_cat_/*K*_m_ (Figure 3B), which are slightly larger than those derived from competition experiments. We speculate that the observed difference in KIE values between the different experiment types could arise from variations in glycyl radical installation between replicate Michaelis–Menten kinetics assays. Although the magnitude of these KIEs is small, it is reproducible across different days (Figure S8). Together, these data may support a mechanism involving initial HAT from the methylene.

### Incubations in D_2_O

**Figure 4:**
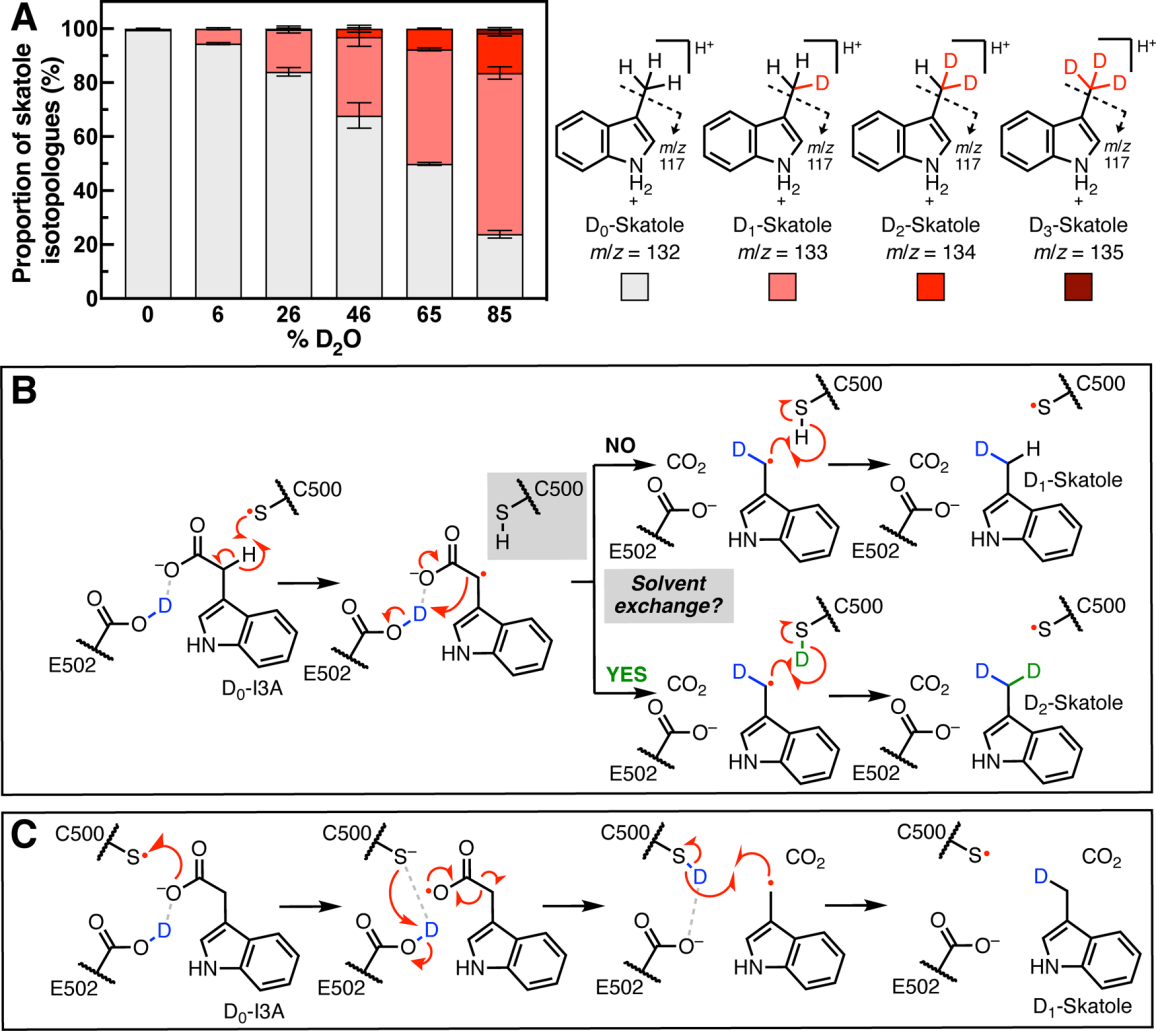
Incubations of D_0_-I3A with IAD in D_2_O lead to production of D_2_-skatole. (A) Incubations of D_0_-I3A with IAD in D_2_O (4.5 h) lead to an extra deuterium being incorporated into the terminal methyl of skatole. Data are mean ± SD (*n* = 3). (B) Proposed mechanism by which deuteria are incorporated into skatole from bulk solvent in a HAT decarboxylation mechanism. (C) Multiple deuteria cannot be incorporated into the skatole product in the Kolbe-type decarboxylation.

To further differentiate between the Kolbe-type and HAT decarboxylation mechanisms, we studied IAD activity in D_2_O, recognizing that these proposed mechanisms invoke intermediates that may participate in solvent exchange to different degrees during catalysis. To examine deuterium incorporation during IAD catalysis, we incubated *Ou* IAD and D_0_-I3A in buffer consisting of increasing ratios of D_2_O to H_2_O and looked for deuterium exchange at the methylene position of I3A and the terminal methyl group of skatole. For each condition, we quantified the four possible skatole isotopologues (*m*/*z* +0, +1, +2, +3) using UPLC–MS/MS (Figure 4A). We observed that in 0% D_2_O, the *m*/*z* +1 peak comes from the natural abundance of ^13^C (theoretical 10.3%, observed 0.5 ± 0.1%). Upon incubation in 26% D_2_O, we observed an increase in the proportion of *m*/*z* +1 (16 ± 1%) corresponding to formation of D_1_-skatole, specifically incorporation of a single deuterium at the terminal methyl group. This result was expected as the net reaction incorporates a proton, mostly likely from a solvent exchangeable residue in IAD or solvent itself. Notably, a significant proportion of D_2_-skatole (15 ± 1%) was produced in 85% D_2_O. The amount of this product exceeded the proportion expected due to natural isotope abundances of D_1_-skatole (6.1%). MS/MS analysis showed that the second deuterium is also localized to the terminal methyl group. In control experiments, we did not observe deuterium incorporation into I3A during the enzymatic reaction. Likewise, incubation of activated *Ou* IAD with skatole did not lead to deuterium incorporation, nor did incubation of either substrate or product in D_2_O without enzyme. In a complimentary experiment, we incubated D_2_-I3A in decreasing ratios of D_2_O to H_2_O and observed loss of deuterium in product (Figure S9A). These results are consistent with those of the analogous experiment using D_2_O and unlabeled substrate.

Of the proposed decarboxylation mechanisms, only a mechanism invoking an initial HAT from the methylene position of I3A could result in incorporation of two deuteria into skatole. Assuming the active site exchanges with bulk solvent, Cys500 could undergo proton-deuterium exchange during catalysis, resulting in transfer of an additional deuterium to the product-based radical (Figures 4B and S9B). The Kolbe-type decarboxylation mechanism (Figures 4C and S9C) cannot account for incorporation of two deuteria, even if there is solvent exchange occurring. We further explored this possibility by analyzing deuterium incorporation into *p*-cresol by HPAD. When HPA and *Cs* HPAD were incubated with D_2_O, only the expected D_1_-*p*-cresol, but not the D_2_-*p*-cresol product, was observed (Figure S10A,B). This result is potentially consistent with the proposed Kolbe decarboxylation mechanism; however, one caveat is that the decarboxylation and re-protonation steps in HPAD could be too fast to allow for active site solvent exchange. Altogether, these results support the use of a HAT mechanism by *Ou* IAD, which is distinct from the mechanism proposed for *Cs* HPAD.

### Computational Modeling

**Figure 5:**
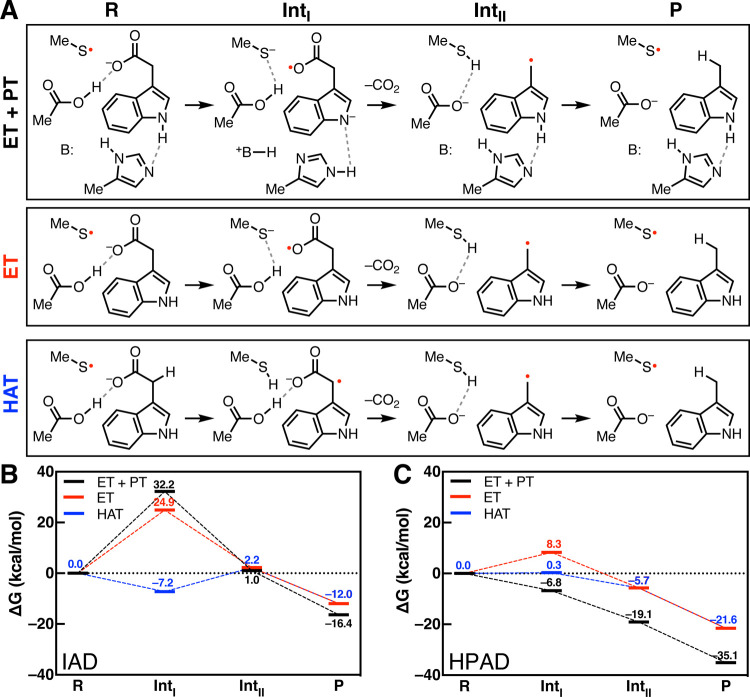
Computational modeling of IAD and HPAD intermediate energies. (A) The three reaction pathway routes modeled for IAD. In the model, backbone atoms are converted to a methyl group. Calculated reaction coordinates for the small models inspired by key active site residues of (B) IAD (Cys500, Glu502, His514) and (C) HPAD (Cys503, Glu505, Glu637).

To better understand the mechanisms of IAD and HPAD, we computationally modeled the stability of potential substrate radical intermediates within systems containing limited active site residues (Cys500, Glu502, His514 for IAD; Cys503, Glu505, Glu637 for HPAD), with the goal of assessing differences in intermediate free energies (Δ*G*) between the two enzymes. We modeled the reaction energetics using domain-based local pair natural orbital coupled-cluster theory (DLPNO-CCSD(T)) with solvent effects computed at the MP2 level of theory using a solvent dielectric of 10 to mimic the protein environment. Entropic contributions were evaluated with hybrid density functional theory, as outlined in the Supporting Information. The active site residue backbones were excluded, and the α-carbons were instead modeled as methyl groups in positions based on the homology model previously generated. Three mechanistic routes were tested: Kolbe-type decarboxylation (ET + PT and ET alone) and HAT for both IAD (Figure 5A) and HPAD (Figure S11).

We found that for IAD, a Kolbe-type decarboxylation reaction generates an endergonic intermediate regardless of whether it involves PT + ET (Δ*G* = 32.2 kcal mol^–1^) or ET alone (Δ*G* = 24.9 kcal mol^–1^), whereas HAT is thermodynamically favored (Δ*G* = −7.2 kcal mol^–1^) (Figure 5B). One caveat is that these values are generated from residue positions of a homology model. However, the general energetic trend should not change with a crystal structure. These calculations estimate a substrate KIE of 6.723 with D_2_-I3A for a HAT mechanism. In comparison, for HPAD, the calculations strongly favor Kolbe-type decarboxylation with PT + ET (Δ*G* = −6.8 kcal mol^–1^) over solely ET (Δ*G* = 8.3 kcal mol^–1^) or HAT (Δ*G* = 0.3 kcal mol^–1^) (Figure 5C). In fact, during modeling of the ET step alone, the residue proposed to deprotonate HPA, Glu637, could not be included because the phenol proton was consistently transferred. These differences in preferred reaction route provide additional support for the proposal that IAD utilizes a HAT mechanism.

## Discussion

The mechanisms of enzymes that catalyze non-oxidative radical decarboxylations are not well understood. The only non-GRE known to perform this chemistry is the FAD-dependent photoenzyme FAP^30^ (Figure S12). Other radical decarboxylases are oxidative, such as the CYP450 OleT,^31^ diiron enzyme UndA,^32^ rSAM MftC,^33^ and non-heme Fe/α-ketoglutarate enzyme IsnB^34^ (Figure S13). Although these oxidative enzymes span multiple protein families and use different cofactors, all proposed mechanisms begin with generation of a carbon-centered radical β to the carboxylate. Comparative study of the three arylacetate GRE decarboxylases IAD, HPAD, and PAD provides a prime opportunity to investigate reaction mechanisms in a class of enzymes that catalyzes related chemical transformations on similar substrates.

The GRE decarboxylases likely have evolved different strategies to achieve the same transformation, depending on the functional groups present in the substrate. For instance, in the Kolbe-type decarboxylation mechanism employed by HPAD, deprotonation of the phenol of HPA by *Cs* HPAD Glu505 greatly stabilizes the carboxylate radical intermediate. Unpublished work has indicated that *Cs* HPAD absolutely requires this Glu for activity.^28^ The corresponding I3A carboxylate radical, conversely, is highly unstable, and *Ou* IAD His514 variants still catalyze skatole decarboxylation. An intermediate with a more comparable free energy is the α-carbon-centered I3A radical. Similarly, the lack of a phenol in PA suggests that PAD initially generates a substrate-based radical via HAT from the benzylic methylene group.^23^ This possibility was explored by assaying the activity of PAD toward α,α-F_2_-PA, which was inferred to bind in the active site as it is a competitive inhibitor. However, no products were generated from this analog, leading the authors to conclude that PAD abstracts an H-atom from the methylene carbon of PA.^23^ It is important to note that the addition of the electron withdrawing fluorine substituents alters the carboxylate oxidation potential, and as a result, a Kolbe-type decarboxylation mechanism cannot be definitively ruled out. This parallels the inconclusive results of incubating *Ou* IAD with α,α-Me_2_-I3A and α-Me-I3A. In contrast, HPAD can decarboxylate *p*-hydroxymandelate, which contains a hydroxyl group at the methylene carbon, to yield *p*-hydroxybenzylalcohol.^2^ One critical gap is that no direct comparison of HPAD reactivity towards α-F- or α-Me-HPA has been conducted, which could provide further evidence for HPAD utilizing a Kolbe-type decarboxylation mechanism.

IAD and HPAD have different substrate scopes, with *C. difficile* HPAD displaying high selectivity for *p*-hydroxyl-containing substrate analogs.^2^ Likewise, analogs where the hydroxyl group is moved to the *ortho* or *meta* positions are not accepted and likely do not bind in the active site, as they do not inhibit HPAD catalysis. The only substrate analog reported to be accepted is 3,4-dihydroxyphenylacetate, which retains the *para* hydroxyl group. Surprisingly, HPAD is unable to decarboxylate the closely related 4-hydroxy-3-methoxyphenylacetate analog,^2^ whereas IAD accepts substrates containing both hydroxyl and methoxy functional groups on the indole ring (Figure S5).

The results of substrate KIE experiments with IAD may support a HAT over a Kolbe-type decarboxylation mechanism. The only other GRE for which substrate D-KIEs have been explored is BSS, which catalyzes the addition of toluene to fumarate. The current mechanistic proposal for BSS invokes HAT from the methyl group of toluene followed by addition of the resulting toluyl radical intermediate into the double bond of fumarate (Figure S14A). This HAT mechanism is largely accepted due to the KIEs observed with perdeuterated D_8_-toluene but not 2,3-D_2_-fumarate.^35,36^ Different KIEs were measured for BSS homologs from different strains, as *Thauera aromatica* strain K172 BSS exhibited a KIE of 4.0 on *V*_max_,^35^ while the *T. aromatica* strain T BSS exhibited a KIE of 1.7 ± 0.2 on *V*_max_ and a KIE of 2.9 ± 0.1 on *V*_max_/*K*_m_.^36^ The authors reasoned that because the KIE on *V*_max_ is significantly smaller than on *V*_max_/*K*_m_, the HAT step from toluene is likely kinetically significant but not fully rate-determining.^36^ In the case of *Ou* IAD, The KIEs on *k*_cat_ and *k*_cat_/*K*_m_ are within error of each other, suggesting that the initial HAT is rate-limiting. The observation of IAD substrate KIEs with D_2_-I3A and D_7_-I3A (1.1–1.6) suggests a HAT mechanism may also be operant and is supported by the computationally predicted KIE (6.723). Although the experimental KIE values are small, they are consistent across different assays and multiple replicates. The difference between the computational and experimental KIE corresponds to an energy barrier of ∼1 kcal mol^–1^, which is within the error range of the calculations and similar to that previously reported for BSS.^37^

Replacing the α-hydrogens with deuterium is not expected to impact catalysis rates in Kolbe-type decarboxylation reactions. For instance, the photocatalytic carbon exchange of the terminal carboxylate of PA with [^13^C]CO_2_ is believed to go through a Kolbe-type decarboxylation mechanism. In these cases, no substrate KIE was detected in competition assays with PA labeled at the methylene position with deuteria.^38^ As a substrate KIE with IAD is observed, a HAT mechanism is likely operant.

Finally, the results of the solvent deuterium exchange experiments with *Ou* IAD provide additional strong support for a HAT mechanism and suggest that that active site Cys is solvent exchangeable. Only a HAT-dependent mechanism allows for two deuteria to be incorporated into product in D_2_O, whereas a Kolbe-type decarboxylation mechanism only results in one deuterium being incorporated. There is precedence for deuterium exchange of GRE active site residues during catalysis. Incubation of D_8_-benzylsuccinate and BSS in H_2_O revealed production of a significant amount of D_7_-toluene in addition to the expected D_8_-toluene.^39^ The authors suggest there is deuterium exchange with a proton on the protein or from bulk solvent, and that the residue most likely involved is the catalytic Cys492 (Figure S14B). Interestingly, this exchange is not detectable in the 250-fold faster forward reaction.^39^

To the best of our knowledge, we are unaware of any precedence for generation of an α-carbon radical facilitating enzymatic decarboxylation beyond the previously proposed mechanism of PAD.^23^ In instances where an α-carbon radical intermediate is generated, recombination or addition to a double bond typically occurs. For example, the CYP450 OleT can generate a radical at both the α- and β-positions of fatty acids. However, while the β-radical can lead to products of both decarboxylation and hydroxyl group rebound, the α-radical results in only hydroxyl group rebound.^31^ Conversely, in organometallic reaction mechanisms, α-oxidation of carboxylic acid substrates has been shown to promote decarboxylation, especially under redox-active conditions of transition-metal or photoredox catalysis.^40^

More broadly, gaining a mechanistic understanding of GRE decarboxylases can inform efforts to manipulate their activity in microbial communities. HPAD and IAD produce *p*-cresol and skatole, respectively, in mammalian gut microbiomes. As both products are associated with host disease, mitigating arylacetate decarboxylation could be a potential therapeutic strategy. Although preliminary inhibitors have been identified for both HPAD^2^ and skatole production in complex fecal samples,^41^ insight into the specific decarboxylations mechanisms can guide the rational design of mechanism-based inhibitors for GRE decarboxylases.

## Conclusions

Microbial non-oxidative radical decarboxylation of arylacetates is a physiologically important reaction that is not well understood mechanistically. Although work with HPAD and PAD had begun to suggest divergent mechanisms amongst the GRE decarboxylases, studies of *Ou* IAD provided an opportunity to fill critical gaps in our understanding of this enzyme class. Our discovery that *Ou* IAD likely employs an initial HAT followed by 2-e^–^ decarboxylation to form skatole rather than a Kolbe-type decarboxylation mechanism highlights how radical enzymes have evolved multiple strategies to catalyze difficult decarboxylation reactions and provides insights that may help develop better mechanism-based inhibitors.

## Supporting Information Available

Full description of materials, detailed procedures for plasmid construction, protein purification, I3A and skatole analog syntheses and characterization, biochemical assays, metabolite analysis, and computational modeling, Tables S1–S9, Figures S1–S14 (PDF)
