# Supplementary material for: Mechanistic Studies of a Skatole-Forming Glycyl Radical Enzyme Suggest Reaction Initiation via Hydrogen Atom Transfer
Source: J Am Chem Soc. 2022 Jun 15;144(25):11110–9. doi: 10.1021/jacs.1c13580 (PMC9248008; doi:10.1021/jacs.1c13580)
Supplement: Supplementary file 1 — ja1c13580_si_001.pdf [file ja1c13580_si_001.pdf]

Supporting Information

Mechanistic Studies of a Skatole-Forming Glycyl Radical Enzyme Suggest Reaction Initiation via Hydrogen Atom Transfer

Beverly Fu,† Azadeh Nazemi,# Benjamin J. Levin,† Zhongyue Yang,# Heather J. Kulik,# and Emily P. Balskus*,†,‡

†Department of Chemistry and Chemical Biology Harvard University, Cambridge, Massachusetts 02138, United States

‡Howard Hughes Medical Institute, Harvard University, Cambridge, Massachusetts, 02138, United States

#Department of Chemical Engineering, Massachusetts Institute of Technology, Cambridge, Massachusetts 02139, United States

Table of Contents

Supplemental figures S3
Figure S1. *In vitro* biochemical experiments verify that *Ou* IAD is a glycyl radical enzyme (GRE) S3
Figure S2. Measuring changes in *Ou* IAD activity over time S4
Figure S3. Comparison of the active sites of the GRE decarboxylases S5
Figure S4. *Ou* IAD homology models constructed using different software prediction software S6
Figure S5. Quantification of substrate analog consumption by *Ou* IAD........ S7
Figure S6. α-Me-I3A and α,α-Me_2-I3A are partial competitive inhibitors of *Ou* IAD S8
Figure S7. KIEs with D_7-I3A indicate the exhibited KIE is due to the two α-deuteria........ S9
Figure S8. Additional replicates of KIE experiments performed on independent days........ S10
Figure S9. Proposed *Ou* IAD decarboxylation mechanism and solvent exchange with D_2-I3A........ S11
Figure S10. Only a single deuterium is incorporated into *p*-cresol by HPAD in D_2O........ S12
Figure S11. The reaction pathway routes modeled for HPAD........ S13
Figure S12. Proposed mechanisms of non-oxidative radical decarboxylations........ S14
Figure S13. Proposed mechanisms of oxidative radical decarboxylations........ S15
Figure S14. Mechanistic studies with benzylsuccinate synthase (BSS)........ S17
Table S1. Kinetic parameters of biochemically characterized GREs........ S18
General materials and methods........ S19
Comparative Genomics S19
Bacterial strains S19
Plasmid construction........ S20
Table S2. Base *E. coli* strains (A), plasmids (B), and oligonucleotides (C) used. S21
Expression and purification of heterologously expressed enzymes S22
Glycyl radical quantification by electron paramagnetic resonance (EPR) spectroscopy S23
Iron and sulfide quantification and UV-vis assays S24
Generation of multiple sequence alignment, homology models, and substrate docked models S24
UPLC–MS/MS assays for detecting end-point SAM cleavage products........ S24
UPLC–MS/MS assays for detecting I3A, skatole, and I3A analogs........ S25
Table S3. UPLC–MS/MS analysis of standards used for assays S25
HPLC assay for detecting I3A, skatole, and I3A analogs S26
Table S4. HPLC analysis of standards used for assays S26
UPLC–MS/MS assay for WT and H514A IAD Michaelis–Menten kinetics S26
UPLC–MS/MS for isotope enrichment assays to determine kinetic isotope effect (KIE) S27
UPLC–MS/MS assay for IAD incubations in D_2O S27
UHPLC–MS assay for HPAD incubations in D_2O S27
Computational details S28
Table S5. Thermodynamic corrections to the electronic energy (E_{el}) in kcal mol^{-1} for IAD........ S28
Table S6. Relative gas-phase DLPNO-CCSD(T)/CBS electronic energies for IAD........ S29
Table S7. Thermodynamic corrections to the electronic energy (E_{el}) in kcal mol^{-1} for HPAD........ S29
Table S8. Relative gas-phase energies relevant to the HPAD system S30
Table S9. Reaction free energies and free energy barriers comparing I3A and α-I3A reactivity........ S30
Synthesis of α-methyl-indole-3-acetic acid and D_3-skatole........ S30
Supplemental references S39

Supplemental figures

Figure S1. In vitro biochemical experiments verify that *Ou* IAD is a glycyl radical enzyme (GRE). (A) SDS-PAGE of purified proteins (2 μg per well). All proteins are His_6-tagged on the N-terminus. Precision Plus Protein All Blue Standards (BioRad) (lane 1), IAD-wild-type (WT) (lane 2), IAD-G853A (lane 3), IAD-C500S (lane 4), IAD-H514A (lane 5), IAD-H514E (lane 6), IAD-E502Q (lane 7), IAD-R226E (lane 8), IAD-R226M (lane 9), IAD-R226K (lane 10), IAD-L616E (lane 11), IAD-F401A (lane 12), IAD-W392F (lane 13), IAD-W392A (lane 14), IAD-AE (lane 15). (B) UV-vis spectrum of *Ou* IAD-AE (± sodium dithionite [NaDT]). (C) Electron paramagnetic resonance (EPR) spectra of WT IAD shows signal characteristic of a glycyl radical. (D) End-point UPLC-MS/MS skatole detection assay using size exclusion chromatography fractions of monomeric or dimeric *Ou* IAD (n =1). (E) End-point UPLC–MS/MS skatole detection and SAM cleavage assays. Full reactions are run with all assay components and either 5 μM IAD and IAD-AE or 50 μM IAD and IAD-AE. Data are mean ± SD (n = 3).

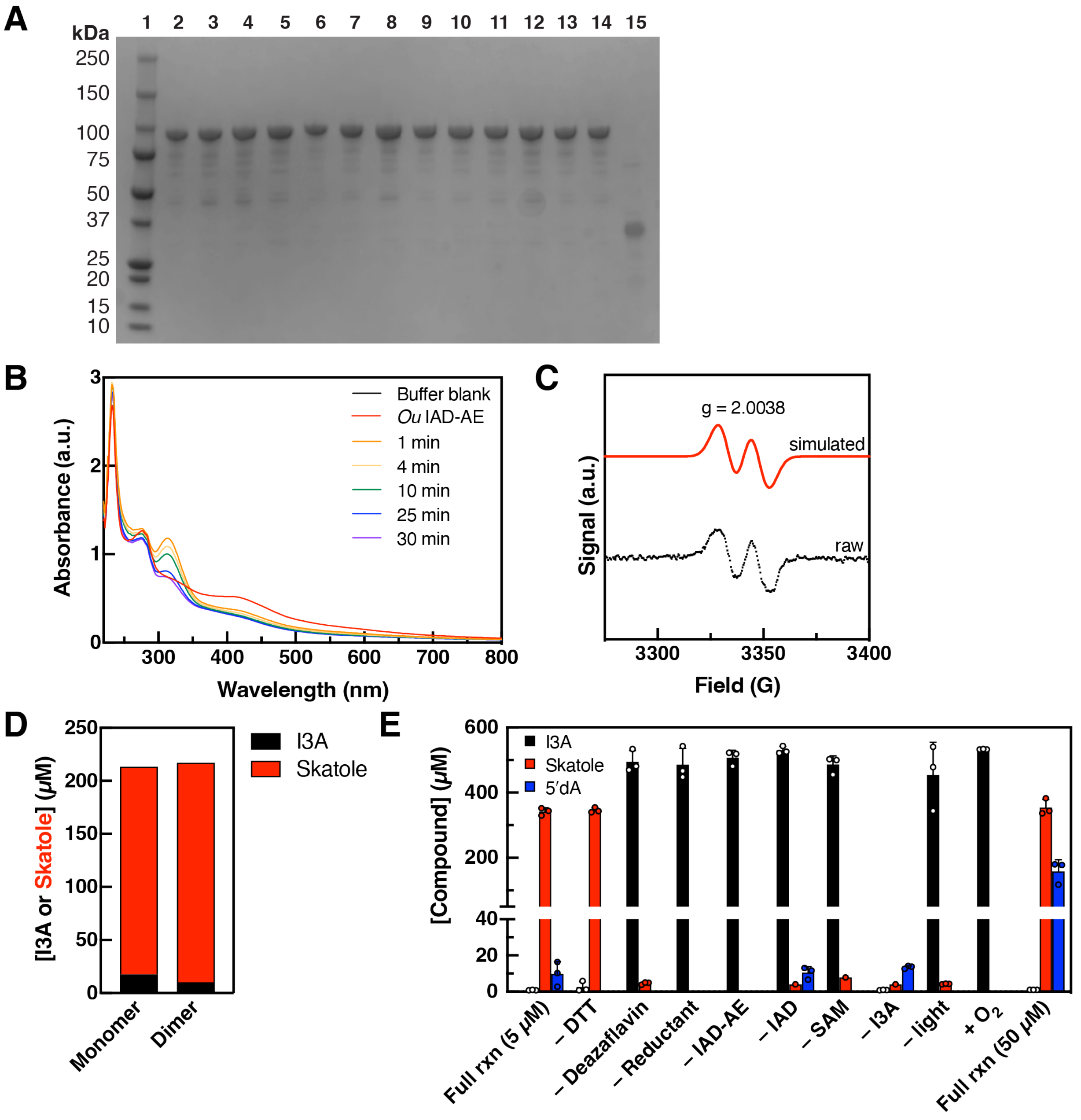

Figure S2. Measuring changes in *Ou* IAD activity over time. (A) Michaelis–Menten kinetics of wild-type (WT) *Ou* IAD with v_0 determined solely at the 10 min time point. Data are the mean ± SE (n = 3) as derived from nonlinear curve fitting to the Michaelis–Menten equation. (B) Michaelis–Menten kinetics of *Ou* IAD H514A point mutant. Data are the mean ± SE (n = 3) as derived from nonlinear curve fitting to the Michaelis–Menten equation.

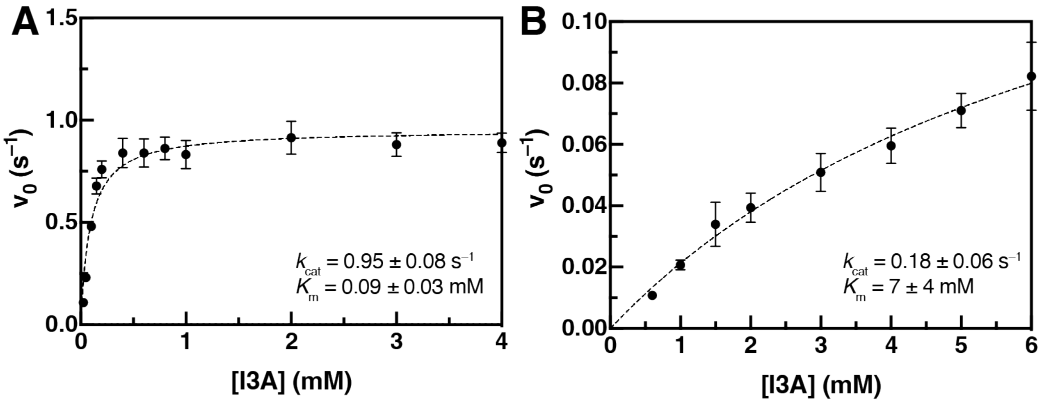

Figure S3. Comparison of the active sites of the GRE decarboxylases. (A) Multiple sequence alignment of GRE decarboxylases. (B) *C. scatologenes* HPAD with 4HP bound in the active site (PDB ID: 2YAJ). Bond distances are highlighted with dashed lines. (C) SWISS-MODEL homology model of PAD (pale green) generated using the structure of *Roseburia inulinivorans* 1,2-propanediol dehydratase (PDB ID: 5I2A) as a template. (D) SWISS-MODEL homology model of *Ou* IAD (pale cyan) generated using the structure of *C. scatologenes* HPAD (2YAJ) as a template.

A

```
OuIAD  GRWIPAY  238    LALGVSPG  346    NSFMNLAV  406
OsIAD  GRWIPAY  231    LALGVSPG  346    NSFMNLAV  406
CsHPAD GREVINY  228    PQSGLSIG  349    NTFNNLSL  410
CdHPAD GREVINY  232    AISGMSIG  353    NTFNTLSI  414
PAD    QIACVKS  210    MTVHHAPM  331    RIAQQLTL  391
OsAAD  WNVPDCA  180    RTYGMGMG  296    SHFHNVVL  357

OuIAD  SGCVENG----------------LAGTGHPIAHP--AFY  518    TLLDSGT  620    RVAGFSAYW  857
OsIAD  SGCVENG----------------LAGTGHPIAHP--AFY  518    TLLDSGT  620    RVAGFSAYW  857
CsHPAD GGCLESAPGCFLPLEYNGKVTMIPGGASPTCGTGVHFI  538    NFESCGT  641    RVAGFTQYW  877
CdHPAD GGCLETSPGCWKQLTLNGKTYSIAGGAGQSAGSGVHFI  542    NVETCGT  645    RVAGFTQYW  881
PAD    AGCVQSIIG------------------GKTDGTWEARF  499    GVCLAGS  603    RVAGFSAYF  819
OsAAD  GGCVLHQVP------------------GKQSSVWPIVQ  464    YIVPVGV  570    RVAGYSAYW  786
```

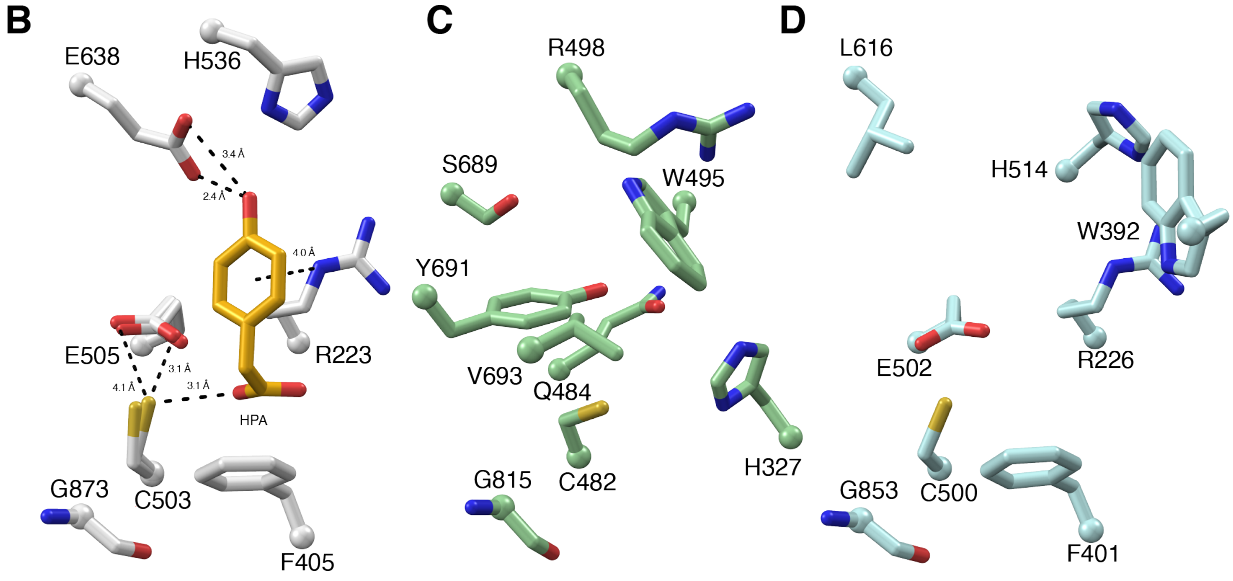

HPAD (2YAJ) **PAD (5I2A)**, 27.4 % aa ID **IAD (2YAJ)**, 33.1 % aa ID

Figure S4. *Ou* IAD homology models constructed using different software prediction software. The (A–C) overall structures, (D–F) active site residues, and (G–I) active sites docked with I3A differ slightly based the program used (SWISS-MODEL[1] [cyan], MOE [purple], or AlphaFold[2, 3] [pink]). Bond distances are indicated with dashed lines.

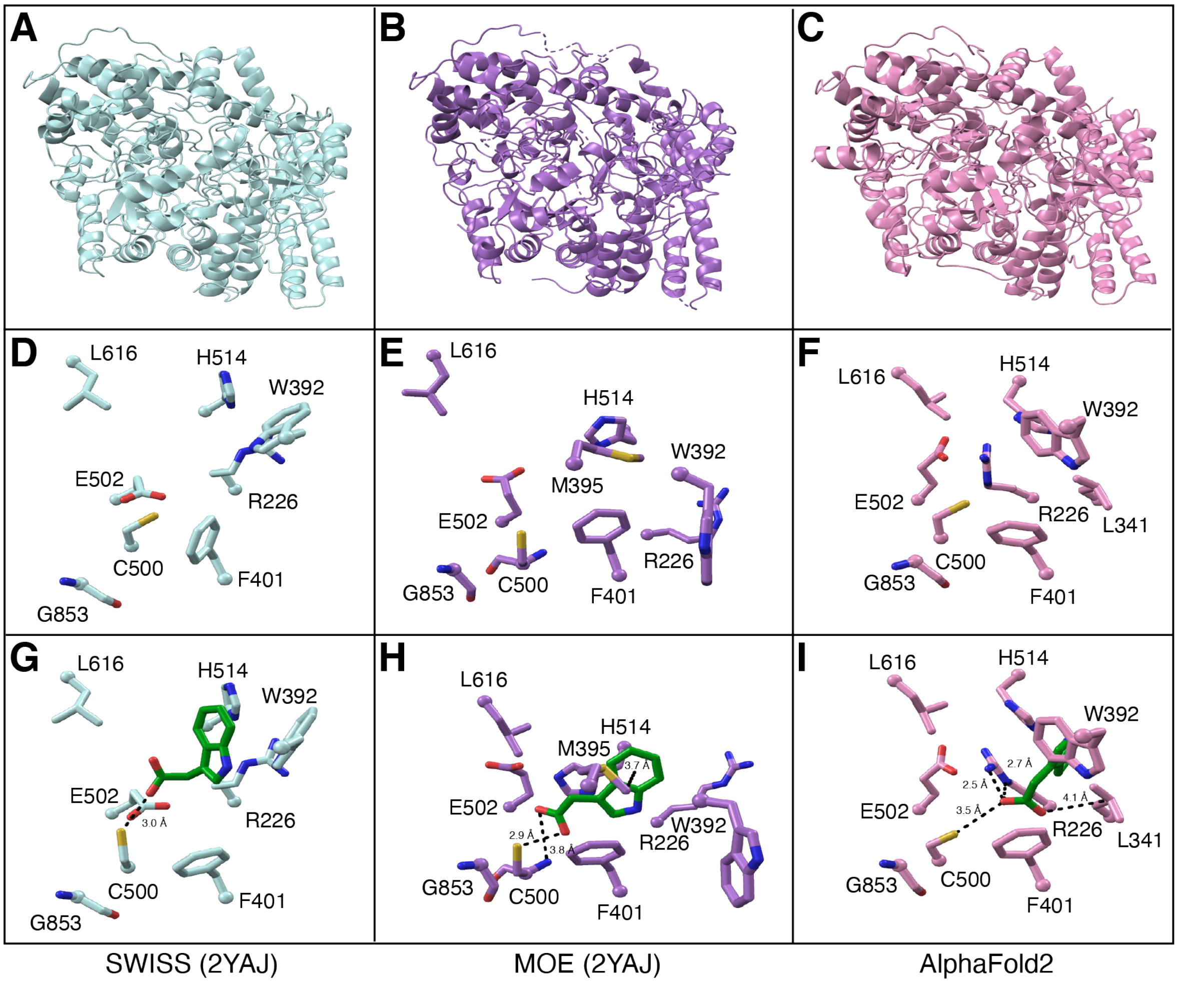

Figure S5. Quantification of substrate analog consumption by *Ou* IAD. Consumption of substrate analog (500 μM) is determined by comparison to a no SAM control and quantified using (A) HPLC or (B) UPLC–MS/MS. Data are mean ± SD (n = 3). (C) Structures and percent conversions. ND, not detected.

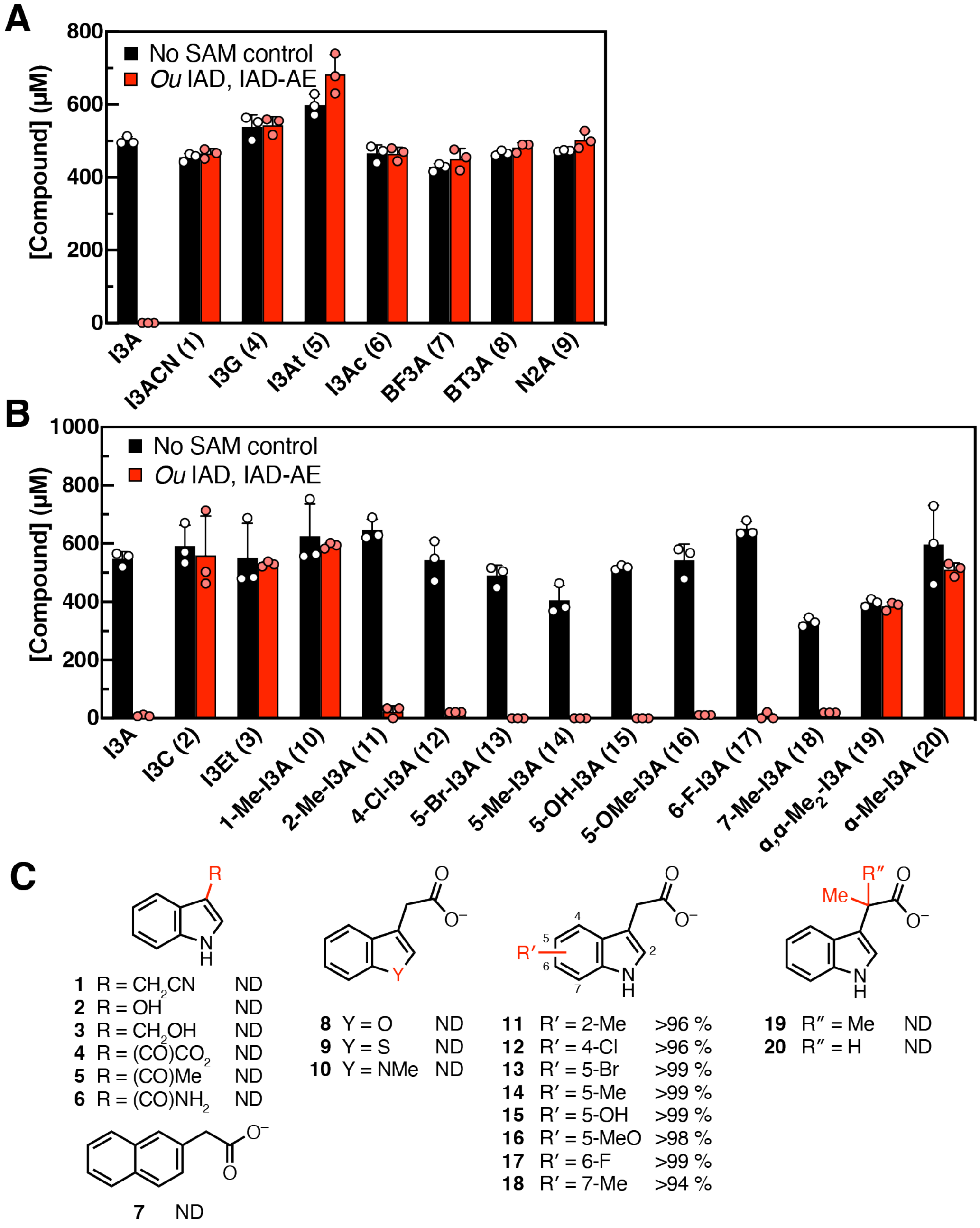

Figure S6. α-Me-I3A and α,α-Me2-I3A are partial competitive inhibitors of *Ou* IAD. Michaelis–Menten plots of (A) benzofuran-3-acetate (**8**), (B) 1-Me-I3A (**10**), (C) α,α-Me_2-I3A (**19**), and (D) α-Me-I3A (**20**). Data are the mean ± SE (n = 2) as derived from nonlinear curve fitting to the Michaelis–Menten equation.

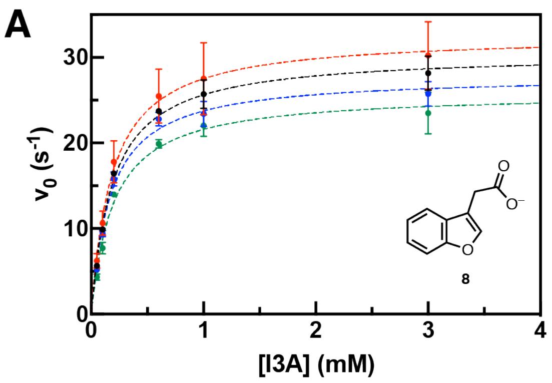

	0 mM	0.5 mM	1 mM	1.5 mM
k_{cat}(s^{-1})	30 ± 2	33 ± 2	28 ± 2	26 ± 2
K_m(mM)	0.19 ± 0.04	0.19 ± 0.08	0.17 ± 0.04	0.20 ± 0.05

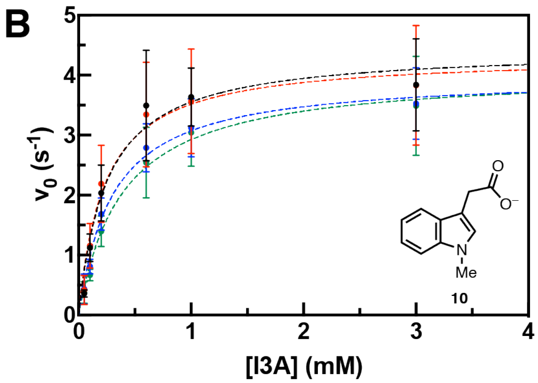

	0 mM	0.5 mM	1 mM	1.5 mM
k_{cat}(s^{-1})	4.4 ± 0.9	4 ± 1	4.0 ± 0.6	4.1 ± 0.8
K_m(mM)	0.3 ± 0.2	0.2 ± 0.2	0.3 ± 0.1	0.4 ± 0.3

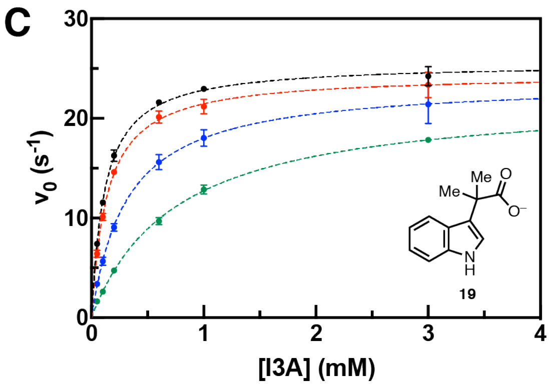

	0 mM	0.5 mM	1 mM	1.5 mM
k_{cat}(s^{-1})	25.6 ± 0.6	24.4 ± 0.8	24 ± 1	22.0 ± 0.8
K_m(mM)	0.12 ± 0.01	0.14 ± 0.03	0.32 ± 0.06	0.75 ± 0.07

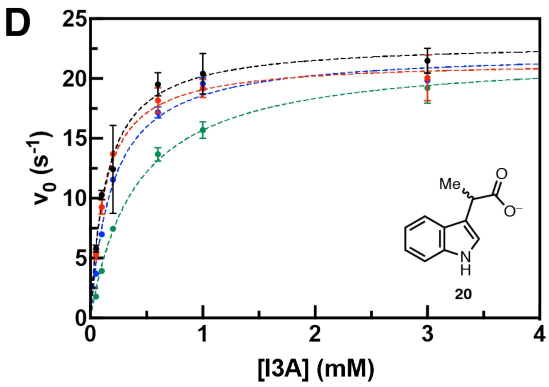

	0 mM	0.5 mM	1 mM	1.5 mM
k_{cat}(s^{-1})	23 ± 2	22 ± 2	22 ± 1	22 ± 2
K_m(mM)	0.14 ± 0.07	0.13 ± 0.04	0.20 ± 0.04	0.4 ± 0.1

Figure S7. KIEs with D_7-I3A indicate the exhibited KIE is due to the two α-deuteria. (A) Substrate enrichment curves show that D_0-I3A is consumed preferentially over D_7-I3A. The inset is of the first 60 s. Data are mean ± SD (n = 3). (B) Substrate enrichment curves showing that D_2-I3A is consumed at about the same rate as D_7-I3A. The inset is of the first 60 s. Data are mean ± SD (n = 3).

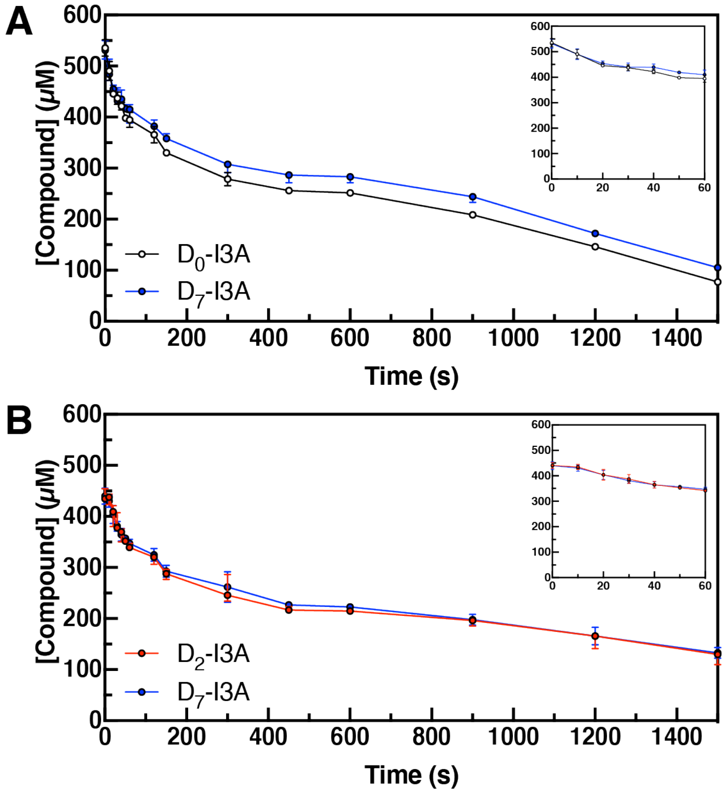

Figure S8. Additional replicates of KIE experiments performed on independent days with (A) D_0 vs D_2-I3A (n=3), (B) D_0 vs D_7-I3A (n=1), and (C) D_2 vs D_7-I3A (n=1).

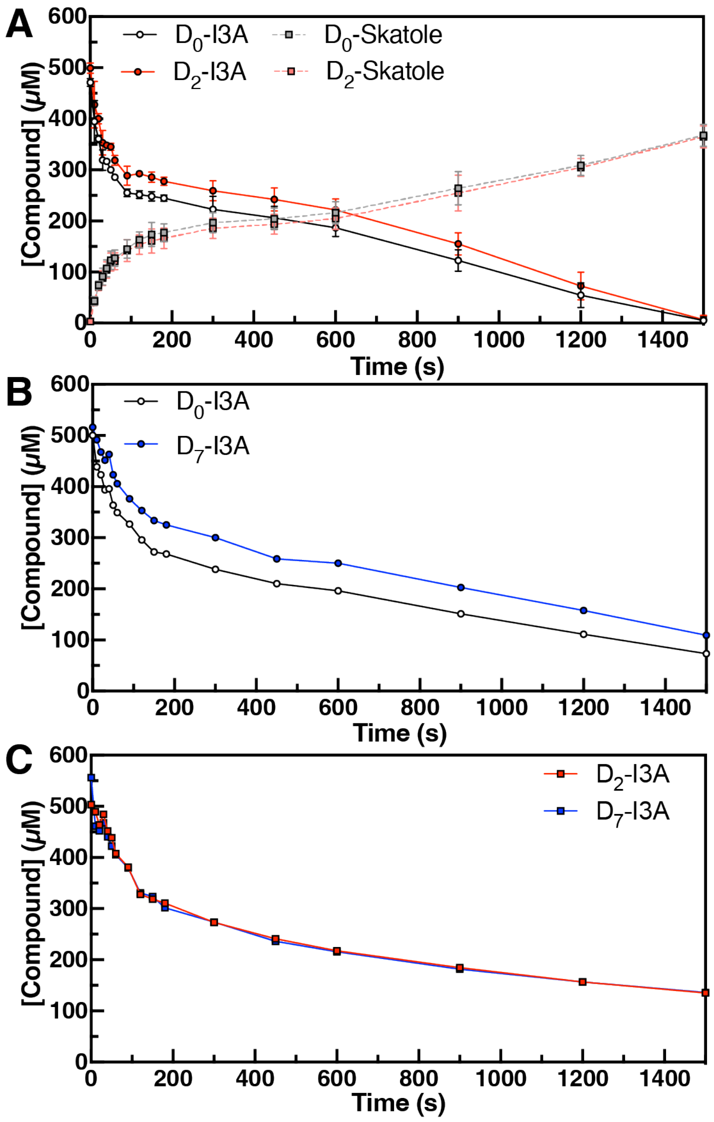

Figure S9. Proposed *Ou* IAD decarboxylation mechanism and solvent exchange with D_2-I3A. (A) Incubations of D_2-I3A in H_2O lead to an extra hydrogen being incorporated into product. Data are mean ± SD (n = 3). (B) Proposed H-atom transfer (HAT) mechanism by which hydrogens are incorporated into skatole from bulk solvent. (C) Multiple hydrogens cannot be incorporated in the Kolbe-type decarboxylation mechanism.

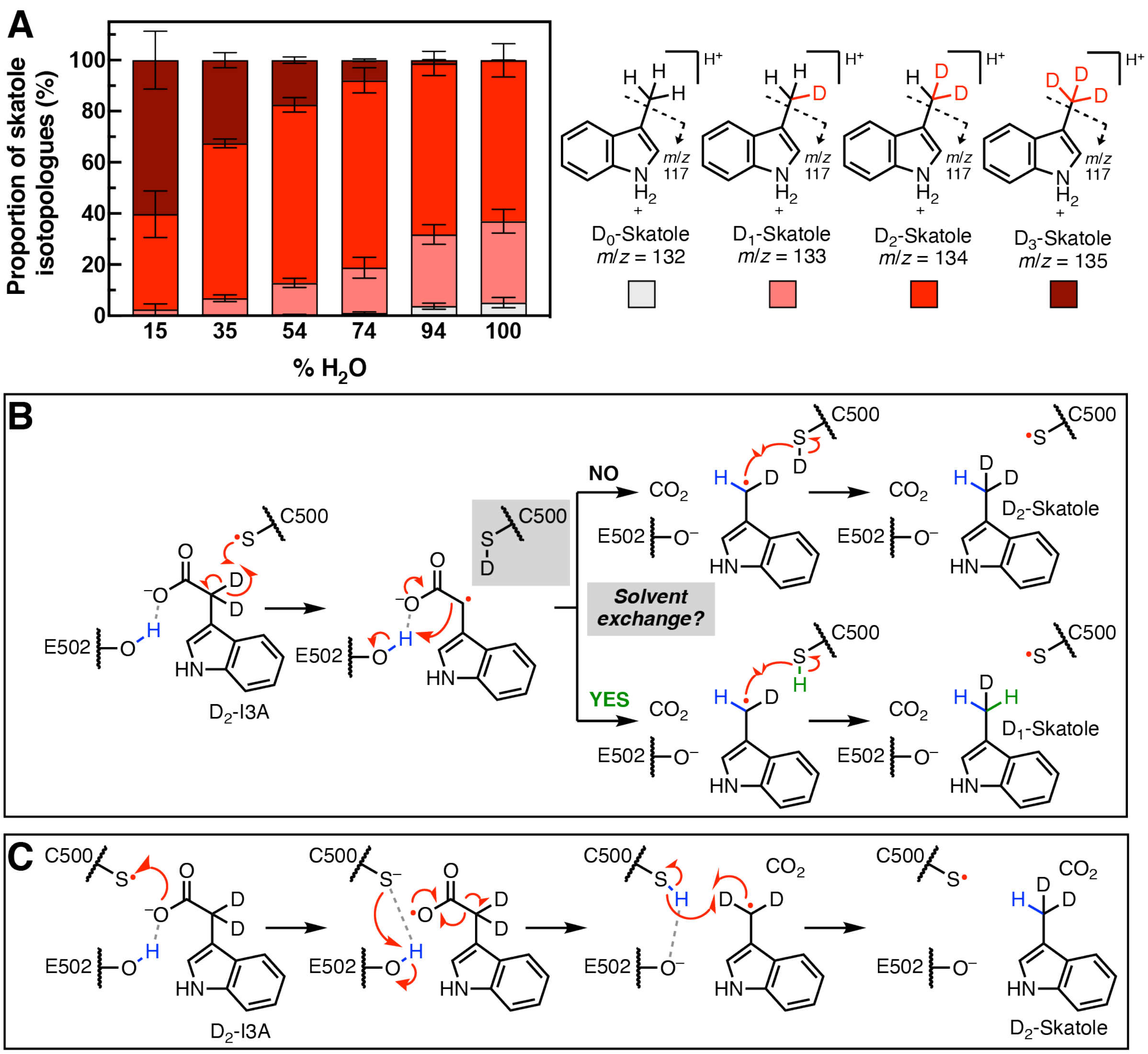

Figure S10. Only a single deuterium is incorporated into *p*-cresol by HPAD in D_2O. End-point (19 h) incubations of HPAD and HPA in (A) H_2O and (B) D_2O. (C) Expected *m*/*z* of *p*-cresol isotopologues.

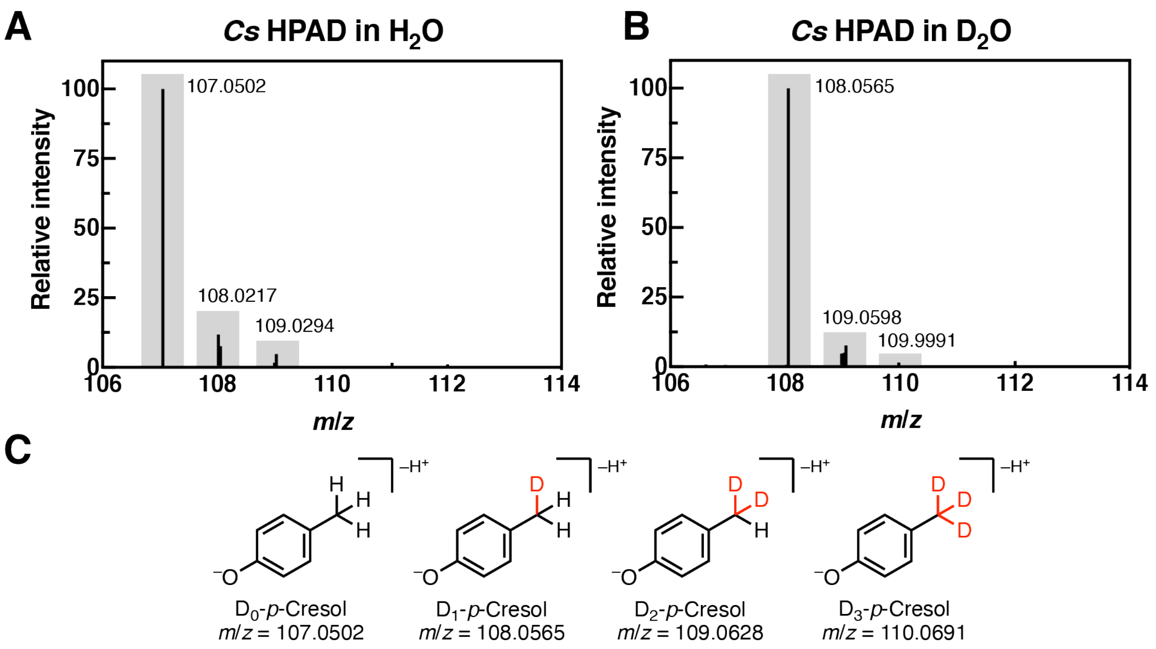

Figure S11. The reaction pathway routes modeled for HPAD. (1) Kolbe-decarboxylation (electron transfer [ET] and proton transfer [PT]), (1) Kolbe-decarboxylation (ET only), (3) H-atom transfer (HAT).

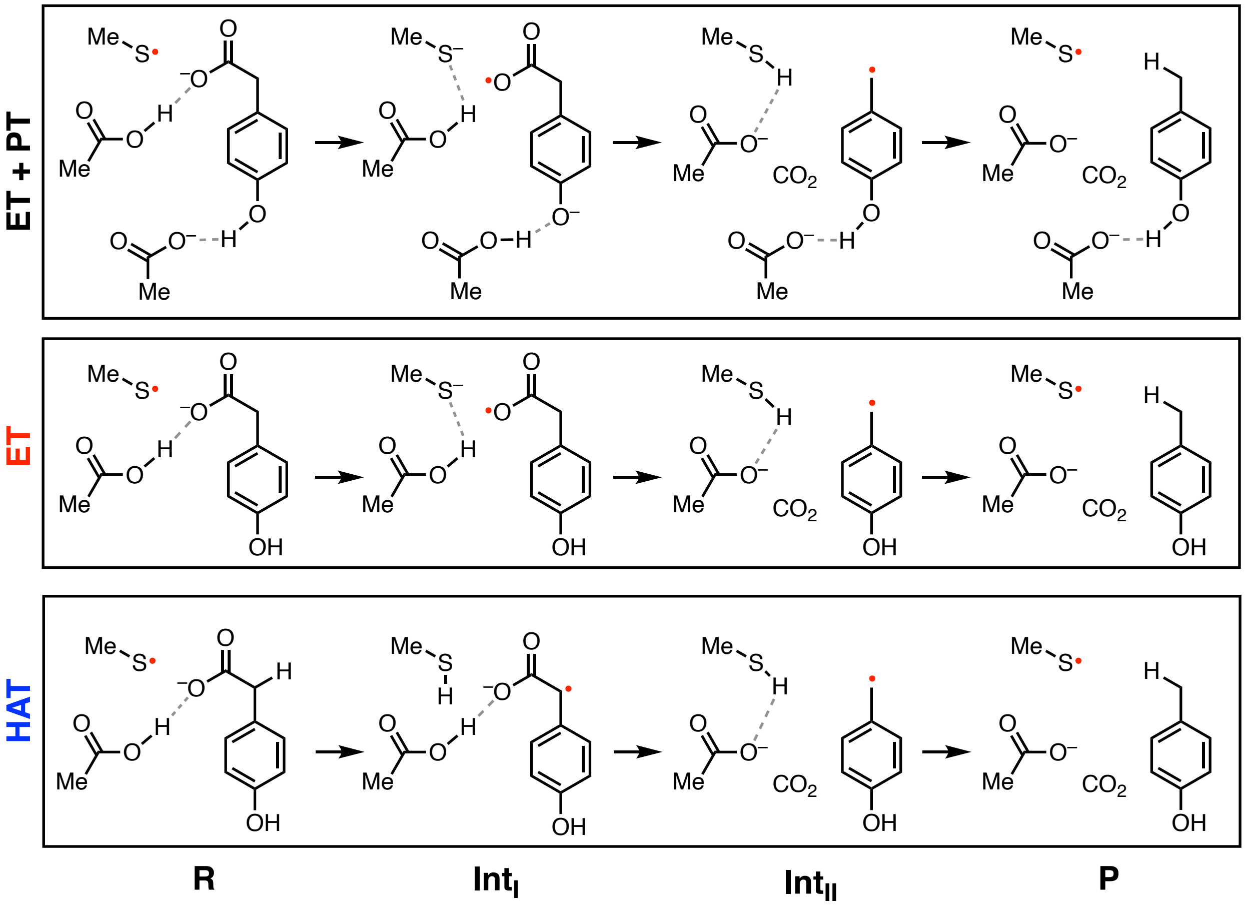

Figure S12. Proposed mechanisms of non-oxidative radical decarboxylations. The FAD-dependent fatty acid photodecarboxylase (FAP) converts $C_{16–18}$ fatty acids into their corresponding (*n*–1)-alkanes.

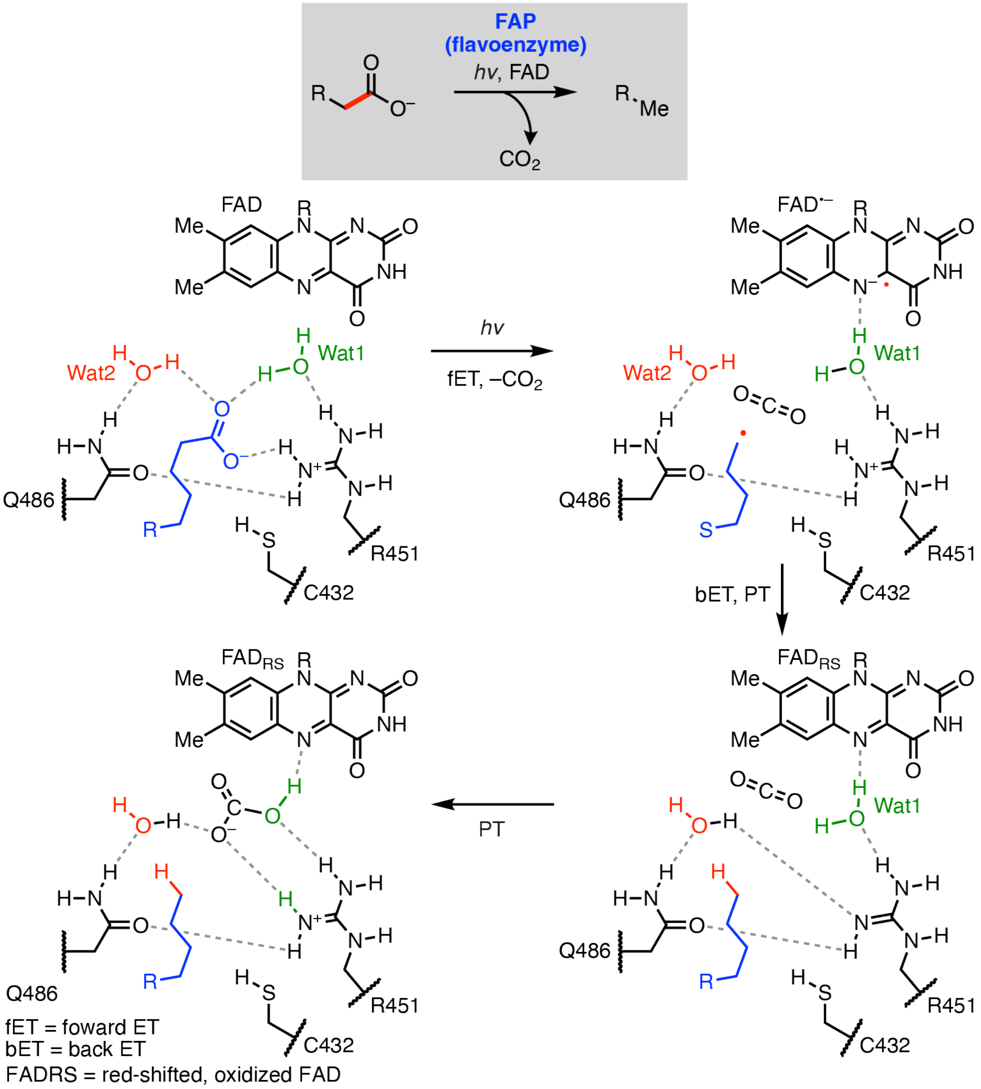

Figure S13. Proposed mechanisms of oxidative radical decarboxylations. Both (A) CYP450 OleT and (B) the diiron enzyme UndA catalyze the conversion of C_x fatty acids into C_{x-1} terminal olefins. (C) The radical SAM enzyme MftC catalyzes C-terminal decarboxylation of a ribosomally-synthesized post-translationally modified peptide (RiPP). (D) The non-heme Fe/α-ketoglutarate enzyme IsnB catalyzes decarboxylation of an isonitrile-containing L-tryptophan precursor.

A

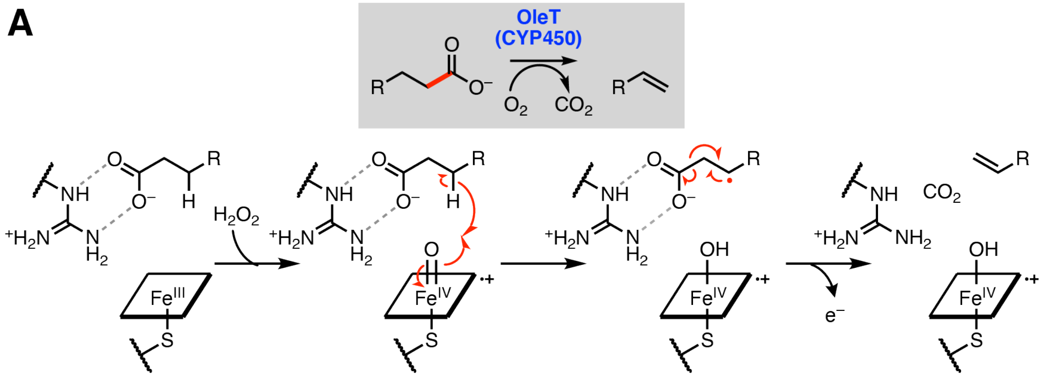

B

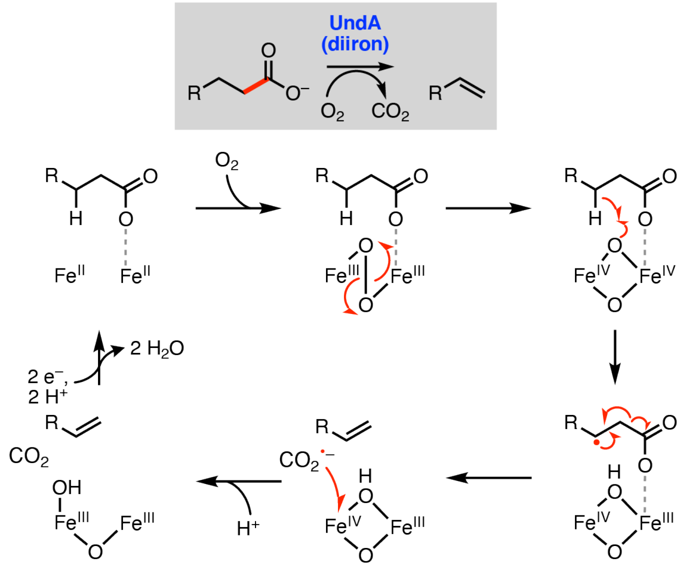

C

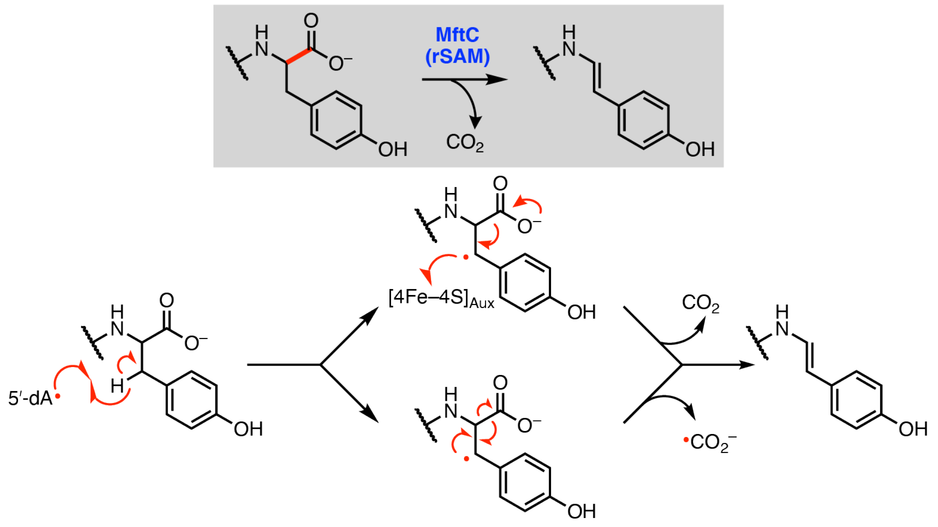

D

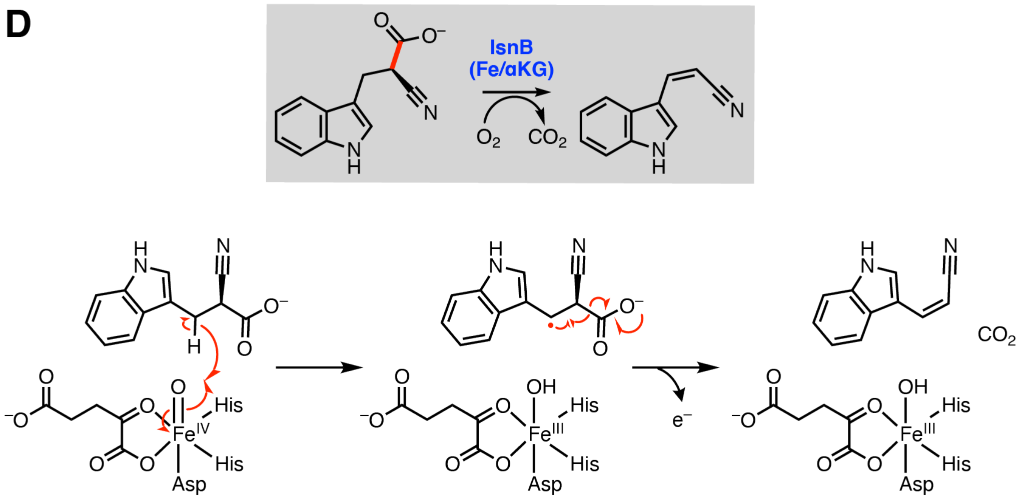

Figure S14. Mechanistic studies with benzylsuccinate synthase (BSS). (A) Current proposed mechanism. (B) Proposed mechanism for hydrogen incorporation into toluene when the BSS reaction is run in reverse.

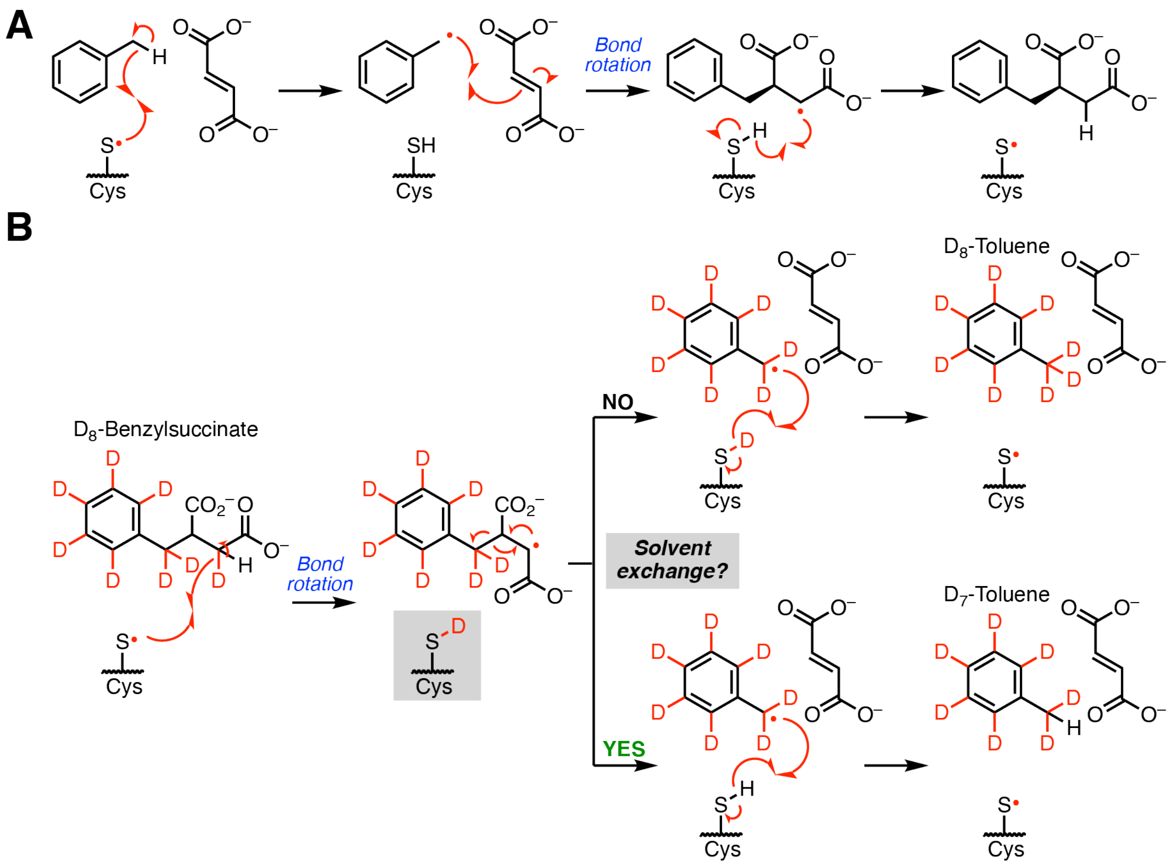

Table S1. Kinetic parameters of biochemically characterized GREs from *Desulfovibrio alaskensis* (*Da*), *Roseburia inulinivorans* (*Ri*), *Clostridioides difficile* (*Cd*), *Escherichia coli* (*Ec*), *Bilophila wadsworthia* (*Bw*), *Klebsiella oxytoca* (*Ko*), *Desulfovibrio vulgaris* (*Dv*), *Desulfovibrio desulfuricans* (*Dd*), *Clostridium scatologenes* (*Cs*), *Olsenella scatoligenes* (*Os*), and *Olsenella uli* (*Ou*). Enzyme abbreviations are CutC (choline trimethylamine-lyase), PD (1,2-propanediol dehydratase), HypD (*trans*-4-hydroxy-L-proline (Hyp) dehydratase), PFL (pyruvate formate-lyase), HpsG (2(*S*)-dihydroxypropanesulfonate [DHPS] sulfolyase), HpfG (DHPS-dehydratase), IslA / IseG (isethionate sulfite-lyase), HPAD (hydroxyphenylacetate decarboxylase), PAD (phenylacetate decarboxylase), IAD (indoleacetate decarboxylase). ND, not determined.

**Enzyme**	**%Active monomer**	**%ID to *Ou* IAD**	**k_{cat} (s^{-1})**	***Glycyl radical normalized* k_{cat} (s^{-1})**	**K_m (mM)**	**k_{cat}/K_m (s^{-1} M^{-1}) with normalized k_{cat}**	**Reference**
Da CutC	20.8	26.2	157 ± 2	755 ± 11	0.13 ± 0.01	$(5.8 \pm 0.5) \times 10^6$	4
Ri PD	18 ± 1	28.1	270 ± 20	1500 ± 100	7.8 ± 0.6	$(1.9 \pm 0.2) \times 10^5$	5
Cd HypD	51 ± 1	26.5	23.0 ± 0.7	45 ± 1	1.2 ± 0.1	$(3.8 \pm 0.3) \times 10^4$	5
Ec PFL	ND	18.7	1100	ND	2.05 ± 0.0068	$(5.37 \pm 0.01) \times 10^5$	6
Bw HpsG	0.02 ± 0.01	24.4	0.005 ± 0.003	26 ± 1	13 ± 2	$(2.0 \pm 0.3) \times 10^3$	7
Ko HpfG	0.015 ± 0.005	24.5	0.020 ± 0.006	130 ± 4	5.0 ± 0.6	$(2.6 \pm 0.3) \times 10^4$	7
Bw IslA	20.8 ± 0.5	24.6	2.0 ± 0.1	9.5 ± 0.6	8 ± 2	$(1.2 \pm 0.31) \times 10^3$	8
	13 ± 0.1		1.82 ± 0.05	14 ± 0.4	8.1 ± 0.8	$(1.7 \pm 0.2) \times 10^3$	9
Dv IseG	20	25.8	18.3 ± 0.7	91.6 ± 3.3	44.8 ± 3.5	$(1.36 \pm 0.05) \times 10^5$	10
Dd IslA	38 ± 3	24.9	157 ± 2	755 ± 11	0.13 ± 0.01	$(5.8 \pm 0.5) \times 10^6$	9
Cs HPAD	9 ± 1	33.1	12.15	135	0.358 ± 0.02	$(3.8 \pm 0.2) \times 10^5$	11
Cd HPAD	9 ± 1	33.7	9.9	110	0.649 ± 0.09	$(1.7 \pm 0.2) \times 10^5$	11
			ND	ND	2.8	ND	12
PAD	ND	26.6	7.5×10^{-7}	ND	2.54 ± 0.38	ND	13
Os AAD	27	25.7	5.9 ± 0.3	21.9 ± 1.1	0.30 ± 0.06	$(7 \pm 2) \times 10^4$	14
Os IAD	14.5	89.3	0.29 ± 0.01	2.0 ± 0.1	0.37 ± 0.06	$(5.41 \pm 0.9) \times 10^3$	15
Ou IAD	19 ± 2	–	2.2 ± 0.2	11.8 ± 0.4	0.10 ± 0.02	$(1.18 \pm 0.2) \times 10^5$	This study

General materials and methods

All chemicals and solvents were purchased from Sigma-Aldrich, except where otherwise noted. α,α-D_2-Indole-3-acetic acid (D_2-I3A) and 5-hydroxyindole-3-acetic acid (5-OH-I3A) were purchased from Santa Cruz Biotechnology, Inc. α,α-D_2-Indole-2,4,5,6,7-D_5-3-acetic acid (D_7-I3A) was purchased from Cambridge Isotope Laboratories. Indole-3-acetone (I3At) was purchased from Life Chemicals Inc. Indole-3-glyoxylic acid (I3G), 5-methylindole-3-acetic acid (5-Me-I3A), and 7-methylindole-3-acetic acid (7-Me-I3A) were purchased from Chem-Impex International. Benzofuran-3-acetic acid (BF3A) was purchased from Maybridge Chemical Company Limited. Benzothiophene-3-acetic acid (BT3A) was purchased from Alfa Aesar. 1-Methylindole-3-acetic acid (1-Me-I3A), 5-bromoindole-3-acetic acid (5-Br-I3A), and 5-methoxyindole-3-acetic acid (5-OMe-I3A) were purchased from Combi-Blocks. 2-Methylindole-3-acetic acid (2-Me-I3A) was purchased from Pharmacia & Upjohn Company. 4-Chloroindole-3-acetic acid (4-Cl-I3A) was purchased from Toronto Research Chemicals. 6-Fluoroindole-3-acetic acid (6-F-I3A) was purchased from Oakwood Chemical. α,α-Dimethyl-indole-3-acetic acid (α,α-Me_2-I3A) was purchased from Ambeed, Inc.

Olsenella uli DSM 7084 genomic DNA (gDNA) was purchased from DSMZ. Luria-Bertani Lennox (LB) medium was purchased from Alfa Aesar. DNA sequencing results were analyzed with Benchling (Benchling, Inc.). Multiple sequence alignments were analyzed with Geneious Pro 11.0.4 (Biomatters)[16] or Clustal Omega.[17] All restriction enzymes, ligases, polymerases, PCR mixes, and Gibson Assembly mixes were obtained from New England Biolabs (NEB). SDS-PAGE (4–15% Mini-PROTEAN TGX gel, Bio-Rad) was routinely used to visualize fractions from protein purifications following staining (EZBlue Gel Staining Reagent, Sigma-Aldrich). Isopropyl β-D-1-thiogalactopyranoside (IPTG) was obtained from Teknova. Ni-NTA resin was obtained from Qiagen. Dithiothreitol (DTT) was purchased from VWR International. *S*-adenosyl-L-methionine (SAM) was purchased from Sigma-Aldrich as a *p*-toluenesulfonate salt. Solvents used for LC–MS were B&J Brand high-purity solvents (Honeywell Burdick & Jackson).

Samples were made anaerobic as follows. Solids were brought into anaerobic chambers (MBraun and Coy Labs) in perforated 1.7 mL microcentrifuge tubes. Protein solutions with volumes greater than 1 mL were made anaerobic on a Schlenk line with 20 cycles of evacuation on vacuum followed by filling with argon or nitrogen. Solutions (<50 mL) were made anaerobic by bubbling argon or nitrogen through the liquid for 20 min. Solutions (≥50 mL) were made anaerobic on a Schlenk line with 3 cycles of evacuation on vacuum followed by filling with argon or nitrogen for 30 min each while stirring.

Comparative Genomics

At the beginning of our work, four *Olsenella* species (*O. scatoligenes* SK9K4^T, *O. uli* DSM 7084^T, *O. profusa* DSM 13989^T, and *O. umbonata* DSM 22620^T) had been sequenced, and it was known that only the former two species could decarboxylate I3A into skatole.[18, 19] BLAST searches identified four GREs encoded in *O. scatoligenes*, five in *O. uli*, and only three in the remaining species. By comparing GREs found in skatole producers versus non-producers, the putative *Ou* IAD gene (89 % amino acid ID [aa ID] to *Os* IAD) was identified. A putative *Ou* IAD-AE enzyme (73.3 % aa ID to *Os* IAD-AE) belonging to the radical SAM enzyme family was co-localized with this GRE. To further corroborate this search, a putative IAD gene was also identified in the genome of *Clostridium scatologenes* ATCC 25775,[11] a known skatole-producer (51 % aa ID to *Ou* IAD, 33 % aa ID to *Cs* HPAD). Because HPAD is the characterized GRE most similar to these putative IADs, we hypothesized we had correctly identified IAD.

Bacterial strains

E. coli Top10 (Invitrogen) was routinely used for DNA construction and either BL21(DE3) (Invitrogen), BL21(DE3) ΔiscR,[9] BL21(DE3) + CodonPlus-RIL (Agilent), or BL21(DE3) ΔiscR + CodonPlus-RIL[9] was used for heterologous production of proteins for biochemical experiments.

To prepare chemically competent cells for plasmid transformations, the appropriate *E. coli* strain was cultured from a single colony in 100 mL LB broth, harvested at OD_{600} of 0.4–0.5, and resuspended in 10 mL of sterile Transformation-Storage Solution (10% w/v PEG 6000, 5% v/v DMSO, 20 mM $MgCl_2$ in LB broth). 100 μL aliquots were flash frozen in liquid nitrogen and stored at –80 °C for later use. Plasmid transformations were performed by adding one or more plasmid (100 ng), 20 μL 5× KCM solution (0.5 M KCl, 0.15 M $CaCl_2$, 0.25 M $MgCl_2$), and 80 μL sterile water to a thawed competent cell aliquot of the appropriate strain. After 10 min incubation on ice, cells were heat shocked at 42 °C for 90 s, diluted to 1 mL with LB broth, and incubated at 37 °C for 1 h before plating with sterile beads on LB agar containing appropriate antibiotics.

Plasmid construction

Plasmid construction was carried out using standard molecular biology techniques. Descriptions of strains and plasmids are listed in **Table S2AB**. PCR amplifications were carried out with Q5 High-Fidelity or Phusion High-Fidelity polymerase, following manufacturer instructions. Primers (Sigma-Aldrich) are listed in **Table S2C**. Typical PCR reactions (total volume 50 μL) contained 10 ng template, 0.6 μM forward primer, 0.6 μM reverse primer, optional 1% DMSO, and either Q5-HF or 2× Phusion-HF Master Mix (25 μL) (NEB). PCR parameters were as follows: Initial denaturation (98 °C for 30 s), 30 cycles of denaturation (98 °C for 10 s), annealing (55 °C for 30 s), extension (72 °C for 90 s), and a final extension (72 °C for 5 min). All PCR amplifications were analyzed by 1% agarose gel electrophoresis with ethidium bromide staining in 1× Tris-acetate-EDTA (TAE) buffer. PCR products and digested DNA were purified using Zymoclean DNA Clean & Concentrator Kit or Gel DNA Recovery Kit (Zymo Research). Constructs were assembled using Gibson Assembly[20] (NEB), following manufacturer instructions. Constructs were verified by sequencing (Eton Bioscience or Genewiz). DNA was purified using E.Z.N.A. Plasmid MiniKit (Omega Bio-tek) and transformed into chemically competent *E. coli* BL21(DE3) cells. Transformed cells were stored at –80 °C as frozen LB/glycerol stocks.

Construction of plasmids for the expression of Ou IAD and Ou IAD-AE. pET28a-OuIAD and pET28a-OuIAD-AE were constructed by amplifying the *Ou* IAD (UniProt ID: E1QXZ2) and *Ou* IAD-AE (UniProt ID: E1QXZ4) genes from *Olsenella uli* DSM 7084 gDNA (DSMZ, Braunschweig, Germany) using primer sets OuIAD_FP & OuIAD_RP and OuIAD-AE_FP & OuIAD-AE_RP, respectively. These fragments were inserted into pET28a digested with NdeI and XhoI using the Gibson Assembly.[20]

Site-directed mutagenesis of Ou IAD. pET28a-OuIAD(G853A) was constructed by amplifying the *Ou* IAD gene in two segments using primer sets pET28a_FP & OuIAD(G853A)_RP and OuIAD(G853A)_FP & pET28a_RP. The two segments were inserted into pET28a digested with NdeI and XhoI using the Gibson Assembly.[20] Other *Ou* IAD mutant constructs (C500S, H514A, H514E, E502Q, R226E, R226M, R226K, L616E, F401A, W392F, W392A) were constructed using a similar strategy.

Construction of plasmids for the expression of Cs HPAD and Cs HPAD-AE. pET28a-CsHPAD_β-γ was synthesized to encode CsHPAD_β (Uniprot ID: Q38HX4) and CsHPAD_γ (Uniprot ID: Q38HX3) under a single T7 promoter (Twist Bioscience). The intergenic region from *C. scatologenes* ATCC 25775 was kept. pET28a-CsHPAD-AE was constructed by amplifying the *Cs* HPAD-AE (UniProt ID: Q38HX2) gene from *C. scatologenes* ATCC 25775 gDNA (DSMZ, Braunschweig, Germany) using primer sets CsHPAD-AE_FP & CsHPAD-AE_RP. These fragments were inserted into pET28a digested with NdeI and XhoI using the Gibson Assembly.[20]

Table S2. Base *E. coli* strains (A), plasmids (B), and oligonucleotides (C) used.

A. E. coli strains

Strain	*Genotype*	*Source*
Top10	F^-*mcr*A Δ(*mrr-hsd*RMS-*mcr*BC) φ80*lac*ZΔM15 Δ*lac*X74 *rec*A1 *ara*D139 Δ(*ara-leu*)7697 *gal*U *gal*K λ^-*rps*L(Str^R) *end*A1 *nup*G	Invitrogen
BL21(DE3)	F^-*omp*T *hsd*S_B (r_B^-, m_B^-) *gal dcm* (DE3)	Invitrogen
BL21(DE3) ΔiscR	F^-*omp*T *hsd*S_B (r_B^-, m_B^-) *gal dcm* (DE3) Δ*iscR*	Dr. Benjamin J. Levin[5]
BL21(DE3), CodonPlus-RIL	F^-*omp*T *hsd*S (r_B^-, m_B^-) *dcm*$^+$ Tet^r *gal* λ(DE3) *endA* Hte [*argU ileY leuW* Cam^r]	Agilent
BL21(DE3) ΔiscR, CodonPlus-RIL	F^-*omp*T *hsd*S (r_B^-, m_B^-) *dcm*$^+$ Tet^r *gal* λ(DE3) *endA* Hte [*argU ileY leuW* Cam^r] Δ*iscR*	Dr. Benjamin J. Levin[5]

B. Plasmids

Plasmid	*Description*	*Source*
pET28a-OuIAD	*His_6-OuIAD* (T7), *lacI*, Km^r, pBR322	This study
pET28a-OuIAD-AE	*His_6-OuIAD-AE* (T7), *lacI*, Km^r, pBR322	This study
pET28a-OuIAD(G853A)	*His_6-OuIAD(G853A)* (T7), *lacI*, Km^r, pBR322	This study
pET28a-OuIAD(C500S)	*His_6-OuIAD(C500S)* (T7), *lacI*, Km^r, pBR322	This study
pET28a-OuIAD(H514A)	*His_6-OuIAD(H514A)* (T7), *lacI*, Km^r, pBR322	This study
pET28a-OuIAD(H514E)	*His_6-OuIAD(H514E)* (T7), *lacI*, Km^r, pBR322	This study
pET28a-OuIAD(E502Q)	*His_6-OuIAD(E502Q)* (T7), *lacI*, Km^r, pBR322	This study
pET28a-OuIAD(R226E)	*His_6-OuIAD(R226E)* (T7), *lacI*, Km^r, pBR322	This study
pET28a-OuIAD(R226M)	*His_6-OuIAD(R226M)* (T7), *lacI*, Km^r, pBR322	This study
pET28a-OuIAD(R226K)	*His_6-OuIAD(R226K)* (T7), *lacI*, Km^r, pBR322	This study
pET28a-OuIAD(L616E)	*His_6-OuIAD(L616E)* (T7), *lacI*, Km^r, pBR322	This study
pET28a-OuIAD(F401A)	*His_6-OuIAD(F401A)* (T7), *lacI*, Km^r, pBR322	This study
pET28a-OuIAD(W392F)	*His_6-OuIAD(W392F)* (T7), *lacI*, Km^r, pBR322	This study
pET28a-OuIAD(W392A)	*His_6-OuIAD(W392A)* (T7), *lacI*, Km^r, pBR322	This study
pET28a-CsHPAD_β-γ	*His_6-CsHPAD*_β-γ (T7), *lacI*, Km^r, pBR322	Twist Bioscience
pET28a-CsHPAD-AE	*His_6-CsHPAD-AE* (T7), *lacI*, Km^r, pBR322	This study

C. Oligonucleotide sequences

Name	*Sequence (5′ to 3′)*
OuIAD_FP	gccgcgcggcagccatatggaggagtctctggttctcg
OuIAD _RP	cagtggtggtggtggtggtgctcgagctagagagcgtcgtactcggtacg
OuIAD-AE_FP	gccgcgcggcagccatatggacggcaagggcaaagagc
OuIAD-AE_RP	cagtggtggtggtggtggtgctcgagtcagttgataacgcactcgatccc
pET28a_FP	cctggtgccgcgcggcagccatatg
pET28a_RP	agtggtggtggtggtggtgctcgag
OuIAD(G853A)_FP	cgtgtcgccgccttctcagcc
OuIAD(G853A)_RP	ggctgagaaggcggcgacacg
OuIAD(C500S)_FP	ctctccggcagcgtcgagaac
OuIAD(C500S)_RP	gttctcgacgctgccggagag
OuIAD(H514A)_FP	cccatcgccgcgccggccttc
OuIAD(H514A)_RP	gaaggccggcgcggcgatggg
OuIAD(H514E)_FP	cccatcgccgaaccggccttc
OuIAD(H514E)_RP	gaaggccggttcggcgatggg
OuIAD(E502Q)_FP	ggctgcgtccagaacggcctc
OuIAD(E502Q)_RP	gaggccgttctggacgcagcc
OuIAD(R226E)_FP	tgtgacggcgaatggattccc
OuIAD(R226E)_RP	gggaatccattcgccgtcaca
OuIAD(R226M)_FP	tgtgacggcatgtggattccc
OuIAD(R226M)_RP	gggaatccacatgccgtcaca
OuIAD(R226K)_FP	tgtgacggcaaatggattccc
OuIAD(R226K)_RP	gggaatccatttgccgtcaca
OuIAD(L616E)_FP	gtcacgctggaagactccggt
OuIAD(L616E)_RP	accggagtcttccagcgtgac
OuIAD(F401A)_FP	tcgaactcggcgatgaacctt
OuIAD(F401A)_RP	aaggttcatcgccgagttcga

OuIAD(W392F)_FP	ccgtccctctttgcggccatg
OuIAD(W392F)_RP	catggccgcaaagagggacgg
OuIAD(W392A)_FP	ccgtccctcgcggcggccatg
OuIAD(W392A)_RP	catggccgccgcgagggacgg
CsHPAD-AE_FP	atcatcatcacagcagcggcctggtgccgcgcggcagccatatgaaggaaaaaggtttaatatttgatatacaaagcttttctgtacatg
CsHPAD-AE_RP	gccggatctcagtggtggtggtggtggtgctcgagttaaaaaggcgtattatcacctatatagcaagctatattattatctaaatataac

Expression and purification of heterologously expressed enzymes

Expression and purification of His$_6$-tagged Ou IAD enzymes. Ou IAD and corresponding mutants were expressed and purified in a similar fashion. Chemically competent *E. coli* BL21(DE3) was transformed with the appropriate pET28a expression vector. An overnight culture of the expression strain was grown in LB containing 50 μg mL^{-1} kanamycin (Km) starting from a colony or glycerol stock. 2 L of LB media in a 2.8 L Erlenmeyer baffled flask with 50 μg mL^{-1} of Km was inoculated with 40 mL of starter culture (2%). The culture was grown at 37 °C at 180 rpm to OD_{600} = 0.5–0.6, at which point cultures were cooled on ice for 20 min, induced with 500 μM IPTG, and grown overnight at 16 °C at 180 rpm. Cell pellets were harvested by centrifugation at 6,730 × g for 10 min at 4 °C, flash frozen in N_2 (*l*), and stored at –80 °C.

All subsequent steps were performed at 4 °C unless otherwise specified. Frozen cell pellets were thawed and resuspended at 5 mL per g cell paste with Buffer A1 (25 mM Tris, 200 mM NaCl, pH 8). Cells were lysed by three passages through a cell disrupter (Avestin EmulsiFlex-C3) at 10,000 psi or by sonicating with a ½ inch horn at 25% amplitude for 4 min (2.5 s on followed by 5 s off) while being kept in an ice bath. Lysate was centrifuged at 20,000 × g for 30 min at 4 °C to separate soluble and insoluble fractions. The soluble lysate was loaded onto a Ni-NTA column (3 mL resuspended resin per 2 L expressed pre-equilibrated with Buffer A1) by gravity flow. The column was washed with 10 column volumes (c.v.) of 90% Buffer A1, 10% Buffer A2 (25 mM Tris, 200 mM NaCl, 250 mM imidazole, pH 8) until no protein was detectable by Bradford Protein Assay (Bio-Rad). Protein was eluted from the column with Buffer A2 in 5 mL fractions. Fractions containing protein, as detected by Bradford, were combined, transferred to a Slide-a-Lyzer™ dialysis cassette with a 20 kDa MWCO (Thermo Scientific), and dialyzed three times against 1 L of Buffer A1. After dialysis, the solution was concentrated using a Ultra-15 Centrifugal Filters centrifugal concentrator with a 30 kDa MWCO membrane (Amicon) to a protein concentration of ~350 μM. Protein concentrations were estimated with a using NanoDrop 2000 UV-Vis Spectrophotometer (Thermo Scientific) using ε_{280nm} calculated by ExPASY ProtParam[21] as follows: *Ou* IAD and all point mutants except W392F and W392A (ε_{280} = 149,660 M^{-1} cm^{-1}); *Ou* IAD(W392F) and *Ou* IAD (W392A) (ε_{280} = 144,160 M^{-1} cm^{-1}). The concentrated protein solution was made anaerobic on a Schlenk line with 20 cycles of evacuation on vacuum and filling with nitrogen and brought into an anaerobic chamber at 4 °C containing 97% N_2 and 3% H_2 (CoyLabs), aliquoted into 0.5 mL cryogenic vials, and placed in 18 × 150 mm Balch-type tubes (Chemglass). The tubes were sealed with butyl stoppers and aluminum seals, frozen in N_2 (*l*), and stored at –80 °C.

Determination of Ou IAD oligomeric state. Anaerobic size-exclusion chromatography was conducted inside an anaerobic chamber at 4 °C containing 97% N_2 and 3% H_2 (CoyLabs) with fast performance liquid chromatography (FPLC) (Bio-Rad BioLogic DuoFlow System equipped with GE Life Sciences DynaLoop90). Degassed *Ou* IAD was loaded onto a HiLoad 26/600 Superdex 200 pg size exclusion column (Cytiva) pre-equilibrated in 25 mM Tris, pH 8, 200 mM NaCl, and 1 mM DTT. The resulting fractions were pooled and concentrated with a 30 kDa MWCO membrane (Amicon).

Expression and purification of His$_6$-tagged Ou IAD-AE, Cs HPAD-AE, and Cs HPAD enzymes. Ou IAD-AE, Cs HPAD-AE, and Cs HPAD were overexpressed and purified based on a reported protocol.[22] Chemically competent *E. coli* BL21(DE3) (CodonPlus-RIL) ΔiscR was transformed with the appropriate pET28a expression vector. An overnight culture of the appropriate strain was grown in LB media containing 50 μg mL^{-1} Km and 50 μg mL^{-1} chloramphenicol (Cm), starting from a colony or glycerol stock. 2 L of LB media supplemented with 20 mM $MgCl_2 \cdot 6 H_2O$ in a lightly screw-capped 2.8 L baffled Fernbach flask (Corning) was allowed to equilibrate overnight at 37 °C. Just prior to inoculation with 40 mL of starter culture (2%), the media was supplemented with 20 mM glucose and 50 μg mL^{-1} of Km and Cm. The culture was grown

at 37 °C at 180 rpm to OD_{600} = 0.5–0.6, at which point 1 mM L-cysteine • HCl and 500 μM $(NH_4)_2Fe(II)(SO_4)_2 \cdot 6 H_2O$ were added. Cultures were cooled on ice for 20 min and induced with 500 μM IPTG. The flask was immediately tightly sealed with a septa and screw cap before growing overnight at 16 °C at 180 rpm. Cell pellets were harvested aerobically by centrifugation at 6,730 × g for 10 min at 4 °C, flash frozen in N_2 (*l*), and stored at –80 °C.

All subsequent purification steps were performed in an anaerobic chamber at 4 °C containing 97% N_2 and 3% H_2 (CoyLabs). *Cs* HPAD was purified using buffers A1 and A2 as described above. Frozen cell pellets were thawed, resuspended at 5 mL per g cell paste with anoxic Buffer B1 (25 mM Tris, 200 mM NaCl, pH 9) (all AEs) supplemented with 10 mg L^{-1} of chicken egg lysozyme, 10 mg L^{-1} DNase I, 10 mM $MgCl_2 \cdot 6 H_2O$, 1 mM β-mercaptoethanol (BME), and SIGMA*FAST* Protease Inhibitor Cocktail Tablets, EDTA-Free. Cells were lysed by sonicating with a ½ inch horn at 30% amplitude for 6 min (2.5 s on followed by 5 s off). The resulting suspension became dark grey during lysis. Lysate was transferred into a 50 mL centrifuge tube, taken out of the anaerobic chamber, and centrifuged at 20,000 × g for 30 min at 4 °C to separate soluble and insoluble fractions before being brought back into the anaerobic chamber. The soluble lysate loaded onto a Ni-NTA column (3 mL resuspended resin per 2 L expressed, pre-equilibrated with anoxic Buffer B1) by gravity flow. The column was washed with 10 c.v. of 90% Buffer B1, 10% Buffer B2 (25 mM Tris, 200 mM NaCl, 250 mM imidazole, pH 9), 5 c.v. of 80% Buffer B1, 20% Buffer B2, and 5 c.v. of 70% Buffer B1, 30% Buffer B2. Protein was eluted from the column with Buffer B2 in 3 mL fractions. Brown elution fractions containing protein, as detected by Bradford, were combined, transferred to a Slide-a-Lyzer™ dialysis cassette with a 10 kDa MWCO (Thermo Scientific), and dialyzed three times against 1 L of Buffer B1 supplemented with 10% glycerol. After dialysis, the solution was concentrated using a Ultra-15 Centrifugal Filters centrifugal concentrator with a 10 kDa MWCO membrane (Amicon) to a protein concentration of ~200 μM. Protein concentrations were estimated with using NanoDrop 2000 UV-Vis Spectrophotometer (Thermo Scientific) using ε_{280nm} calculated by ExPASY ProtParam[21] as follows: *Ou* IAD-AE (ε = 21,890 M^{-1} cm^{-1}); *Cs* HPAD-AE (ε = 57,870 M^{-1} cm^{-1}); Cs HPAD (ε = 140,500 and 12,950 M^{-1} cm^{-1}). The protein solution was aliquoted into 0.5 mL cryogenic vials and placed in 18 × 150 mm Balch-type tubes (Chemglass). The tubes were sealed with butyl stoppers and aluminum seals, frozen in N_2 (*l*), and stored at –80 °C.

Glycyl radical quantification by electron paramagnetic resonance (EPR) spectroscopy

Much effort went into optimizing conditions for glycyl radical installation on *Ou* IAD by *Ou* IAD-AE. In brief various reductants (sodium dithionite ± methyl viologen, titanium citrate ± methyl viologen, acriflavine ± halogen lamp exposure, sodium ascorbate), length of halogen lamp illumination (0–2 h), buffer components (buffer identity, pH, NaCl concentrations), IAD:IAD-AE ratios, and FeS reconstitution of IAD-AE.[22] None of these optimizations improved *Ou* IAD activity relative to the conditions described below.

Wild-type *Ou* IAD and mutants were prepared for EPR spectroscopy as follows. All assay concentrations are final concentrations. Anoxic *Ou* IAD and *Ou* IAD-AE aliquots were brought into an anaerobic chamber containing N_2 and < 0.1 ppm O_2 at 22 °C (Mbraun). 10 mM DTT, 100 μM 5-deazariboflavin,[23] 50 μM *Ou* IAD-AE, 50 μM *Ou* IAD (total monomer concentration), and 500 μM SAM were mixed together in buffer (50 mM HEPES, 50 mM NaCl, pH 8.0). The samples were illuminated by a 500 W halogen lamp for 30 min to photoreduce the $[Fe_4S_4]$ cluster from the +2 to +1 state.[24] The entire 250 μL reaction was used for analysis.

Perpendicular mode X-band EPR spectra were recorded on an EMX-Plus EPR. All samples were loaded into EPR tubes with 4 mm outer diameter and 8″ length (Wilmad LabGlass, 734-LPV-7), sealed, and frozen in N_2 (*l*). Data acquisition was performed with Xepr software (Bruker). The magnetic field was calibrated with an external standard of $K_2(SO_3)_2NO$ (Frémy) [g_x = 2.00785, g_y = 2.00590, g_z = 2.00265, A_x = 5.5 G, A_y = 5.0 G, A_z = 28.7 G].[25] The experimental spectra for the glycyl radicals were modeled with EasySpin (Version 5.2.33)[26] for MATLAB (MathWorks) to obtain *g*-values, hyperfine coupling constants, and line widths. Spin concentration measurements were performed by numerically calculating the double integral of the simulated spectra and comparing the area with that of a Frémy standard. This standard was prepared

before each set of EPR measurements by dissolving solid Frémy under anaerobic conditions in anoxic 20 mM Tris, 100 mM KCl, pH 7.5 and diluting to a final concentration of 25 μM. To account for any decomposition during dissolution, the concentration was measured at 248 nm (ε = 1,690 M^{-1} cm^{-1}) using a NanoDrop 2000 UV-Vis Spectrophotometer.[27] EPR spectra were recorded under the following conditions: temperature, 77 K; center field, 3350 G; sweep width, 200 G; microwave power, 1.262 μW; microwave frequency, 9.37 GHz; modulation amplitude, 4 G; modulation frequency, 100 kHz; time constant, 0.01 ms; conversion time, 20.12 ms; sweep time, 10.06 s; receiver gain, 52 dB. Simulated spectra were integrated twice to quantify the number of spins in each sample. All EPR assays were performed in triplicate.

Iron and sulfide quantification and UV-vis assays

The iron content of a 6 μM solution of *Ou* IAD-AE was determined using Ferene (3-(2-Pyridyl)-5,6-di(2-furyl)-1,2,4-triazine-5′,5″-disulfonic acid disodium salt), according to a previously published procedure,[28] with the only differences being that the assay volume was tripled, the standard curve was prepared with $(NH_4)_2Fe(II)(SO_4)_2$, • 6 H_2O and samples were incubated at 37 °C for 1 h after the addition of Solution B.

The sulfide content of a 6 μM solution of *Ou* IAD-AE was determined using a previously published procedure,[29] with the only differences being that assays were performed in microcentrifuge tubes, assay volumes were doubled, pipetting was performed instead of stirring, and the mixture was incubated for 20 min after the addition of NaOH.

To obtain UV-vis spectra, *Ou* IAD-AE was diluted to 25 μM with anoxic buffer (50 mM HEPES, 50 mM NaCl, pH 8.0) inside of an anaerobic chamber at 4 °C containing 97% N_2 and 3% H_2 (CoyLabs). The absorbance of the solution was measured from 200 nm to 1200 nm in a septa-sealed Ultra-Micro Cell quartz cuvette (Hellma) using a Cary 8454 UV-Vis Diode Array System (Agilent). To obtain a spectrum for the reduced protein, 100 μM sodium dithionite was added with a gas-tight syringe before the absorbance was measured at 1, 4, 10, 25, and 30 min.

Generation of multiple sequence alignment, homology models, and substrate docked models

Multiple sequence alignment (MSA) of *Olsenella uli* IAD (Uniprot ID: E1QXZ2), *Olsenella scatoligenes* IAD (Uniprot ID: A0A100YXA1), *Clostridium scatologenes* HPAD (Uniprot ID: A0A0E3JS98), *Clostridioides difficile* HPAD (Uniprot ID: C9YHW1), PAD from sewage (Uniprot ID: A0A2P1UAH0), and Olsenella scatoligenes AAD (Uniprot ID: A0A100YWM3) was created using the default parameters of Clustal Omega[17] with a Pearson/FASTA output format, and the results were visualized in Geneious Pro 11.0.4 (Biomatters).[16]

Homology models of *Ou* IAD and PAD were generated using the SWISS-MODEL[1] workspace. The most similar, structurally solved proteins (HPA bound *Cs* HPAD, PDB ID: 2YAJ and *Roseburia inulinivorans* 1,2-propanediol, PDB ID: 5I2A, respectively) were used as templates for homology model construction. In addition, homology models of *Ou* IAD were created using MOE 2020.0901 (Chemical Computing Group) and AlphaFold2 with MMseqs2.[2, 3] These structures were corrected in MOE, energy minimized, protonated to pH 8, and structurally aligned against PDB ID: 2YAJ. I3A was docked into the active site using Triangle Matcher Placement method, London dG scoring, 30 poses. Refinement was performed using Induced Fit method, GBVI/WSA dG scoring, 5 poses. Structures were visualized and compared in Pymol.

UPLC–MS/MS assays for detecting end-point SAM cleavage products

LC–MS samples were prepared by combining 10 mM DTT, 10 μM 5-deazariboflavin, 5 μM *Ou* IAD-AE, 5 μM *Ou* IAD (total monomer concentration), and 500 μM SAM in anoxic buffer (50 mM HEPES, 50 mM NaCl, pH 8.0) in an anaerobic chamber containing N_2 and < 0.1 ppm O_2 at 22 °C (Mbraun) or 3% H_2 and 97% N_2 (CoyLabs). The samples were illuminated by a 500 W halogen lamp for 30 min prior to the addition of 500 μM indole-3-acetate (I3A). After incubating at room temperature for 2.5 h, the reactions were taken out of

the anaerobic chamber and quenched with 5 equivalents of LC–MS grade acetonitrile (ACN) with 50 μM adenosine internal standard. The samples were place in a –20 °C freezer for 30 min, centrifuged at 3,220 × g for 10 min, 4 °C on a tabletop swinging bucket centrifuge, diluted 4-fold into H_2O, and then diluted a further 20-fold into H_2O.

Simultaneous analysis of SAM, 5′-deoxyadenosine (5′-dA), *S*-adenosyl-L-homocysteine (SAH), methylthioadenosine (MTA), and adenosine was carried out by ultra performance liquid chromatography tandem mass spectrometry (UPLC–MS/MS). Liquid chromatography was conducted using a Waters Acquity UPLC H-Class System (Waters Corporation). 1 μL of each sample was injected onto an Acquity UPLC BEH C18 1.7 μm (2.1 × 50 mm) column (Waters Corporation). The flow rate was 0.8 mL min^{-1} using mobile phase A = 0.1% formic acid in H_2O and mobile phase B = 0.1% formic acid in ACN. The column temperature was maintained at 40 °C. The following gradient was applied: 0–1 min at 0–33% B isocratic, 1.0–1.5 min at 33–100% B, 1.5–2.0 min at 100% B isocratic, 2.0–2.1 min at 100–0% B, 2.1–2.8 min at 0% B isocratic. The first 0.4 min of the run was diverted to waste. MS detection was performed with a Waters Xevo TQ-S (Waters Corporation) instrument with electron spray ionization in positive mode (ESI^+) (capillary voltage, 0.7 kV; cone voltage, 15 V; source offset voltage, 50 V; desolvation temperature, 500 °C; desolvation gas flow, 1000 L h^{-1}; cone gas flow, 150 L h^{-1}; nebulizer, 7.0 bar). Conditions for tandem MS/MS were optimized using authentic standards. See **Table S3** for specific detection parameters. For quantification, standards of 5′-dA, SAH, and MTA were prepared ranging from 50–600 μM in H_2O, in triplicate.

UPLC–MS/MS assays for detecting I3A, skatole, and I3A analogs

LC–MS samples were prepared in the same way as the LC–MS samples for detecting SAM cleavage products, except that they were quenched by the addition of 5 equivalents of LC–MS grade ACN with 50 μM D_3-skatole and 50 μM D_7-I3A as internal standards. The samples were place in a –20 °C freezer for 30 min, centrifuged at 3,220 × g for 10 min, 4 °C on a tabletop swinging bucket centrifuge, and diluted 4-fold into H_2O.

Simultaneous analysis of I3A, skatole, and I3A analogs was carried out by UPLC–MS/MS. Liquid chromatography was conducted using a Waters Acquity UPLC H-Class System (Waters Corporation). 1 μL of each sample was injected onto an Acquity UPLC BEH C18 1.7 μm (2.1 × 50 mm) column (Waters Corporation). The flow rate was 0.5 mL min^{-1} using mobile phase A = 0.1% formic acid in H_2O and mobile phase B = 0.1% formic acid in ACN. The column temperature was maintained at 40 °C. The following gradient was applied: 0–0.4 min at 10% B isocratic, 0.4–1.3 min at 10–80% B, 1.3–2.1 min at 80% B isocratic, 2.1–2.3 min at 80–10% B, 2.3–3.4 min at 10% B isocratic. The first 0.4 min of the run was diverted to waste. MS detection was performed with a Waters Xevo TQ-S (Waters Corporation) instrument with atmospheric pressure chemical ionization in positive mode ($APCI^+$) (capillary voltage, 3.10 kV; cone voltage, 42 V; source offset voltage, 50 V; desolvation temperature, 500 °C; desolvation gas flow, 800 L h^{-1}; cone gas flow, 150 L h^{-1}; nebulizer, 7.0 bar). Conditions for tandem MS/MS were optimized using authentic standards, unless otherwise stated. See **Table S3** for specific detection parameters. For quantification, standards were prepared ranging from 50–600 μM in H_2O, in triplicate.

Table S3. UPLC–MS/MS analysis of standards used for assays.

Standard	*Transition (m/z)*	*Mode*	*Cone (V)*	*Collision (V)*	*Retention (min)*	*Notes*
5′-Deoxyadenosine (5′-dA)	252.0062 > 135.9766	ESI	2	14	0.60	
S-Adenosyl-L-homocysteine (SAH)	399.1181 > 250.0088	ESI	2	12	0.47	
Methylthioadenosine (MTA)	297.9942 > 162.9676	ESI	62	12	0.95	
Adenosine (internal standard)	268.0010 > 135.9765	ESI	4	14	0.60	
Indole (internal standard)	118.0259 > 91.0398	APCI	70	18	1.92	
Skatole	132.0404 > 116.9679	APCI	32	18	2.03	
D_1-Skatole_SAME	133.0479 > 117.2786	APCI	32	18	2.03	Not opt.
D_2-Skatole_SAME	134.0542 > 117.2786	APCI	32	18	2.03	Not opt.
D_3-Skatole_SAME	135.0604 > 117.0599	APCI	32	18	2.03	Not opt.

D_3-Skatole (internal standard)	135.0604 > 117.0599	APCI	60	32	2.03	
Indole-3-acetic acid (I3A)	176.0042 > 129.9714	APCI	30	20	1.69	
D_1-I3A_SAME	177.0769 > 131.0714	APCI	30	20	1.69	Not opt.
D_2-I3A_SAME	178.0440 > 132.0833	APCI	30	20	1.69	Not opt.
D_7-I3A (internal standard)	183.0319 > 135.8457	APCI	44	12	1.69	
Indole-3-carbinol (I3C)	130.032 > 77.006	APCI	68	20	1.56	
Indole-3-ethanol (I3Et)	144.032 > 117.058	APCI	88	16	1.70	
1-Methylindole-3-acetic acid (1-Me-I3A)	190.160 > 144.092	APCI	52	14	1.83	
2-Methylindole-3-acetic acid (2-Me-I3A)	190.0470 > 144.030	APCI	42	16	1.75	
4-Chloroindole-3-acetic acid (4-Cl-I3A)	209.924 > 163.977	APCI	42	16	1.79	
5-Bromoindole-3-acetic acid (5-Br-I3A)	254.032 > 207.976	APCI	22	16	1.85	
5-Methylindole-3-acetic acid (5-Me-I3A)	190.032 > 144.094	APCI	14	16	1.80	
5-Hydroxyindole-3-acetic acid (5-OH-I3A)	193.032 > 146.026	APCI	22	14	0.86	
5-Methoxyindole-3-acetic acid (5-OMe-I3A)	206.096 > 160.083	APCI	30	18	1.65	
6-Fluoroindole-3-acetic acid (6-F-I3A)	193.954 > 147.959	APCI	32	14	1.75	
7-Methylindole-3-acetic acid (7-Me-I3A)	190.047 > 144.105	APCI	8	14	1.78	
α,α-Dimethyl-indole-3-acetic acid (α,α-Me_2-I3A)	204.190 > 158.220	APCI	34	12	1.84	
α-Methyl-indole-3-acetic acid (α-Me-I3A)	190.111 > 144.099	APCI	30	14	1.82	

HPLC assay for detecting I3A, skatole, and I3A analogs

HPLC samples were prepared in the same way as the UPLC–MS samples for detecting SAM cleavage products except that they were subsequently quenched by the addition of 17 μL of 5% HPLC-grade trifluoroacetic acid (TFA) in LC–MS grade acetonitrile. The supernatant was analyzed by HPLC on an Inspire C18 column (5 μm particle size, 50 × 4.6 mm) (Dikma Technologies). 20 μL of each sample was injected onto the column. The flow rate was 1 mL min^{-1} using 0.1% formic acid in water as mobile phase A and 0.1% formic acid in acetonitrile as mobile phase B. The column was maintained at 25 °C. The following gradient was applied: 0–3 min: 5% B isocratic, 3–10 min: 5–90% B, 10–12 min: 90% B isocratic, 12–3 min: 90–5% B, 13–16 min: 55% B isocratic. All compounds were detected by measuring the absorbance at 280 nm. See **Table S4** for specific detection parameters. For quantification, standards were prepared ranging from 50–600 μM in H_2O, in singlicate.

Table S4. HPLC analysis of standards used for assays.

Standard	***Retention (min)***
Skatole	7.52
Indole-3-acetic acid (I3A)	5.96
Indole-3-acetamide (I3Ac)	4.37
Indole-3-acetone (I3At)	6.50
Indole-3-acetonitrile (I3ACN)	6.59
Indole-3-glyoxylic acid (I3G)	4.22
Benzofuran-3-acetic acid (BF3A)	6.62
Benzothiophene-3-acetic acid (BT3A)	6.97
Naphthalene-2-acetic acid (N2A)	7.16

UPLC–MS/MS assay for WT and H514A IAD Michaelis–Menten kinetics

All assay concentrations are final concentrations. Anoxic *Ou* IAD and *Ou* IAD-AE aliquots were brought into anaerobic chamber containing N_2 and < 0.1 ppm O_2 at 22 °C (Mbraun). An activation mixture containing 10 mM DTT, 100 μM 5-deazariboflavin, 50 μM O*u* IAD-AE, 50 μM *Ou* IAD, and 500 μM SAM in anoxic buffer (50 mM HEPES, 50 mM NaCl, pH 8.0) was illuminated by a 500 W halogen lamp for 30 min to install the glycyl radical on IAD. Assays were performed in PCR strips (VWR International) containing pre-aliquoted solutions of D_0-I3A or D_2-I3A (and inhibitor) prepared in buffer. 1 min prior to beginning kinetics, activated WT IAD was diluted to 4.0 μM. Reactions were initiated by adding an equivalent volume of diluted IAD (2.0 μM final for WT IAD, 25 μM final for H514A IAD). Time points (10, 20, 30, 40, 50, 60 s) were obtained by quenching 10 μL of the reaction mixture with 50 μL of LC–MS grade acetonitrile containing 50 μM D_3-skatole internal standard in pre-aliquoted PCR strips. The sealed PCR strips were taken out of the anaerobic chamber and analyzed by UPLC–MS/MS as previously described. All of the data were fit to the

Michaelis–Menten equation simultaneously using nonlinear regression in Graphpad Prism. Experiments were repeated on two independent days with similar results.

UPLC–MS/MS for isotope enrichment assays to determine kinetic isotope effect (KIE)

50 μM *Ou* IAD was activated as previously described. Assays were performed in PCR strips (VWR International) containing pre-aliquoted solutions of 500 μM D_0-I3A and D_2-I3A; D_0-I3A and D_7-I3A; or D_2-I3A and D_7-I3A prepared in buffer (50 mM HEPES [8], 50 mM NaCl). 1 min prior to beginning kinetics, activated IAD was diluted to 8.0 μM. Reactions were initiated by adding an equivalent volume of diluted IAD (4.0 μM final). Time points (10, 20, 30, 40, 50, 60, 90, 120, 150, 300, 450, 600, 900, 1200, 1500 s) were obtained by quenching 10 μL of the reaction mixture with 50 μL of LC–MS grade acetonitrile containing 50 μM D_3-skatole internal standard and either 50 μM D_7-I3A, D_2-I3A, or D_0-I3A in pre-aliquoted PCR strips. The sealed PCR strips were taken out of the anaerobic chamber and analyzed by UPLC–MS/MS as previously described.

Kinetic isotope effects (KIEs) from competition assays were determined using the following equation[30]:

$$\frac{k_H}{k_D} = \frac{\ln(1-F)}{\ln((1-F)\frac{R}{R_0})}, \text{ where } F = \frac{[SM_H]}{[SM_H]_0} \text{ and } R = \frac{[SM_D]}{[SM_H]}$$

Where k_H and k_D are the consumption rates of D_0-I3A and D_2-I3A, respectively; $[SM_H]$ and $[SM_D]$ are the concentrations of D_0-I3A and D_2-I3A, respectively; and $[SM_H]_0$ is the concentration of D_0-I3A at time zero. The KIE of each biological triplicate was determined when at least half of the substrate was consumed and then averaged. Assays were repeated on two independent days with similar results (**Figure S8**).

UPLC–MS/MS assay for IAD incubations in D_2O

50 μM *Ou* IAD was activated as previously described. Assays were performed in PCR strips (VWR International) containing pre-aliquoted solutions of 1 mM D_0-I3A, D_2-I3A, or skatole in various ratios of buffer (50 mM HEPES [8], 50 mM NaCl) in H_2O:D_2O. Activated IAD was added (2.5 μM final) and the reactions were incubated for 4.5 hr. The sealed PCR strips were taken out of the anaerobic chamber, quenched with 5 equivalents of LC–MS grade acetonitrile containing 50 μM D_7-I3A and 50 μM indole internal standards and analyzed by UPLC–MS/MS as previously described.

UHPLC–MS assay for HPAD incubations in D_2O

UHPLC–MS samples were prepared by combining 10 mM DTT, 2 mM sodium dithionite, 5 μM *Cs* HPAD-AE, 5 μM *Cs* HPAD (total monomer concentration), 500 μM SAM, and 25 mM hydroxyphenylacetate in anoxic buffer (50 mM HEPES, 50 mM NaCl, pH 8.0) in an anaerobic chamber containing N_2 and < 0.1 ppm O_2 at 22 °C (Mbraun). The reactions were incubated for 19 hr prior to being passed through a Filter Nanosep® Centrifugal Devices with Omega™ Membrane 10K and analyzed by UHPLC–MS/MS.

Analysis of hydroxyphenylacetate and *p*-cresol was carried out on a Thermo Scientific Dionex UltiMate 3000 UHPLC coupled to a Thermo Q Exactive Plus mass spectrometer system (Thermo Fisher Scientific Inc, Waltham, MA) equipped with an APCI probe for the Ion Max API source. Data were acquired with Chromeleon Xpress software for UHPLC and Thermo Xcalibur software version 3.0.63 for mass spectrometry and processed with Thermo Xcalibur Qual Browser software version 4.0.27.19.

8 μL sample was injected onto the UHPLC including an HPG-3400RS binary pump with a built-in vacuum degasser and a thermostated WPS-3000TRS high performance autosampler. An Xterra MS C18 analytical column (2.1 × 50 mm, 3.5 μm) from Waters Corporation (Milford, MA) was used at the flow rate of 0.3 mL min^{-1} using 0.1% formic acid in water as mobile phase A and 0.1% formic acid in acetonitrile as mobile phase B. The column temperature was maintained at room temperature. The following gradient was applied:

0–5 min: 5–47 %B, 5–5.1 min: 47–100 %B, 5.1–7.1 min: 100 %B isocratic, 7.1–7.2 min: 100–5% B, 7.2–9.2min, 5 %B isocratic.

The MS conditions were as follows: negative ionization mode; scan range, 85–165 *m/z*; resolution, 140,000; AGC target, 1e6; maximum IT, 480 ms; spray voltage, 5000 V; capillary temperature, 325 °C; sheath gas, 28; Aux gas, 5; maximum spray current, 5; probe heater temperature, 432 °C; S-Lens RF level, 55.00. A mass window of ± 5 ppm was used to extract the ion of $[M–H]^–$ for the compounds. Targets were considered detected when the mass accuracy was less than 5 ppm and there was a match of isotopic pattern between the observed and the theoretical ones and a match of retention time between those in real samples and standards.

Computational details

Electronic structure calculations were performed to investigate the energetics of substrates, intermediates, and products along the hypothesized intrinsic reaction pathways (*i.e.*, without explicitly modeling the enzyme environment) using ORCA[31] 4.0.1.2. Small models of the key residues (Cys500, Glu502, His514 in the case of *Ou* IAD and Cys503, Glu505, Glu637 in the case of *Cs* HPAD) in which backbone atoms are replaced with a methyl group were used. Geometry optimizations were performed using density functional theory (DFT) with the hybrid B3LYP[32] functional in conjunction with the 6-31G(d) basis in the gas phase. To obtain more accurate reference energetics, corrected single-point energies were obtained with domain local pair natural orbital (PNO) coupled-cluster calculations [*i.e.*, DLPNO-CCSD(T)].[33] Because implicit solvent models are not implemented in conjunction with DLPNO-CCSD(T), we corrected gas-phase DLPNO-CCSD(T) intermediate energies with the conductor-like implicit solvent model (C-PCM)[34, 35] with dielectric constant of 10 obtained from MP2[36] in ORCA.

Table S5. Thermodynamic corrections to the electronic energy (Eel) in kcal mol–1 for IAD. These results were obtained with B3LYP/6-31G(d)-optimized geometries of $CH_3S•$, CH_3SH, acetate, indole-3-acetate + acetic acid (ET-R and HAT-R), indole-3-acetate + acetic acid + 5-methylimidazole (ET/PT-R), indole-3-acetate (radical-anion) + 4-methylimidazole (ET/PT-Int1), indole-3-acetate radical (ET-Int1), indole-3-•$CHCO_2$ + acetic acid (HAT-Int1), acetic acid + $CH_3S^–$, acetate + CH_3SH, indole-3-methylene radical + 5-methylimidazole (ET/PT-Int2), indole-3-methylene radical (ET-Int2 and HAT-Int2), skatole + 5-methylimidazole (ET/PT-P), skatole (ET-P and HAT-P). Zero point energy (ZPE) (column 2), ZPE and thermal energy (column 3), entropy multiplied by temperature (TS, where T = 298.15 K; column 4), and the energy that is to be added to E_{el} to transform it into G^0 (G – E_{el}; column 5) in kcal $mol^{–1}$ are shown. Inner energy, U = ZPE + thermal energy + E_{el}. G – E_{el} = ZPE, T = 298.15 K.

**System**	**ZPE (kcal $mol^{–1}$)**	**ZPE + thermal energy (U – E_{el}) (kcal $mol^{–1}$)**	**Entropy (TS) (kcal $mol^{–1}$)**	**G – E_{el} (H – TS – E_{el} = U + kBT – TS – E_{el} = ZPE + thermal + kBT – TS) (kcal $mol^{–1}$)**
$CH_3S•$	22.82	24.77	17.11	8.25
CH_3SH	29.07	31.33	17.42	14.50
acetate	30.22	32.47	18.46	14.60
indole-3-acetate + acetic acid (ET-R and HAT-R)	138.76	147.60	34.47	113.72
indole-3-acetate + acetic acid + 5-methylimidazole (ET/PT-R)	201.42	213.88	42.52	171.96
indole-3-acetate (radical-anion) + 4-methylimidazole (ET/PT-Int1)	153.81	162.17	33.91	128.86
indole-3-acetate radical (ET-Int1)	98.87	105.01	28.58	77.03
indole-3-•$CHCO_2$ + acetic acid (HAT-Int1)	130.28	139.62	36.23	103.98
acetic acid + $CH_3S^–$	60.88	66.41	26.87	40.14
acetate + CH_3SH	59.73	64.15	24.70	40.04
indole-3-methylene radical + 5-methylimidazole (ET/PT-Int2)	153.50	161.93	33.99	128.54
indole-3-methylene radical (ET-Int2 and HAT-Int2)	90.33	95.22	25.26	70.55
skatole + 5-methylimidazole (ET/PT-P)	162.04	171.78	37.20	135.18

skatole (ET-P and HAT-P)	98.89	103.99	25.44	79.14

Table S6. Relative gas-phase DLPNO-CCSD(T)/CBS electronic energies for IAD. In column 2, the electronic energies are shown; in column 3, solvent corrected DLPNO-CCSD(T)/CBS energies with solvent correction energies obtained at the MP2/CBS level of theory for dielectric values of 10 are shown; and in column 4, Gibbs free energies at T = 298.15 K and 1 atm pressure for dielectric values of are shown. All energies reported here are in units of kcal mol^{-1}. The two-point extrapolation formula based on the aug-cc-pVDZ and aug-cc-pVTZ energies is used to extrapolate to the complete basis set limit for DLPNO-CCSD(T). DLPNO-CCSD(T)/CBS computed using tight PNO thresholds, which refer to the default thresholds of energies were TCutPairs = 10^{-5}, TCutPNO = 10^{-10}, and TCutMKN = 10^{-3}.

System	DLPNO-CCSD(T)/CBS (kcal mol^{-1})	DLPNO-CCSD(T)/CBS + solv. corr. with ε = 10 (kcal mol^{-1})	DLPNO-CCSD(T)/CBS + solv. corr. with ε = 10 + G – E_{el} (kcal mol^{-1})
CH_3S•	–274586.03	–274588.03	–274579.78
CH_3SH	–274992.02	–274994.56	–274980.06
acetate	–143262.13	–143321.88	–143307.28
indole-3-acetate+acetic acid (ET-R and HAT-R)	–514152.32	–514205.03	–514091.31
indole-3-acetate+acetic acid+5-methylimidazole (ET/PT-R)	–680580.52	–680640.42	–680468.46
indole-3-acetate (radical-anion) + 4-methylimidazole (ET/PT-Int1)	–536546.41	–536592.7	–536463.84
indole-3-acetate radical (ET-Int1)	–370414.36	–370443.79	–370366.76
indole-3-•$CHCO_2$ + acetic acid (HAT-Int1)	–513741.94	–513802.16	–513698.18
acetic acid + CH_3S^-	–418269.2	–418319.59	–418279.45
acetate + CH_3SH	–418257.64	–418313.95	–418273.91
indole-3-methylene radical + 5-methylimidazole (ET/PT-Int2)	–418653.58	–418641.61	–418513.07
indole-3-methylene radical (ET-Int2 and HAT-Int2)	–252221.2	–252229.14	–252158.59
skatole + 5-methylimidazole (ET/PT-P)	–419066.11	–419076.44	–418941.26
skatole (ET-P and HAT-P)	–252633.01	–252638.83	–252559.69

Table S7. Thermodynamic corrections to the electronic energy (Eel) in kcal mol–1 for HPAD. These results were obtained with B3LYP/6-31G(d)-optimized geometries of hydroxyphenylacetate + acetic acid (ET-R and HAT-R), hydroxyphenylacetate + acetic acid + acetate (ET/PT-R), hydroxyphenylacetate (radical-anion) + 4-methylimidazole (ET/PT-Int1), hydroxyphenylacetate radical (ET-Int1), hydroxyphenyl-•$CHCO_2$ + acetic acid (HAT-Int1), hydroxyphenyl-methylene radical + 5-methylimidazole (ET/PT-Int2), hydroxyphenyl-methylene radical (ET-Int2 and HAT-Int2), *p*-cresol + 5-methylimidazole (ET/PT-P), *p*-cresol (ET-P and HAT-P). Zero point energy (ZPE) (column 2), ZPE and thermal energy (column 3), entropy multiplied by temperature (TS, where T = 298.15 K; column 4), and the energy that is to be added to E_{el} to transform it into G^0 (G – E_{el}; column 5) in kcal mol^{-1} are shown. Inner energy, U = ZPE + thermal energy + E_{el}. G – E_{el} = ZPE, T = 298.15 K.

System	ZPE (kcal mol^{-1})	ZPE + thermal energy (U – E_{el}) (kcal mol^{-1})	Entropy (TS) (kcal mol^{-1})	G – E_{el} (H – TS – E_{el} = U + kBT – TS – E_{el} = ZPE + thermal + kBT – TS) (kcal mol^{-1})
hydroxyphenylacetate + acetic acid (ET-R and HAT-R)	123.25	132.26	34.72	98.13
hydroxyphenylacetate + acetic acid + acetate (ET/PT-R)	155.08	165.64	38.79	127.44
hydroxyphenylacetate (radical-anion) + 4-methylimidazole (ET/PT-Int1)	116.83	125.08	33.61	92.07
hydroxyphenylacetate radical (ET-Int1)	84.05	90.16	28.37	62.38
hydroxyphenyl-•$CHCO_2$ + acetic acid (HAT-Int1)	115.80	124.54	34.64	90.49
hydroxyphenyl-methylene radical + 5-methylimidazole (ET/PT-Int2)	106.26	113.16	30.48	83.27
hydroxyphenyl-methylene radical (ET-Int2 and HAT-Int2)	74.71	78.99	23.75	55.83
p-cresol + 5-methylimidazole (ET/PT-P)	114.86	121.57	29.92	92.25
p-cresol (ET-P and HAT-P)	83.00	87.64	24.42	63.81

Table S8. Relative gas-phase energies relevant to the HPAD system. DLPNO-CCSD(T)/CBS electronic energies (column 2), solvent corrected DLPNO-CCSD(T)/CBS energies with solvent correction energies obtained at the MP2/CBS level of theory for dielectric values of 10 (column 3), Gibbs free energies at T = 298.15 K and 1 atm pressure for dielectric values of 10 (column 4) are shown. All energies reported here are in units of kcal mol^{-1}. The two-point extrapolation formula based on the aug-cc-pVDZ and aug-cc-pVTZ energies is used to extrapolate to the complete basis set limit for DLPNO-CCSD(T). DLPNO-CCSD(T)/CBS computed using tight PNO thresholds, which refer to the default thresholds of energies were TCutPairs = 10^{-5}, TCutPNO = 10^{-7}, and TCutMKN = 10^{-3}.

System	DLPNO-CCSD(T)/CBS (kcal mol^{-1})	DLPNO-CCSD(T)/CBS + solv. corr. with ε = 10 (kcal mol^{-1})	DLPNO-CCSD(T)/CBS + solv. corr. with ε = 10 + G – E_{el} (kcal mol^{-1})
hydroxyphenylacetate + acetic acid (ET-R and HAT-R)	–478852.5	–478902.72	–478804.59
hydroxyphenylacetate + acetic acid + acetate (ET/PT-R)	–622071	–622209.51	–622082.07
hydroxyphenylacetate (radical-anion) + 4-methylimidazole (ET/PT-Int1)	–478436.85	–478481.29	–478389.22
hydroxyphenylacetate radical (ET-Int1)	–335133.57	–335158.96	–335096.58
hydroxyphenyl-•$CHCO_2$ + acetic acid (HAT-Int1)	–478441.02	–478494.46	–478403.97
hydroxyphenyl-methylene radical + 5-methylimidazole (ET/PT-Int2)	–360208.16	–360254	–360170.73
hydroxyphenyl-methylene radical (ET-Int2 and HAT-Int2)	–216929.19	–216935.67	–216879.84
p-cresol + 5-methylimidazole (ET/PT-P)	–360616.91	–360665.78	–360573.53
p-cresol (ET-P and HAT-P)	–217340.69	–217346.35	–217282.54

Table S9. Reaction free energies and free energy barriers comparing I3A and α-I3A reactivity. Level of theory that used for these calculations is B3LYP/6-311++g(d,p)//6-31G(d) and dielectric constant = 10. Energies are reported in kcal mol^{-1}.

Substituents	ΔG (HAT)	$ΔG^‡$ (HAT)
With adjacent methyl group	–6.2	14.6
Without methyl group	–4.4	14.7

Synthesis of α-methyl-indole-3-acetic acid and D_3-skatole

All chemicals and solvents were purchased from Sigma-Aldrich unless otherwise noted. Anhydrous reactions were performed using oven dried or flame-dried glassware, which were then cooled under vacuum and purged with nitrogen (N_2) gas. Unless otherwise noted, all proton nuclear magnetic resonance (^1H NMR) spectra and carbon nuclear magnetic resonance (^{13}C NMR) spectra were recorded on a Bruker AVANCE NEO 400 (400 MHz, 100 MHz) NMR spectrometer.

Chemical shifts (δ) are reported in parts per million (ppm) using the solvent resonance as an internal standard for ^1H ($CDCl_3$ = 7.26 ppm, C_2D_6OS = 2.50 ppm and ^{13}C ($CDCl_3$ = 77.2 ppm, C_2D_6OS = 39.5 ppm). Data are reported as follows: chemical shift, multiplicity (br = broad, s = singlet, d = doublet, t = triplet, q = quartet, m = multiplet), coupling constants in Hertz (Hz), integration, and assignments. All NMR solvents were purchased from Cambridge Isotope Laboratories unless otherwise noted. NMR spectra were visualized using MestReNova, version 12.0.3-21384.

Mass spectrometry data were obtained using an Agilent 6530 quadruple time-of-flight mass spectrometer with an ESI source. The mass spectra data were recorded on positive ionization mode with a mass range of 100 to 3000 *m/z*; spectra rate, 10 spectra s^{-1}; capillary voltage, 4500 V; nebulizer pressure, 22 psi; drying gas (N_2) flow, 8 L min^{-1}; temperature, 200 °C.

Synthesis of methyl 2-(1*H*-indol-3-yl)acetate (21)

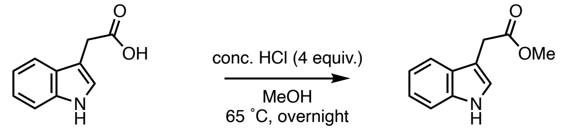

Indole-3-acetic acid **21**

Methyl 2-(1*H*-indol-3-yl)acetate (**21**) was synthesized according to a published protocol[37] with the following modifications. Indole-3-acetic acid (2 g, 11.417 mmol, 1 eq.) was dissolved in MeOH (110 mL). Concentrated hydrochloric acid (HCl) (3.806 mL, 45.667 mmol, 4.0 eq.) was added at room temperature. The mixture was refluxed overnight at 65°C for 14 h, cooled to room temperature, and concentrated *in vacuo*. The residue was dissolved in saturated sodium bicarbonate ($NaHCO_3$) and then extracted with diethyl ether (Et_2O) three times. The combined organic extracts were washed with brine, dried over anhydrous sodium sulfate (Na_2SO_4), filtered, and concentrated *in vacuo* to afford methyl 2-(1*H*-indol-3-yl)acetate as a brown oil (2.139 g, 99% yield). ^1H NMR ($CDCl_3$, 400 MHz) δ 8.09 (s, 1H), 7.62 (d, J = 7.8 Hz, 1H), 7.36 (d, J = 8.1 Hz, 1H), 7.21 (t, J = 8.2 Hz, 1H), 7.16 (t, 2H), 3.80 (d, J = 0.9 Hz, 2H), 3.71 (s, 3H) ppm; ^{13}C NMR ($CDCl_3$, 101 MHz) δ 172.67, 136.23, 127.35, 123.18, 122.38, 119.85, 118.99, 111.32, 108.61, 52.11, 31.29 ppm; LC–MS (ES$^+$) calc'd for $C_{11}H_{12}NO_2^+$ [M+H] 190.0863, found 190.0847. The ^1H and ^{13}C NMR spectra are consistent with previously reported spectra.[37]

Synthesis of methyl 2-(1-acetyl-1*H*-indol-3-yl)acetate (22)

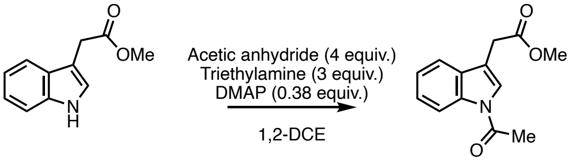

21 **22**

Methyl 2-(1-acetyl-1*H*-indol-3-yl)acetate (**22**) was synthesized based on a published protocol[38] with the following modifications. Methyl 2-(1*H*-indol-3-yl)acetate (**21**) (650 mg, 3.435 mmol, 1 eq.) was dissolved in 1,2-dichloroethene (1,2-DCE) (7 mL). To this solution, acetic anhydride (1.297 mL, 13.744 mmol, 4 eq.), triethylamine (1.437 mL, 10.308 mmol, 3 eq.), and 4-dimethylaminopyridine (DMAP) (160 mg, 1.306 mmol, 0.38 eq.) were added, and the mixture was stirred overnight at room temperature for 22 h. The reaction was washed with a saturated aqueous solution of ammonium chloride (NH_4Cl) and extracted with dichloromethane (DCM). The combined organic extracts were washed with brine, dried over anhydrous Na_2SO_4, and concentrated *in vacuo*. The crude product was purified by column chromatography on silica gel (30:70 ethyl acetate:hexanes) to afford methyl 2-(1-acetyl-1*H*-indol-3-yl)acetate as a yellow oil (786 mg, 3.401 mmol, 99% yield). ^1H NMR ($CDCl_3$, 400 MHz) δ 8.43 (d, J = 8.3 Hz, 1H), 7.52 (d, J = 7.8 Hz, 1H), 7.45 (s, 1H), 7.37 (t, J = 8.4 Hz, 1H), 7.30 (t, 1H), 3.76 – 3.72 (m, 5H), 2.62 (s, 3H) ppm; ^{13}C NMR ($CDCl_3$, 101 MHz) δ 171.74, 168.90, 136.16, 130.49, 125.93, 124.27, 124.11, 119.25, 117.14, 115.35, 52.66, 31.19, 24.41 ppm; LC–MS (ES$^+$) calc'd for $C_{13}H_{14}NO_3^+$ [M+H] 232.0968, found 232.0951.

Synthesis of methyl 2-(1*H*-indol-3-yl)propanoate (23)

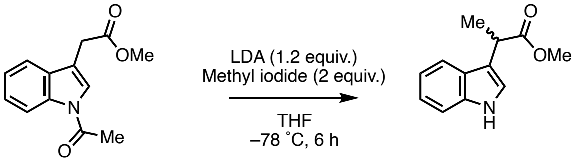

22 **23**

Methyl 2-(1*H*-indol-3-yl)propanoate (**23**) was synthesized according to a published protocol[39] with the following modifications. Methyl 2-(1-acetyl-1*H*-indol-3-yl)acetate (**22**) (420 mg, 1.816 mmol, 1 eq.) was dissolved in dry tetrahydrofuran (THF) (10 mL) in an oven-dried 50 mL flask equipped with a stir bar and cooled to –78 °C. A dry solution of lithium diisopropylamide (1 M in THF/hexanes) (LDA) (2.179 mL, 2.179 mmol, 1.2 eq.) was then added dropwise. The solution was stirred at –78 °C for 30 min before a solution of methyl iodide (0.226 mL, 3.632 mmol, 2 eq.) was added dropwise. The solution was stirred at –78 °C for 30 min before slowly warming up to room temperature and stirred for 6 h. The reaction was quenched with saturated aqueous solution of NH_4Cl and extracted with ethyl acetate (EtOAc). The combined organic extracts were washed with a saturated aqueous solution of $NaHCO_3$, washed with brine, dried over anhydrous Na_2SO_4, and concentrated *in vacuo*. The crude product was purified by column chromatography on silica gel (20:80 ethyl acetate:hexanes) to afford methyl 2-(1*H*-indol-3-yl)propanoate as a yellow oil (131.4 mg, 0.647 mmol, 36% yield). ^{1}H NMR ($CDCl_3$, 400 MHz) δ 8.12 (s, 1H), 7.69 (d, *J* = 8.3 Hz, 1H), 7.35 (d, *J* = 8.1 Hz, 1H), 7.21 (t, *J* = 7.5 Hz, 1H), 7.13 (dd, *J* = 14.3, 6.6 Hz, 2H), 3.68 (s, 3H), 1.63 (d, *J* = 7.2 Hz, 3H) ppm; ^{13}C NMR ($CDCl_3$, 101 MHz) δ 175.84, 136.36, 126.51, 122.32, 121.69, 119.75, 119.29, 115.63, 111.37, 52.09, 37.02, 17.97 ppm; LC–MS (ES$^+$) calc'd for $C_{12}H_{14}NO_2^+$ [M+H] 204.1019, found 204.1012.

Synthesis of α-methyl-indole-3-acetic acid (20)

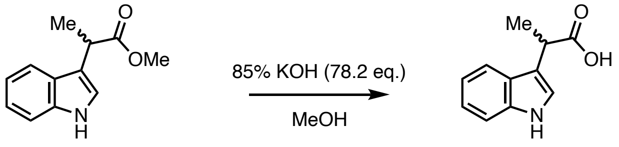

23 **20**

α-Methyl-indole-3-acetic acid (**20**) was synthesized according to a published protocol[39] with the following modifications. Methyl 2-(1*H*-indol-3-yl)propanoate (**23**) (50 mg, 0.246 mmol, 1 eq.) was dissolved in MeOH (24 mL). A solution of potassium hydroxide (KOH) (85%, 1.08 g, 19.249 mmol, 78.2 eq.) in water (6 mL) was added, and the reaction stirred at 70 °C for 1 h. The reaction mixture was cooled to room temperature and the crude was concentrated *in vacuo*. The aqueous solution was acidified with 1 N HCl and extracted with EtOAc. The combined organic extracts were washed with brine, dried over anhydrous Na_2SO_4, and concentrated *in vacuo*. The crude product was purified by preparative thin layer chromatography (1:99 MeOH:DCM) to afford α-methyl-indole-3-acetic acid as a clear oil (104.7 mg, 0.553 mmol, 85% yield). ^{1}H NMR ($CDCl_3$, 400 MHz) δ 8.05 (s, 1H), 7.70 (d, *J* = 7.9 Hz, 1H), 7.35 (d, *J* = 8.0 Hz, 1H), 7.21 (t, *J* = 8.1 Hz, 1H), 7.18 – 7.09 (m, 2H), 4.05 (q, *J* = 7.2 Hz, 1H), 1.63 (d, *J* = 7.2 Hz, 3H) ppm; ^{13}C NMR ($CDCl_3$, 101 MHz) δ 180.94, 136.34, 126.46, 122.46, 121.88, 119.87, 119.40, 115.00, 111.38, 36.99, 17.62 ppm; LC–MS (ES$^+$) calc'd for $C_{11}H_{12}NO_2^+$ [M+H] 190.0863, found 190.0844. The ^{1}H and ^{13}C NMR spectra are consistent with previously reported spectra.[39]

Synthesis of D_3-skatole (24)

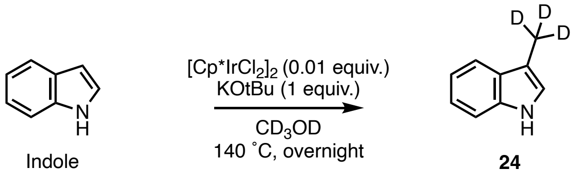

Indole **24**

D_3-Skatole (**24**) was synthesized according to a published protocol[40] with the following modifications. Indole (117.15 mg, 1 mmol, 1.00 eq.) was dissolved in CD_3OD (3.33 mL) in a 25 mL high pressure flask with a Teflon screw top and o-ring and stir bar. To this solution, [Cp*$IrCl_2$]$_2$ (Acros Organics) (7.967 mg, 0.01 mmol, 0.01 eq.) and KO*t*Bu (112.21 mg, 1 mmol, 1.00 eq.) were added. The reaction was stirred in an oil bath for 20 h at 140 °C. After being cooled to ambient temperature, the reaction mixture was concentrated *in vacuo* and purified by column chromatography on silica gel (15:85 ethyl acetate:hexanes) to afford D_3-skatole as a white solid (73.5 mg, 0.548 mmol, 55% yield). ^{1}H NMR ($CDCl_3$, 400 MHz) δ 7.87 (s, 1H), 7.59 (d, *J* = 7.8

Hz, 1H), 7.35 (d, J = 8.1 Hz, 1H), 7.20 (t, J = 8.1 Hz, 1H), 7.13 (t, J = 7.9 Hz, 1H), 6.97 (d, J = 2.2 Hz, 1H) ppm; ^{13}C NMR ($CDCl_3$, 101 MHz) δ 136.39, 128.44, 121.99, 121.69, 119.24, 118.97, 111.74, 111.06, 76.84, 9.62 – 8.30 (m, 1C) ppm; LC–MS (ES$^+$) calc'd for $C_9H_7D_3N^+$ [M+H] 135.0996, found 135.0984. The ^{1}H and ^{13}C NMR spectra are consistent with previously reported spectra.[40]

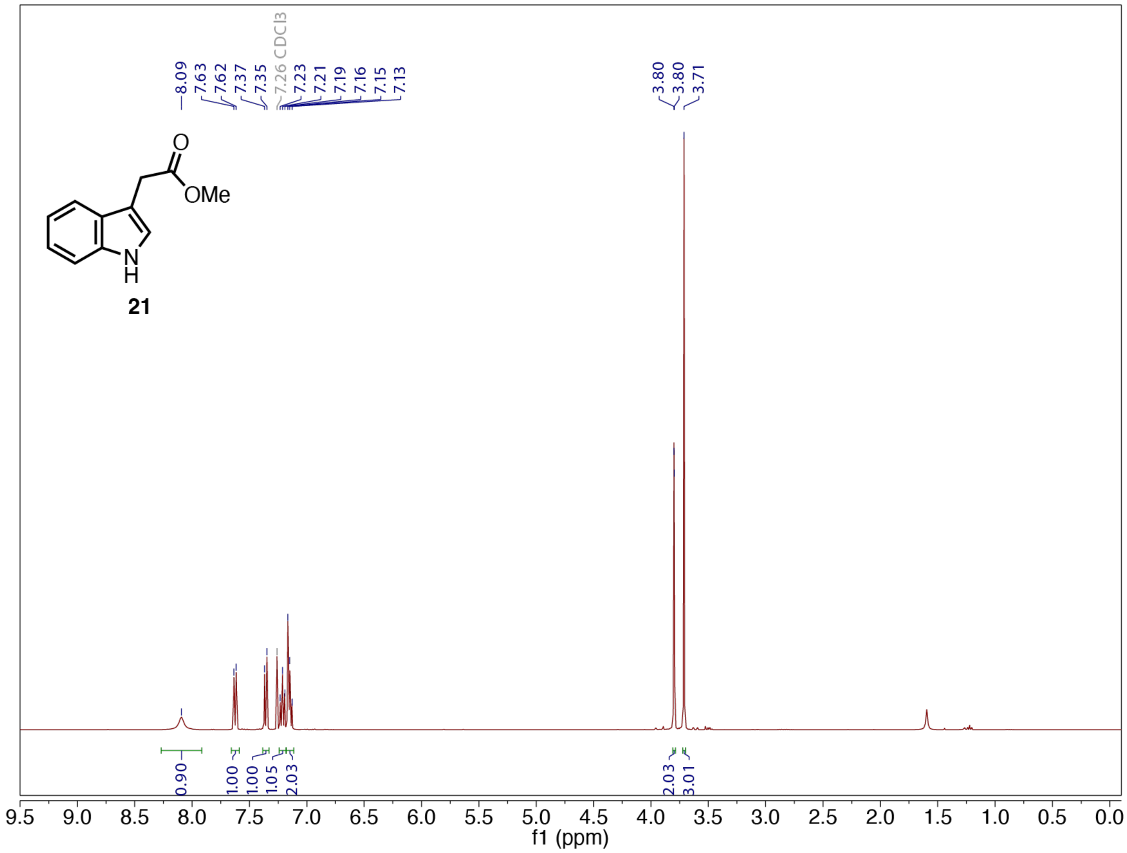
8.09
7.63
7.62
7.37
7.35
7.26 CDCl3
7.23
7.21
7.19
7.16
7.15
7.13
3.80
3.80
3.71
O
OMe
N
H
21
0.90
1.00
1.00
1.05
2.03
2.03
3.01
9.5 9.0 8.5 8.0 7.5 7.0 6.5 6.0 5.5 5.0 4.5 4.0 3.5 3.0 2.5 2.0 1.5 1.0 0.5 0.0
f1 (ppm)

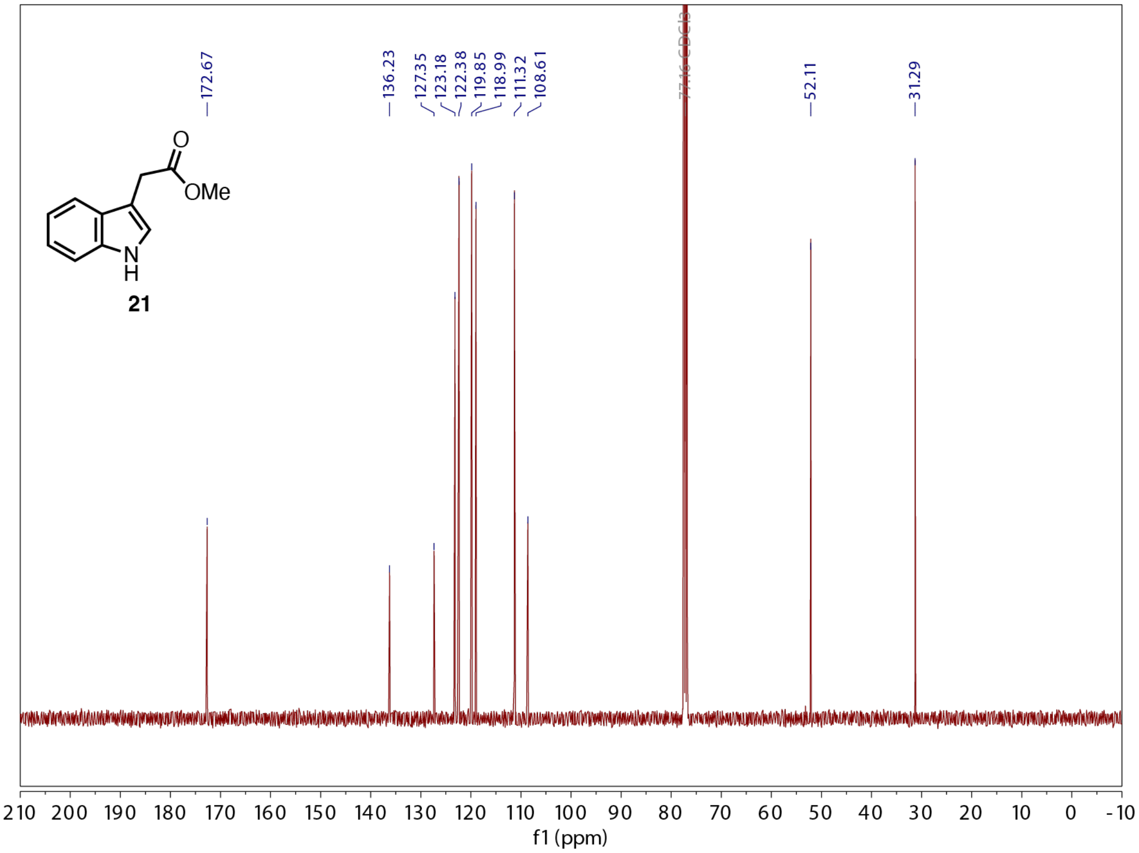
172.67
136.23
127.35
123.18
122.38
119.85
118.99
111.32
108.61
77.16 CDCl3
52.11
31.29
O
OMe
N
H
21
210 200 190 180 170 160 150 140 130 120 110 100 90 80 70 60 50 40 30 20 10 0 -10
f1 (ppm)

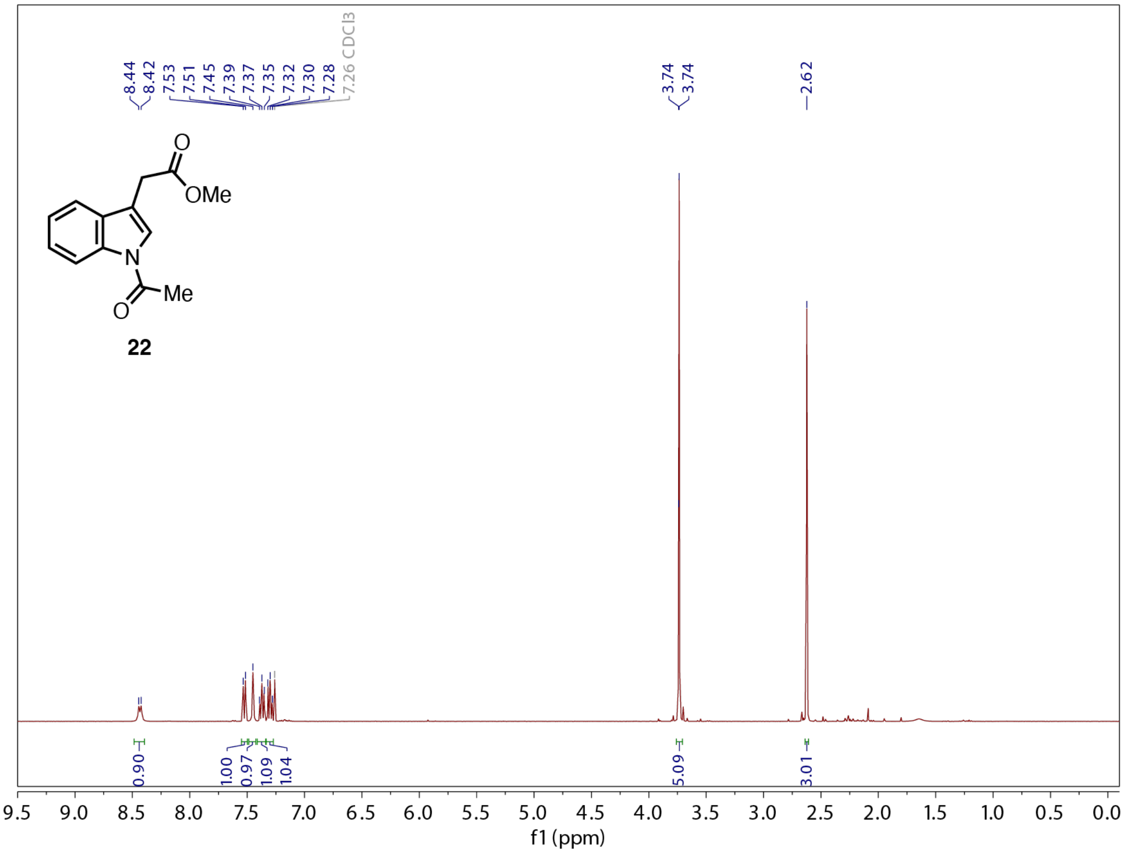
8.44
8.42
7.53
7.51
7.45
7.39
7.37
7.35
7.32
7.30
7.28
7.26 CDCl3
3.74
3.74
2.62
O
OMe
N
O
Me
22
0.90
1.00
0.97
1.09
1.04
5.09
3.01
9.5 9.0 8.5 8.0 7.5 7.0 6.5 6.0 5.5 5.0 4.5 4.0 3.5 3.0 2.5 2.0 1.5 1.0 0.5 0.0
f1 (ppm)

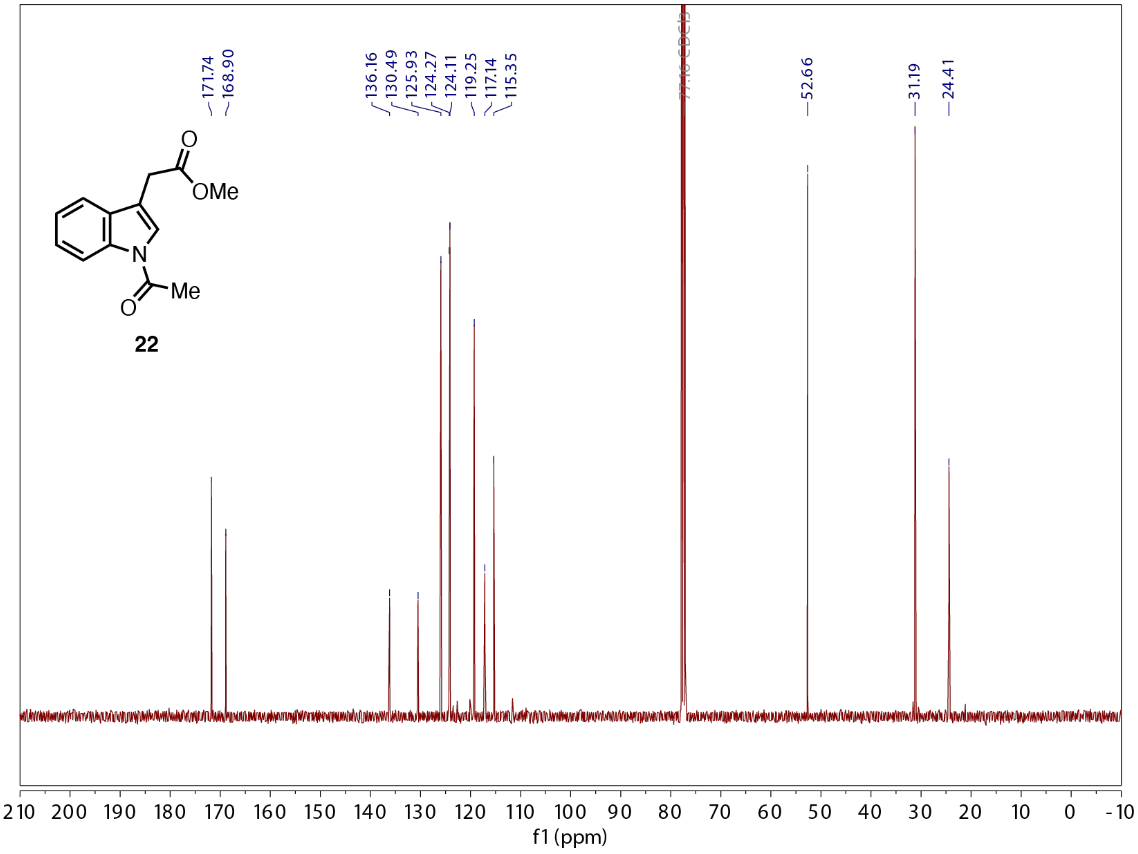
171.74
168.90
136.16
130.49
125.93
124.27
124.11
119.25
117.14
115.35
52.66
31.19
24.41
O
OMe
N
O
Me
22
210 200 190 180 170 160 150 140 130 120 110 100 90 80 70 60 50 40 30 20 10 0 -10
f1 (ppm)

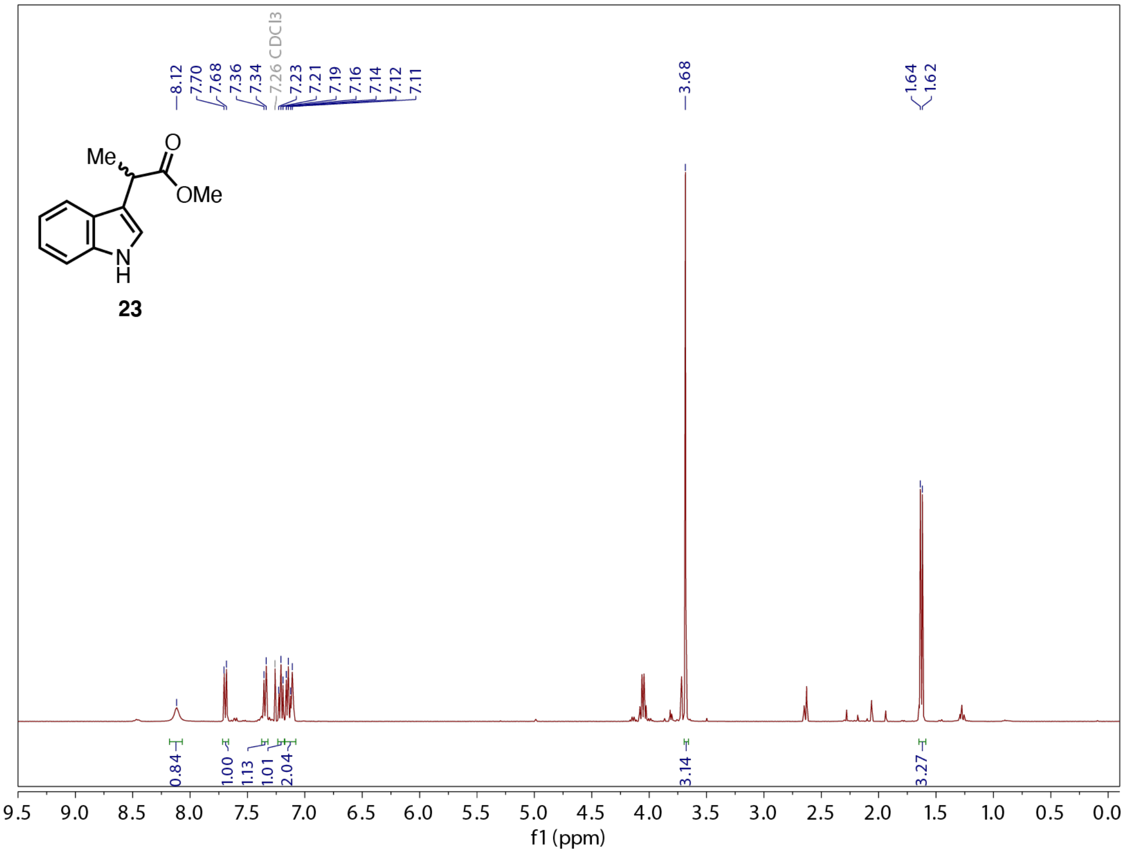

8.12
7.70
7.68
7.36
7.34
7.26 CDCl3
7.23
7.21
7.19
7.16
7.14
7.12
7.11
3.68
1.64
1.62
Me
O
OMe
N
H
23
0.84
1.00
1.13
1.01
2.04
3.14
3.27
f1 (ppm)

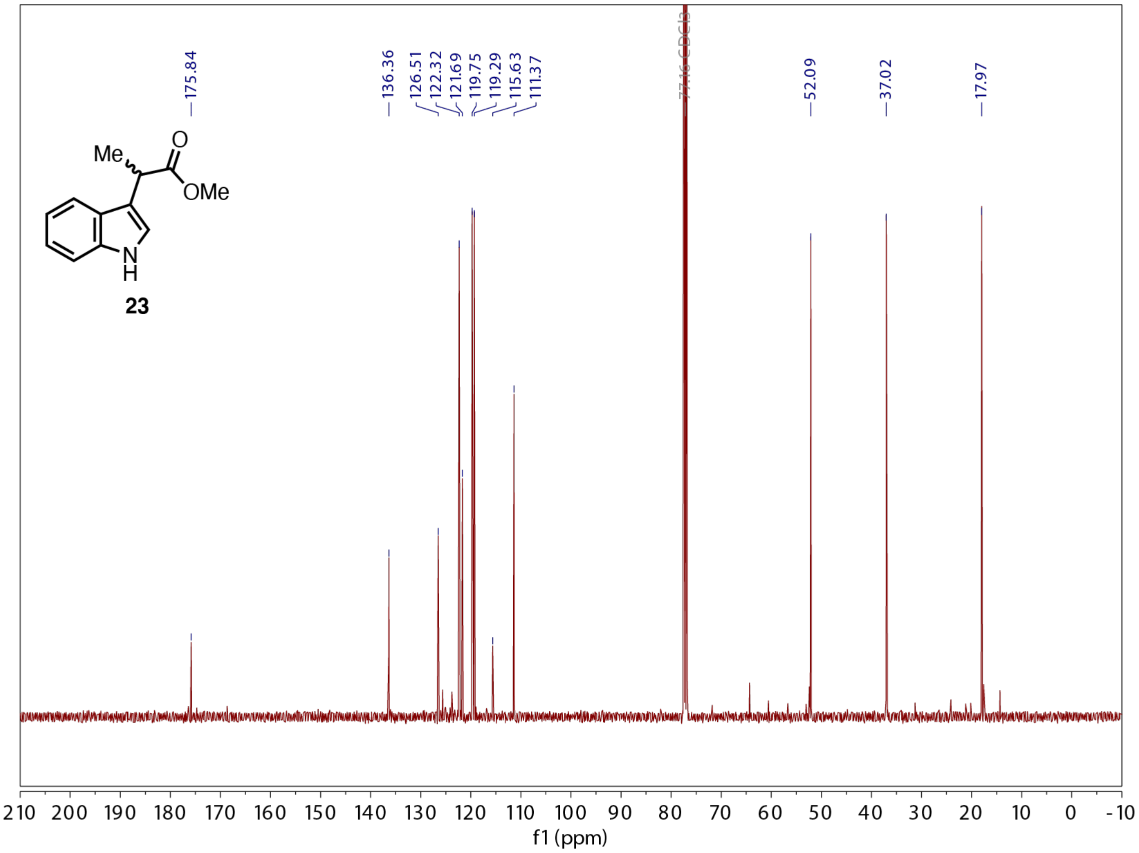

175.84
136.36
126.51
122.32
121.69
119.75
119.29
115.63
111.37
77.16 CDCl3
52.09
37.02
17.97
Me
O
OMe
N
H
23
f1 (ppm)

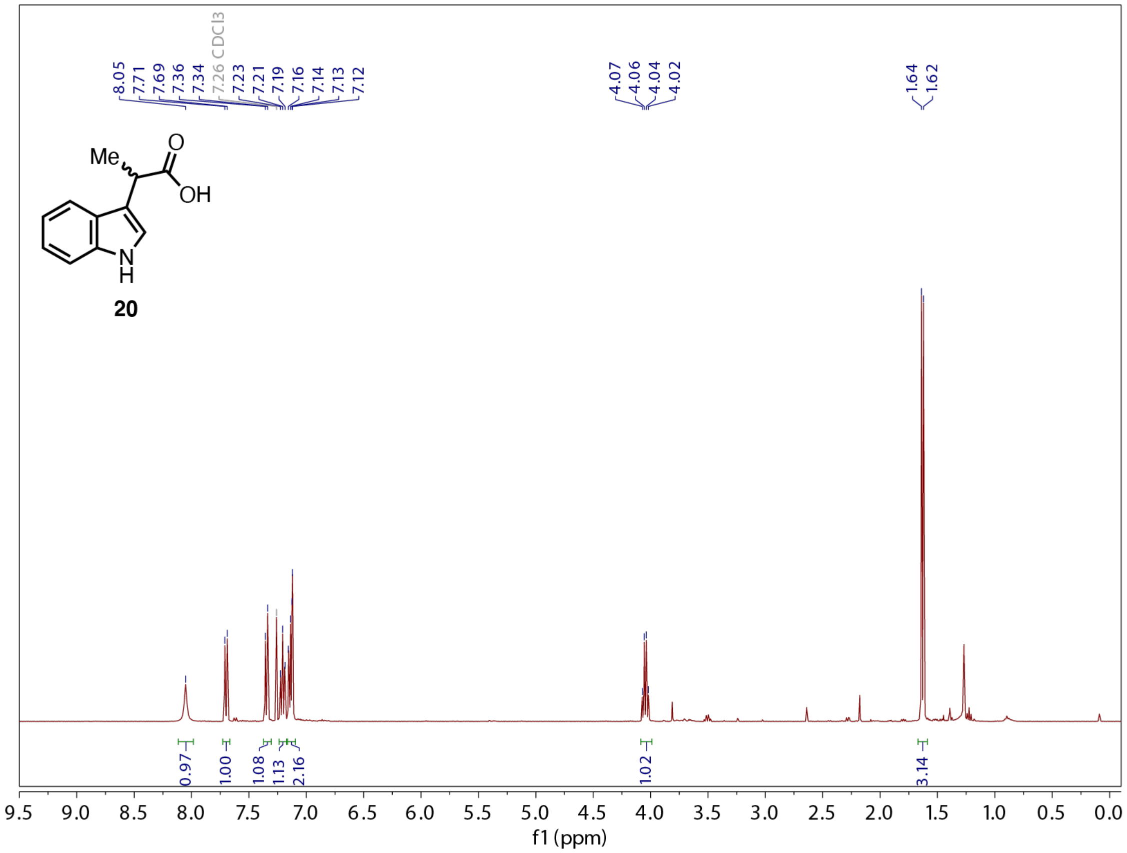
8.05
7.71
7.69
7.36
7.34
7.26 CDCl3
7.23
7.21
7.19
7.16
7.14
7.13
7.12
4.07
4.06
4.04
4.02
1.64
1.62
Me
O
OH
N
H
20
0.97
1.00
1.08
1.13
2.16
1.02
3.14
9.5 9.0 8.5 8.0 7.5 7.0 6.5 6.0 5.5 5.0 4.5 4.0 3.5 3.0 2.5 2.0 1.5 1.0 0.5 0.0
f1 (ppm)

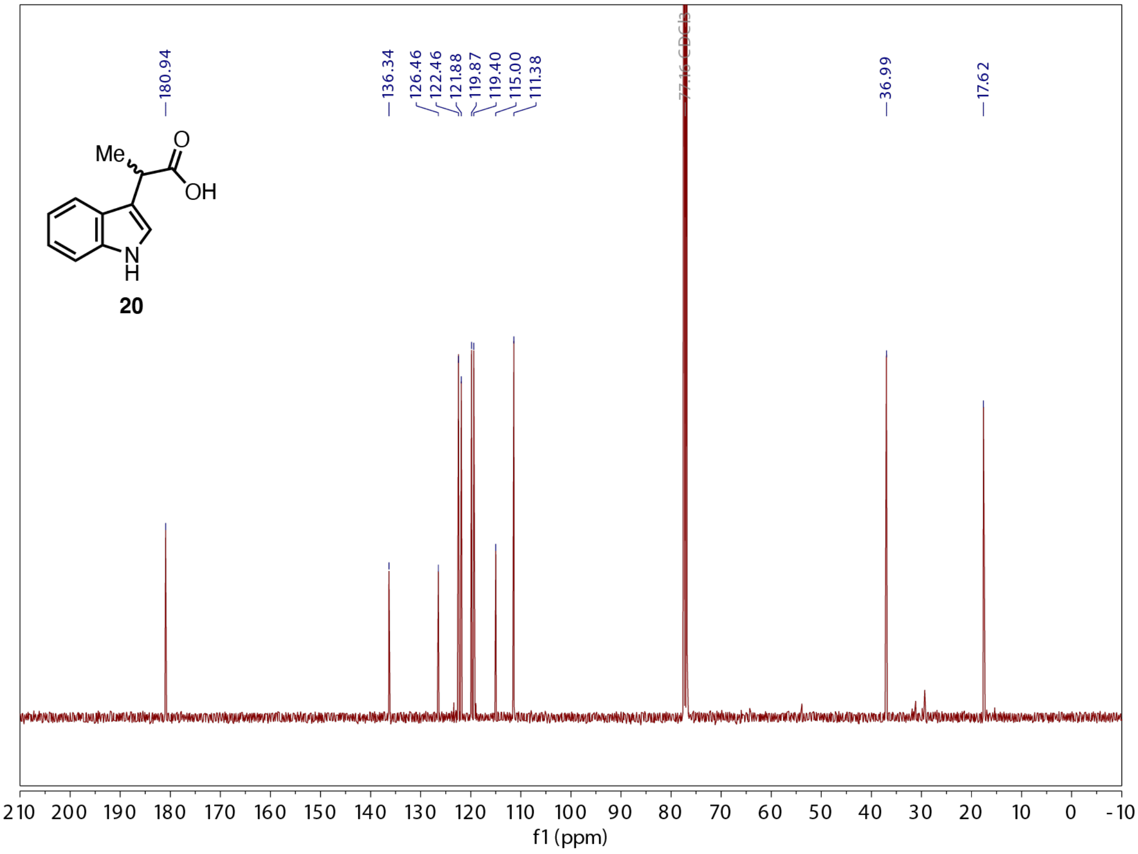
180.94
136.34
126.46
122.46
121.88
119.87
119.40
115.00
111.38
77.16 CDCl3
36.99
17.62
Me
O
OH
N
H
20
210 200 190 180 170 160 150 140 130 120 110 100 90 80 70 60 50 40 30 20 10 0 -10
f1 (ppm)

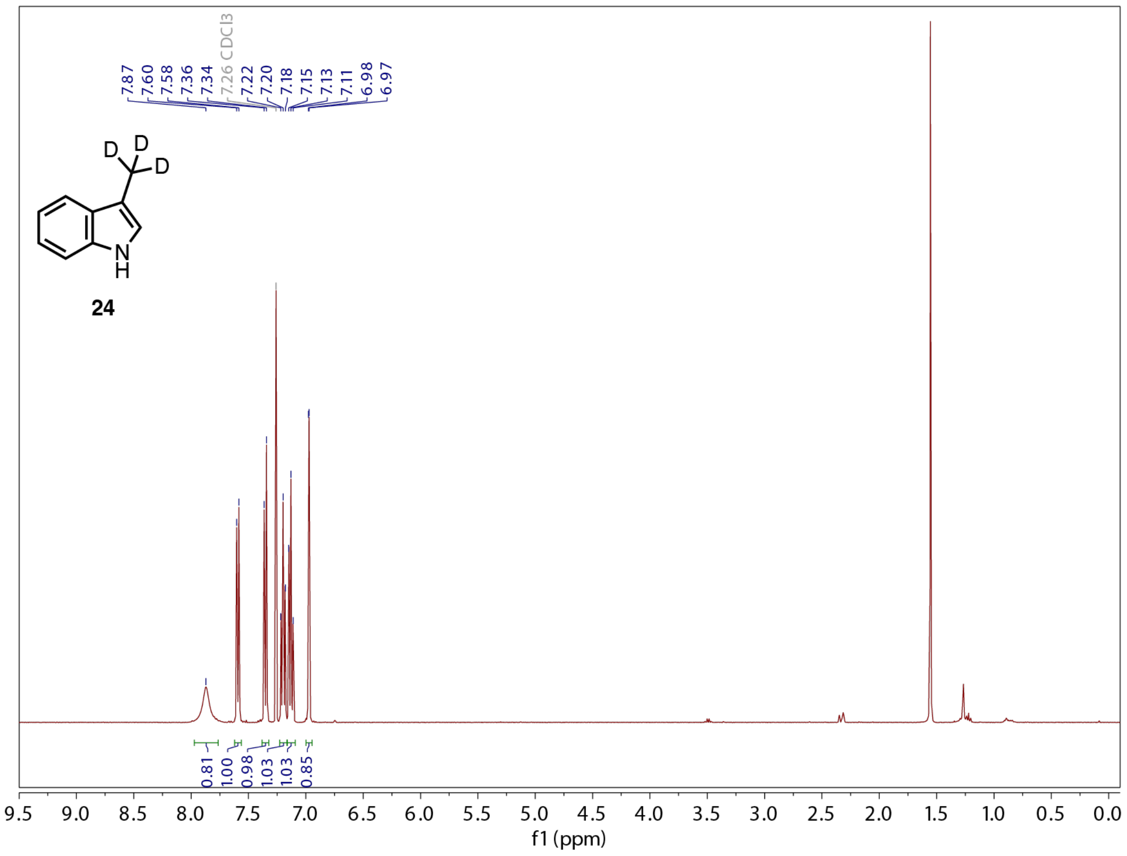
7.87
7.60
7.58
7.36
7.34
7.26 CDCl3
7.22
7.20
7.18
7.15
7.13
7.11
6.98
6.97
D
D
D
N
H
24
0.81
1.00
0.98
1.03
1.03
0.85
9.5 9.0 8.5 8.0 7.5 7.0 6.5 6.0 5.5 5.0 4.5 4.0 3.5 3.0 2.5 2.0 1.5 1.0 0.5 0.0
f1 (ppm)

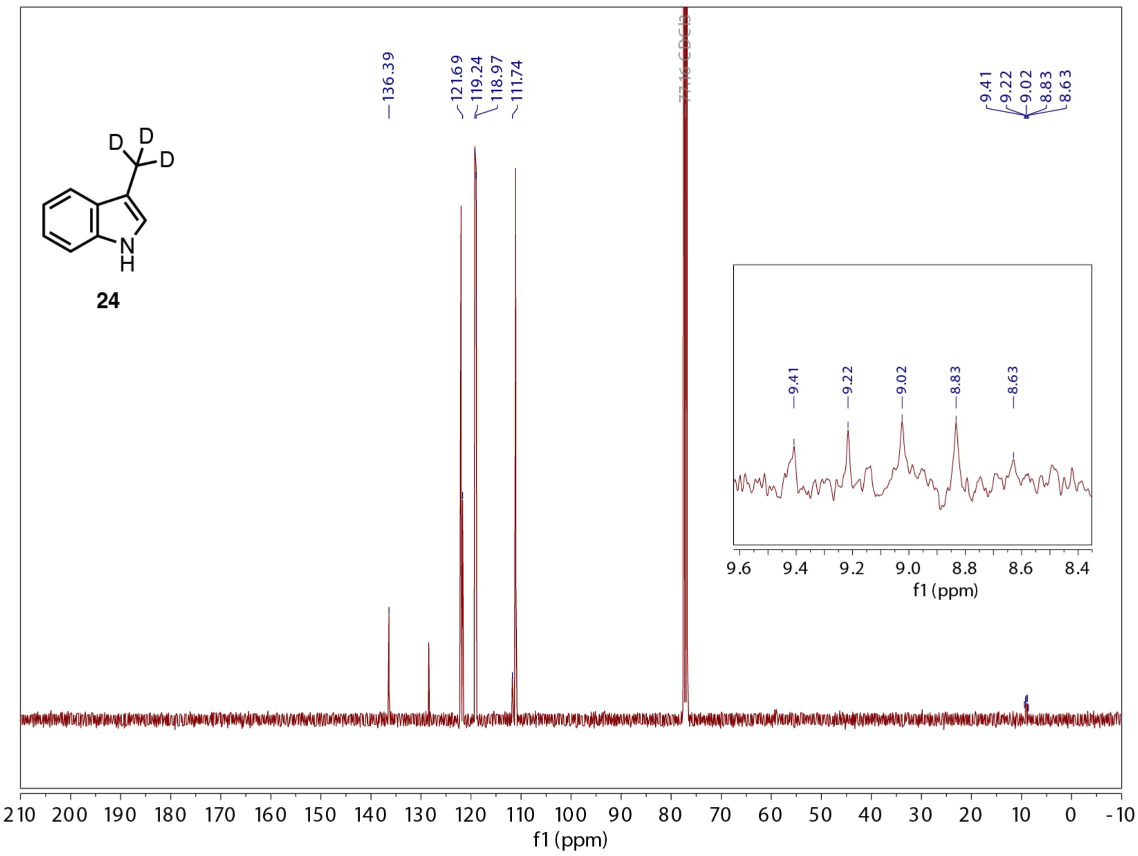
136.39
121.69
119.24
118.97
111.74
77.16 CDCl3
9.41
9.22
9.02
8.83
8.63
D
D
D
N
H
24
9.6 9.4 9.2 9.0 8.8 8.6 8.4
f1 (ppm)
210 200 190 180 170 160 150 140 130 120 110 100 90 80 70 60 50 40 30 20 10 0 -10
f1 (ppm)

Supplemental references

(1) Waterhouse, A.; Bertoni, M.; Bienert, S.; Studer, G.; Tauriello, G.; Gumienny, R.; Heer, F. T.; Beer, D., P, Tjaart A; Rempfer, C.; Bordoli, L.; Lepore, R.; Schwede, T. SWISS-MODEL: homology modelling of protein structures and complexes. *Nucleic Acids Res.* **2018**, *46*, W296–W303.
(2) Jumper, J.; Evans, R.; Pritzel, A.; Green, T.; Figurnov, M.; Ronneberger, O.; Tunyasuvunakool, K.; Bates, R.; Žídek, A.; Potapenko, A.; Bridgland, A.; Meyer, C.; Kohl, S. A. A.; Ballard, A. J.; Cowie, A.; Romera-Paredes, B.; Nikolov, S.; Jain, R.; Adler, J.; Back, T.; Petersen, S.; Reiman, D.; Clancy, E.; Zielinski, M.; Steinegger, M.; Pacholska, M.; Berghammer, T.; Bodenstein, S.; Silver, D.; Vinyals, O.; Senior, A. W.; Kavukcuoglu, K.; Kohli, P.; Hassabis, D. Highly accurate protein structure prediction with AlphaFold. *Nature* **2021**, *596*, 583–589.
(3) Mirdita, M.; Schütze, K.; Moriwaki, Y.; Heo, L.; Ovchinnikov, S.; Steinegger, M. ColabFold - making protein folding accessible to all. *bioRxiv* **2021**.
(4) Bodea, S.; Funk, M. A.; Balskus, E. P.; Drennan, C. L. Molecular basis of C–N bond cleavage by the glycyl radical enzyme choline trimethylamine-lyase. *Cell Chem. Biol.* **2016**, *23*, 1206–1216.
(5) Levin, B. J.; Huang, Y. Y.; Peck, S. C.; Wei, Y.; Martínez-del Campo, A.; Marks, J. A.; Franzosa, E. A.; Huttenhower, C.; Balskus, E. P. A prominent glycyl radical enzyme in human gut microbiomes metabolizes *trans*-4-hydroxy-L-proline. *Science* **2017**, *355*, eaai8386.
(6) Knappe, J.; Blaschkowski, H. P.; Grobner, P.; Schmttt, T. Pyruvate formate-lyase of *Escherichia coli*: the acetyl-enzyme intermediate. *Eur. J. Biochem.* **1974**, *50*, 253–263.
(7) Liu, J.; Wei, Y.; Lin, L.; Teng, L.; Yin, J.; Lu, Q.; Chen, J.; Zheng, Y.; Li, Y.; Xu, R.; Zhai, W.; Liu, Y.; Liu, Y.; Cao, P.; Ang, E. L.; Zhao, H.; Yuchi, Z.; Zhang, Y. Two radical-dependent mechanisms for anaerobic degradation of the globally abundant organosulfur compound dihydroxypropanesulfonate. *Proc. Natl. Acad. Sci.* **2020**, *117*, 15599–15608.
(8) Dawson, C. D.; Irwin, S. M.; Backman, L. R. F.; Le, C.; Wang, J. X.; Vennelakanti, V.; Yang, Z.; Kulik, H. J.; Drennan, C. L.; Balskus, E. P. Molecular basis of C–S bond cleavage in the glycyl radical enzyme isethionate sulfite-lyase. *Cell Chem. Biol.* **2021**, *28*, 1333–1346.
(9) Peck, S. C.; Denger, K.; Burrichter, A.; Irwin, S. M.; Balskus, E. P.; Schleheck, D. A glycyl radical enzyme enables hydrogen sulfide production by the human intestinal bacterium *Bilophila wadsworthia*. *Proc. Natl. Acad. Sci.* **2019**, *116*, 3171–3176.
(10) Xing, M.; Wei, Y.; Zhou, Y.; Zhang, J.; Lin, L.; Hu, Y.; Hua, G.; N. Nanjaraj Urs, A.; Liu, D.; Wang, F.; Guo, C.; Tong, Y.; Li, M.; Liu, Y.; Ang, E. L.; Zhao, H.; Yuchi, Z.; Zhang, Y. Radical-mediated C–S bond cleavage in C2 sulfonate degradation by anaerobic bacteria. *Nat. Commun.* **2019**, *10*, 1–11.
(11) Yu, L.; Blaser, M.; Andrei, P. I.; Pierik, A. J.; Selmer, T. 4-Hydroxyphenylacetate decarboxylases: Properties of a novel subclass of glycyl radical enzyme systems. *Biochemistry* **2006**, *45*, 9584–9592.
(12) Selmer, T.; Andrei, P. I. *p*-Hydroxyphenylacetate decarboxylase from *Clostridium difficile*. *Eur. J. Biochem.* **2001**, *268*, 1363–1372.
(13) Rodrigues, A. V.; Tantillo, D. J.; Mukhopadhyay, A.; Keasling, J. D.; Beller, H. R. Insight into the mechanism of phenylacetate decarboxylase (PhdB), a toluene-producing glycyl radical enzyme. *ChemBioChem* **2020**, *21*, 663–671.
(14) Lu, Q.; Wei, Y.; Lin, L.; Liu, J.; Duan, Y.; Li, Y.; Zhai, W.; Liu, Y.; Ang, E. L.; Zhao, H.; Yuchi, Z.; Zhang, Y. The glycyl radical enzyme arylacetate decarboxylase from *Olsenella scatoligenes*. *ACS Catal.* **2021**, *11*, 5789–5794.
(15) Liu, D.; Wei, Y.; Liu, X.; Zhou, Y.; Jiang, L.; Yin, J.; Wang, F.; Hu, Y.; Nanjaraj Urs, A. N.; Liu, Y.; Lui Ang, E.; Zhao, S.; Zhao, H.; Zhang, Y.; Ang, E. L.; Zhao, S.; Zhao, H.; Zhang, Y. Indoleacetate decarboxylase is a glycyl radical enzyme catalysing the formation of malodorant skatole. *Nat. Commun.* **2018**, *9*, 4224.
(16) Kearse, M.; Moir, R.; Wilson, A.; Stones-Havas, S.; Cheung, M.; Sturrock, S.; Buxton, S.; Cooper, A.; Markowitz, S.; Duran, C.; Thierer, T.; Ashton, B.; Meintjes, P.; Drummond, A. Geneious Basic: an integrated and extendable desktop software platform for the organization and analysis of sequence data. *Bioinformatics* **2012**, *28*, 1647–1649.

(17) Sievers, F.; Wilm, A.; Dineen, D.; Gibson, T. J.; Karplus, K.; Li, W.; Lopez, R.; McWilliam, H.; Remmert, M.; Soding, J.; Thompson, J. D.; Higgins, D. G. Fast, scalable generation of high-quality protein multiple sequence alignments using Clustal Omega. *Mol. Syst. Biol.* **2014**, *7*, 539–539.
(18) Li, X.; Højberg, O.; Noel, S. J.; Canibe, N.; Jensen, B. B. Draft genome sequence of *Olsenella scatoligenes* SK9K4^{T}, a producer of 3-methylindole (skatole) and 4-methylphenol (*p*-cresol), isolated from pig feces. *Genome Announc.* **2016**, *4*, e00042-16.
(19) Göker, M.; Held, B.; Lucas, S.; Nolan, M.; Yasawong, M.; Glavina Del Rio, T.; Tice, H.; Cheng, J.-F.; Bruce, D.; Detter, J. C.; Tapia, R.; Han, C.; Goodwin, L.; Pitluck, S.; Liolios, K.; Ivanova, N.; Mavromatis, K.; Mikhailova, N.; Pati, A.; Chen, A.; Palaniappan, K.; Land, M.; Hauser, L.; Chang, Y.-J.; Jeffries, C. D.; Rohde, M.; Sikorski, J.; Pukall, R.; Woyke, T.; Bristow, J.; Eisen, J. A.; Markowitz, V.; Hugenholtz, P.; Kyrpides, N. C.; Klenk, H.-P.; Lapidus, A. Complete genome sequence of *Olsenella uli* type strain (VPI D76D-27C^{T}). *Stand. Genom. Sci.* **2010**, *3*, 76–84.
(20) Gibson, D. G.; Young, L.; Chuang, R.-Y.; Venter, J. C.; Hutchison, C. A.; Smith, H. O. Enzymatic assembly of DNA molecules up to several hundred kilobases. *Nat. Methods* **2009**, *6*, 343–345.
(21) Gasteiger, E.; Hoogland, C.; Gattiker, A.; Duvaud, S. E.; Wilkins, M. R.; Appel, R. D.; Bairoch, A., Protein identification and analysis tools on the ExPASy server. In *The Proteomics Protocols Handbook*, Humana Press: 2005; pp 571–607.
(22) Lanz, N. D.; Grove, T. L.; Gogonea, C. B.; Lee, K.-H.; Krebs, C.; Booker, S. J. RlmN and AtsB as models for the overproduction and characterization of radical SAM proteins. *Methods Enzymol.* **2012**, *516*, 125–152.
(23) Carlson, E. E.; Kiessling, L. L. Improved chemical syntheses of 1- and 5-deazariboflavin. *J. Org. Chem.* **2004**, *69*, 2614–2617.
(24) Henshaw, T. F.; Cheek, J.; Broderick, J. B. The [4Fe-4S]$^{1+}$ cluster of pyruvate formate-lyase activating enzyme generates the glycyl radical on pyruvate formate-lyase: EPR-detected single turnover. *J. Am. Chem. Soc.* **2000**, *122*, 8331–8332.
(25) Goldman, S. A.; Bruno, G. V.; Polnaszek, C. F.; Freed, J. H. An ESR study of anisotropic rotational reorientation and slow tumbling in liquid and frozen media. *J. Chem. Phys.* **1972**, *56*, 716–735.
(26) Stoll, S.; Schweiger, A. EasySpin, a comprehensive software package for spectral simulation and analysis in EPR. *J. Magn. Reson.* **2006**, *178*, 42–55.
(27) Hamoodi Murib, J.; Ritter, D. M.; Hamoodi Murib, B. J. Decomposition of nitrosyl disulfonate ion. I. Products and mechanism of color fading in acid solution. *J. Am. Chem. Soc.* **1952**, *74*, 3394–3398.
(28) Kennedy, M. C.; Kent, T. A.; Emptage, M.; Merkle, H.; Beinert, H.; Münck, E. Evidence for the formation of a linear [3Fe-4S] cluster in partially unfolded aconitase. *J. Biol. Chem.* **1984**, *259*, 14463–14471.
(29) Beinert, H. Semi-micro methods for analysis of labile sulfide and of labile sulfide plus sulfane sulfur in unusually stable iron-sulfur proteins. *Anal. Biochem.* **1983**, *131*, 373–378.
(30) Singh, P.; Islam, Z.; Kohen, A. Examinations of the chemical step in enzyme catalysis. *Methods Enzymol.* **2016**, 287–318.
(31) Neese, F. The ORCA program system. *WIREs Comput. Mol. Sci.* **2012**, *2*, 73–78.
(32) Becke, A. D. Density-functional thermochemistry. III. The role of exact exchange. *J. Chem. Phys.* **1993**, *98*, 5648–5652.
(33) Riplinger, C.; Neese, F. An efficient and near linear scaling pair natural orbital based local coupled cluster method. *J. Chem. Phys.* **2013**, *138*, 034106.
(34) Liu, F.; Luehr, N.; Kulik, H. J.; Martínez, T. J. Quantum chemistry for solvated molecules on graphical processing units using polarizable continuum models. *J. Chem. Theory Comput.* **2015**, *11*, 3131–3144.
(35) Klamt, A.; Schüürmann, G. COSMO: a new approach to dielectric screening in solvents with explicit expressions for the screening energy and its gradient. *J. Chem. Soc., Perkin Trans. 2* **1993**, 799–805.
(36) Møller, C.; Plesset, M. S. Note on an approximation treatment for many-electron systems. *Phys. Rev.* **1934**, *46*, 618–622.
(37) Tsui, K. Y.; Tombari, R. J.; Olson, D. E.; Tantillo, D. J. Reconsidering the structure of Serlyticin-A. *J. Nat. Prod.* **2019**, *82*, 3464–3468.
(38) Medina-Mercado, I.; Asomoza-Solís, E. O.; Martínez-González, E.; Ugalde-Saldívar, V. M.; Ledesma-Olvera, L. G.; Barquera-Lozada, J. E.; Gómez-Vidales, V.; Barroso-Flores, J.; Frontana-Uribe, B. A.; Porcel,

S. Ascorbic acid as an aryl radical inducer in the gold-mediated arylation of indoles with aryldiazonium chlorides. *Chem. Eur. J.* **2020**, *26*, 634–642.
(39) Katayama, M.; Kato, Y.; Marumo, S. Synthesis, absolute configuration and biological activity of both enantiomers of 2-(5,6-dichloro-3-indolyl)propionic acid: new dichloroindole auxins. *Biosci. Biotechnol. Biochem.* **2001**, *65*, 270–276.
(40) Chen, S.-J.; Lu, G.-P.; Cai, C. Iridium-catalyzed methylation of indoles and pyrroles using methanol as feedstock. *RSC Adv.* **2015**, *5*, 70329–70332.
